# Bioinspired Syntheses of Dimeric Hydroxycinnamic Acids (Lignans) and Hybrids, Using Phenol Oxidative Coupling as Key Reaction, and Medicinal Significance Thereof †

**DOI:** 10.3390/molecules191219769

**Published:** 2014-11-28

**Authors:** George E. Magoulas, Dionissios Papaioannou

**Affiliations:** Department of Chemistry, University of Patras, Patras 26504, Greece; E-Mail: magoulas@upatras.gr

**Keywords:** 4-hydroxycinnamic acids, lignans, biomimetic synthesis, phenol oxidative coupling, regioselective coupling, stereoselective coupling, dilactones, dihydronaphthalenes, dihydrobenzofurans, hybrids

## Abstract

Lignans are mainly dimers of 4-hydroxycinnamic acids (HCAs) and reduced analogs thereof which are produced in Nature through phenol oxidative coupling (POC) as the primary C-C or C-O bond-forming reaction under the action of the enzymes peroxidases and laccases. They present a large structural variety and particularly interesting biological activities, therefore, significant efforts has been devoted to the development of efficient methodologies for the synthesis of lignans isolated from natural sources, analogs and hybrids with other biologically interesting small molecules. We summarize in the present review those methods which mimic Nature for the assembly of the most common lignan skeleta by using either enzymes or one-electron inorganic oxidants to effect POC of HCAs and derivatives, such as esters and amides, or cross-POC of pairs of HCAs or HCAs with 4-hydrocycinnamyl alcohols. We, furthermore, provide outlines of mechanistic schemes accounting for the formation of the coupled products and, where applicable, indicate their potential application in medicine.

## 1. Introduction

**Scheme 1:**
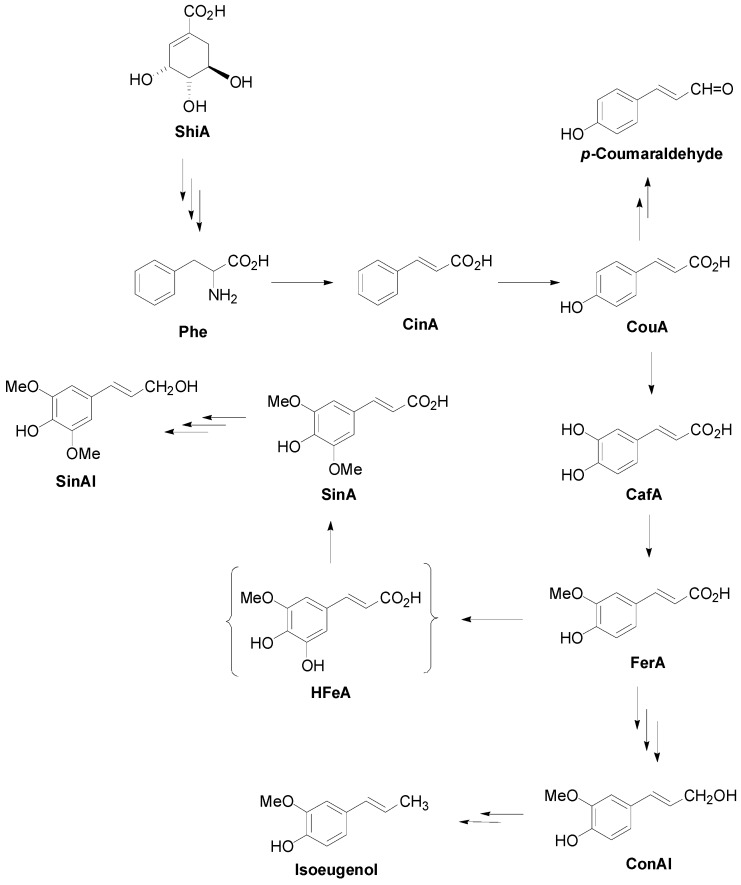
Outline of the biosynthetic route to naturally occurring cinnamates and selected reduced derivatives.

CinA and its 4-hydroxysubstituted derivatives (HCAs), namely CouA, CafA, FerA and SinA, form an important family of natural products ubiquitous in the plant kingdom generally known as cinnamates [1]. Cinnamates are derived biogenetically from ShiA through the intermediacy of the amino acid Phe (Scheme 1).

HCAs and derivatives are powerful natural antioxidants, abundantly found in a variety of foods and drinks, which could be therefore used for the prevention and/or therapy of oxidative stress-associated diseases, like atherosclerosis, inflammatory injury and cancer [2,3]. The side chain of cinnamates is biogenetically further reduced thus leading to a variety of reduced derivatives such as the corresponding aldehydes (e.g., *p*-coumaraldehyde), alcohols (e.g., ConAl) and alkenes (e.g., isoeugenol). All these compounds are coined as phenylpropanoids and form the C_6_-C_3_ class of ShiA metabolites [1].

Interestingly, these phenypropanoid compounds are further dimerized or polymerized in Nature and thus provide access to two further families of natural products also widely distributed in plants, collectively coined as lignans and lignin, respectively. Lignans are mainly dimeric in Nature and are produced biosynthetically by the dimerization of HCAs and their reduced derivatives HCAls. The role of lignans in plants seems to be primarily plant defense [4]. Lignin is a high molecular weight complex polymer, derived from the polymerization of phenylpropanoid alcohols (called monolignols), such as ConAl. Lignin constitutes an integral part of the secondary cell walls of plants conferring mechanical strength to the cell wall and, by extension, to plant as a whole.

**Scheme 2:**
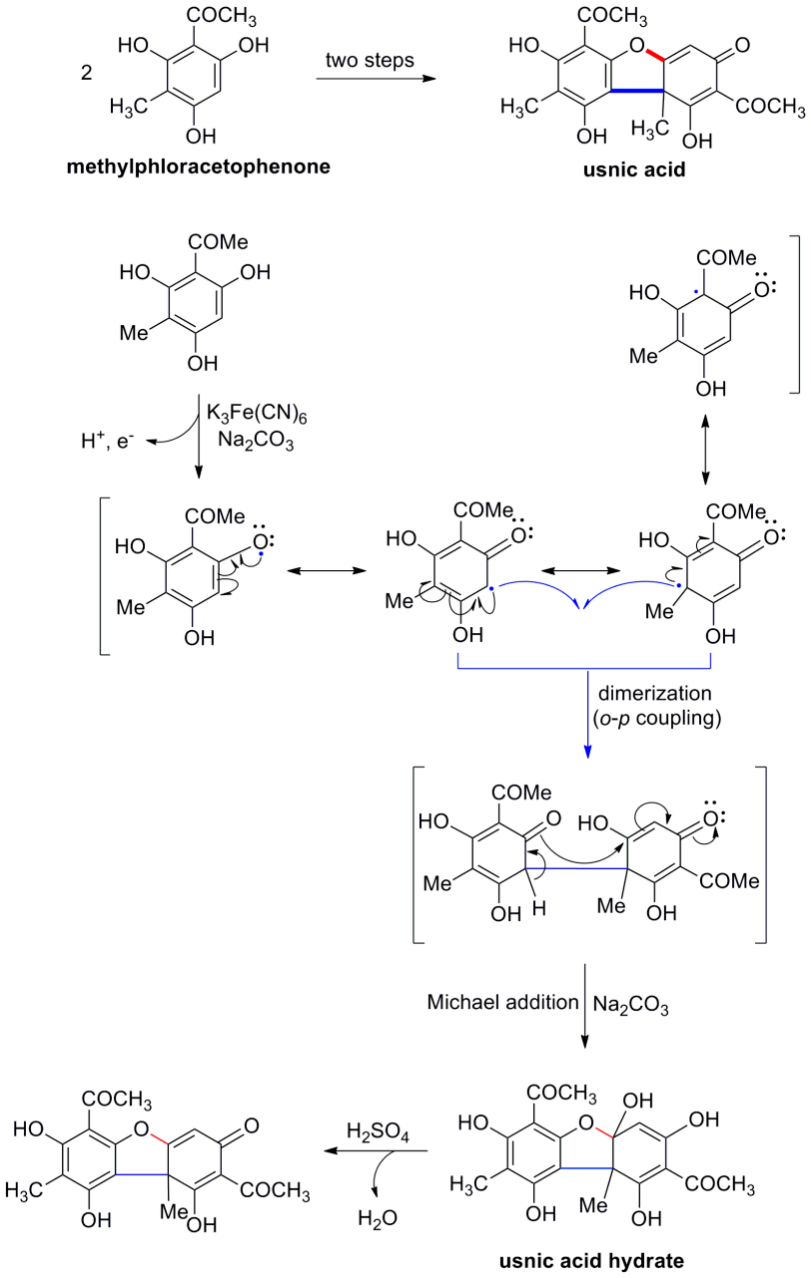
Barton’s biomimetic synthesis of usnic acid based on POC as key step. The primary bond connecting the two monomers is shown in blue and bolded and the secondary one in red and bolded.

Dimerization of phenolic compounds in Nature is effected using POC as key reaction, which allows the connection of the monomers through the formation of new C-C or C-O bonds. Although this concept had been long recognized, it was the seminal investigation by Barton and Cohen on the structure of Pummerer’s ketone, produced by the ferricyanide-mediated POC of *p*-cresol in alkaline solution, which established the relation of such reactions in the laboratory with biosynthetic pathways [5]. This concept was applied in the elegant biomimetic two-steps synthesis of (±)-usnic acid by Barton and coworkers using the POC of methylphloracetophenone as key step for the assembly of the skeleton (Scheme 2) [6].

According to the mechanism put forward, one-electron oxidation of the corresponding phenolate leads to a radical species which, due to the resonance effect exhibits additional mesomeric forms, with the unpaired electron residing on the *ortho* and *para* carbons. Radical pairing creates a new C-C bond connecting the two monomers and the thus obtained intermediate, following aromatization, undergoes a base-induced intramolecular Michael addition thus producing the tricyclic usnic acid hydrate in one pot. Dehydration may then be effected directly by treating with concentrated sulfuric acid or indirectly by treating with acetic anhydride in the presence of a catalytic quantity of sulfuric acid followed by sulfuric acid-mediated hydrolysis of the thus obtained usnic acid diacetate.

**Scheme 3:**
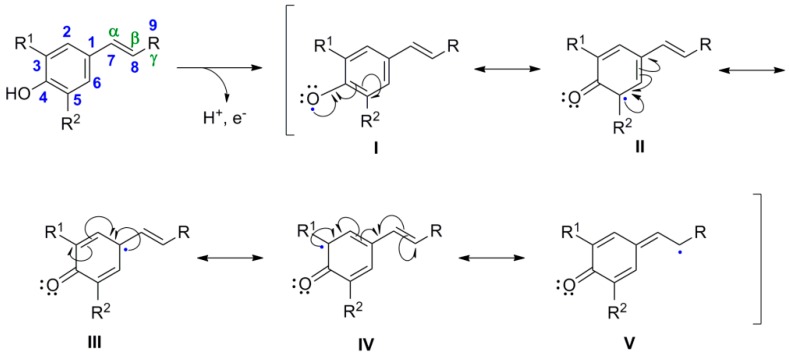
One electron oxidation of 4-hydroxyphenylpropanoid compounds creating the mesomeric forms **I**–**V** of the phenoxide redical. Numbering of the aromatic ring and the alternative numbering of the side-chain is the one suggested by IUPAC.

Although radical pairing seems to be the prevailing mechanism in POC, other mechanisms might be applicable in certain cases, e.g., two electron oxidation of one phenolate to create an onium ion followed by an electrophilic aromatic substitution on the other electron-rich phenolate, also followed by a prototropic shift leading to aromatization, in case one or both of the coupled carbon atoms bear a hydrogen atom. If this were not the case, external or better internal nucleophiles attack the intermediate cross-conjugated spirodienone or a dienone-phenol type rearrangement takes place creating stable products, which can further lose small molecule, e.g. water, to give the final product [1]. POC is also the key reaction through which the plethora of the naturally occurring lignans is biosynthetically produced from monomeric phenylpropanoid compounds [7,8,9,10,11,12,13], under the action of the enzymes peroxidases or laccases. The two families of enzymes mainly differ in the metallic cation present in their active site, Fe^3+^ in the former and Cu^2+^ in the latter. Due to their side chain, one electron oxidation of phenolate anions of phenylpropanoids creates through resonance an additional site (C-8 or C-β) for radical coupling (see canonical form **V** in Scheme 3).

**Scheme 4:**
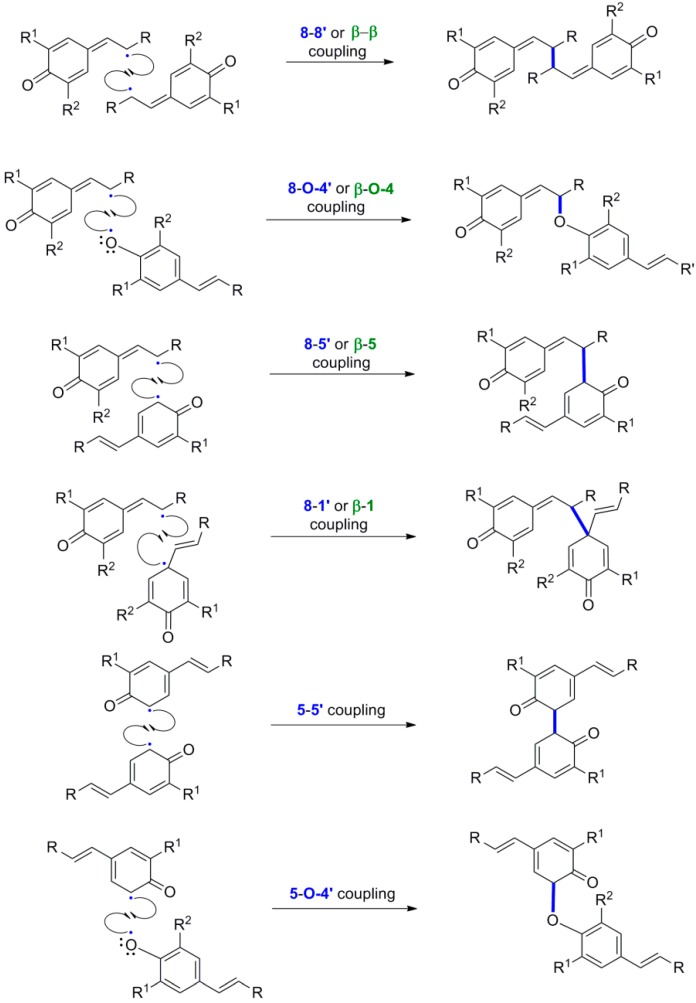
Possible combinations of the phenoxide radicals from 4-hydroxypropanoids. The primary bond formed in POC is shown in blue and bold.

Pairing of two such radicals creates the new C-C or C-O bond connecting the two monomers. There are several combinations of such radicals leading to different regioisomeric dimers (Scheme 4).

According to the 2000 IUPAC recommendations, lignans are the dimers in which the new primary C-C bond is formed between the C-8 (or C-β) of one and the C-8' (or C-β) of the other monomer [14]. All other lignans in which the monomers are connected with a bond other than the 8-8' (or β-β) bond are collectively coined neolignans (NLs). NLs in which the two monomers are connected through a primary C-O bond are particularly called oxyneolignans. The ratio of the various possible regioisomers actually formed in a particular POC depends on the stereoelectronic effects operating in the phenoxide radical, the oxidant or oxidant system and the reaction conditions. The most abundant structures in lignans are constructed by primary 8-8', 8-5' and 8-*Ο*-4' bonds. Coupling at position 5 is only possible when this position is unoccupied. On the other hand, coupling between O atoms or between C atoms both in position 1 (1-1' coupling) have not been observed in lignans because in the former case it would create a highly unstable peroxy dimer, whereas in the latter case it could not be effected due to steric hindrance because both monomers bear a propanoid side chain in position 1 [15].

Many lignans are produced in Nature in optically pure form, while others are produced as mixtures of enantiomers. Even different plants may produce the same lignan but with different enantiomeric composition [16]. The multitude of the pharmacological properties of lignans has been recently summarized [17], and include antioxidant, anti-inflammatory, antitumor and anti-HIV activities to name a few. Taking into consideration the many potential applications of lignans in medicine, a variety of synthetic methods have been devised which allow ease access to natural lignans and derivatives suitable for structure-activity relationship studies [18,19,20].

This review presents an overview of the application of POC on HCAs and derivatives, such esters and amides, as a key reaction in the efficient assembly of the skeleton of the most abundant lignan structures in nature and of analogs of potential medicinal interest. Particular attention is paid to POCs in which the anticipated regioisomer is being produced in significant excess to unwanted other regioisomers and oligomers. Interestingly, although a large array of oxidants, inorganic and enzymes, have been already developed for performing POC [21], a few of them have been proved particularly useful in producing regioselectively and/or stereoselectively the desired lignan isomer or an appropriate key intermediate.

## 2. Lignan Skeleton Assembly with β-β Bond Formation as the Key Step

### 2.1. Syntheses of Lignans Based on the Key Intermediate 4-cis,8-cis-Diaryl-3,7-dioxabicyclo[3.3.0]-octane-2,6-dione

**Scheme 5:**
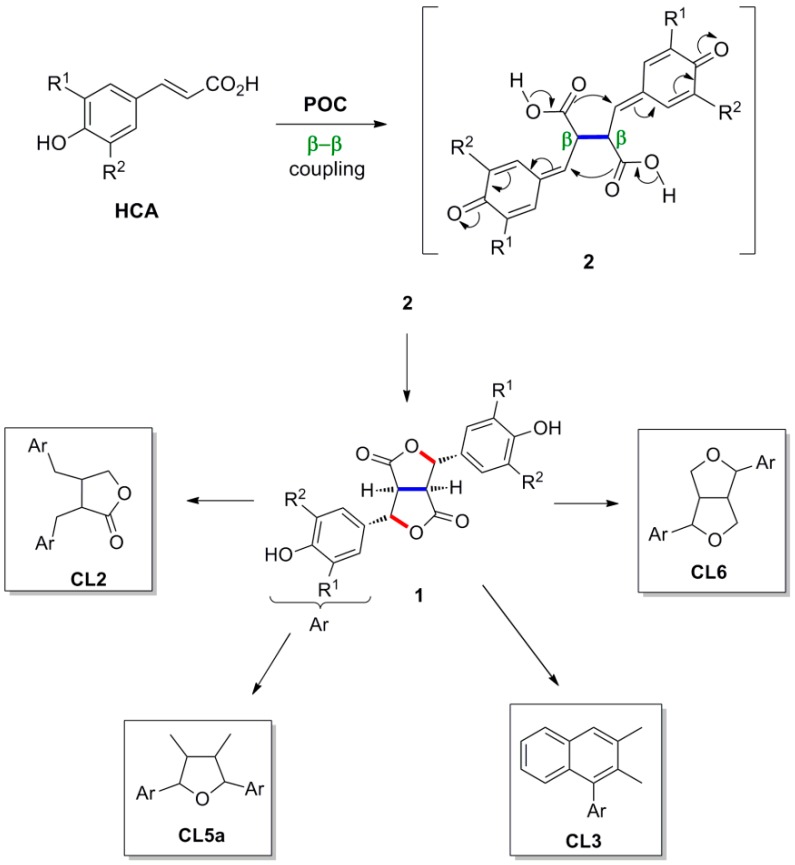
Dilactone **1** as key intermediate in the synthesis of CLs.

Dilactones of the general formula **1**, which are produced by POC of HCAs and halogenated derivatives, are key intermediates in the synthesis of several subtypes of classical lignans (CLs), such as the dibenzylbutyrolactone (CL2), arylnaphthalene (CL3), 2,5-diaryltetrahydrofuran (CL5a) and 2,6-diaryl- furofuran (CL6) subtypes. For the classification of CLs and NLs in subtypes see [20]. Dilactones **1** are formed through intramolecular nucleophilic attack of the bis-*p*-quinonemethide intermediates **2**, primarily produced through POC, by the carboxyl functions (Scheme 5).

**Scheme 6:**
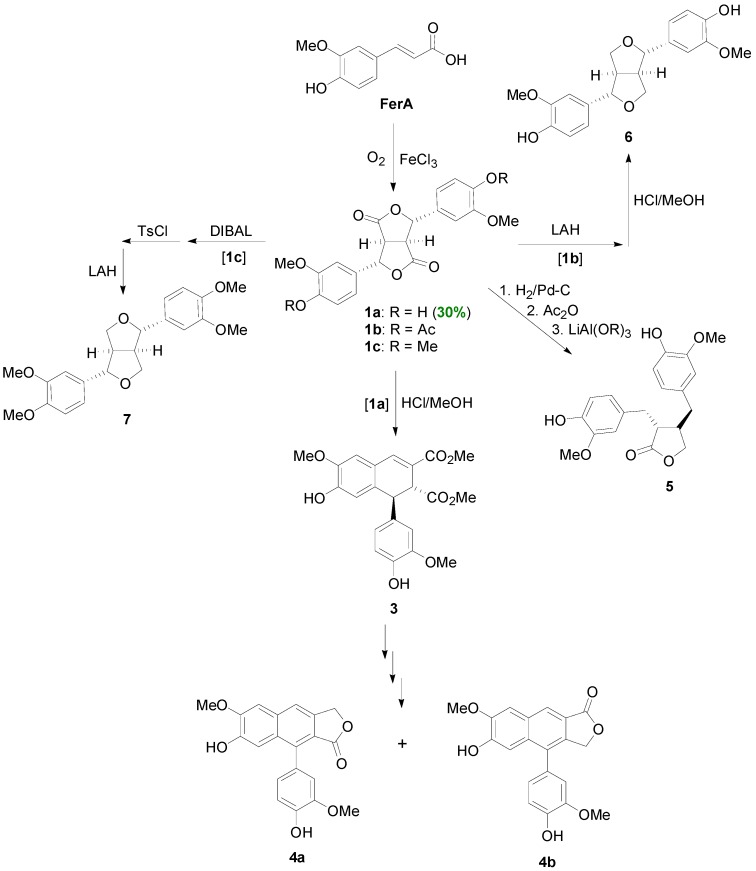
Synthesis of lignans **3**–**7** through POC of FerA and using dilactone **1a** as key intermediate.

**Scheme 7:**
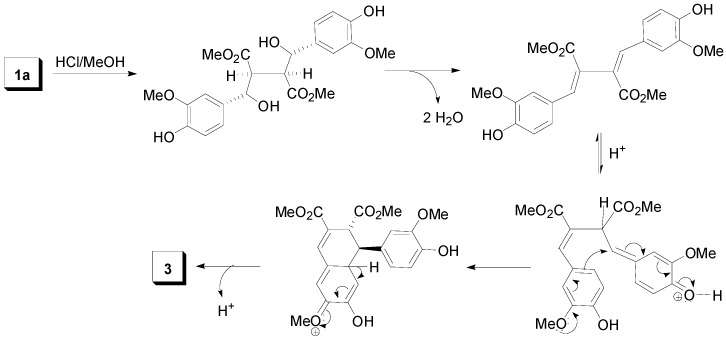
A plausible mechanism for the conversion of dilactone **1a** to arylnaphtalene **3** on treatment with HCl/MeOH.

Thus, POC of FerA mediated by FeCl_3_ and O_2_, produced dilactone **1a** in 30% yield (Scheme 6) [22,23]. Dilactone **1a**, on treatment with methanolic HCl, rearranges to aryldihydronaphthalene **3**, which in turn can be converted through several steps into a mixture of arylnaphthalene lactones **4a** and **4b** [24]. Alternatively, catalytic hydrogenation of **1a**, followed by dehydration and finally partial reduction, leads to matairesinol (**5**) [24]. On the other hand, LAH-mediated reduction of the diacetylated or dimethylated dilactones **1b** or **1c**, followed by acid-mediated cyclization of the thus obtained tetraols, provided pinoresinol (**6**) and eudesmin (**7**), respectively. Alternatively, lignans **6** and **7** may be obtained from dilactones **1b** and **1c** by partial reduction with DIBAL to the corresponding dilactols, followed by tosylation and LAH-mediated reduction [25]. The conversion of the dilactone **1a** to the arylnaphthalene derivative **3** can be envisaged to take place through transesterification, followed by double dehydration and finally electrophilic aromatic substitution on the intermediate *p*-quinone-methide (Scheme 7).

**Scheme 8:**
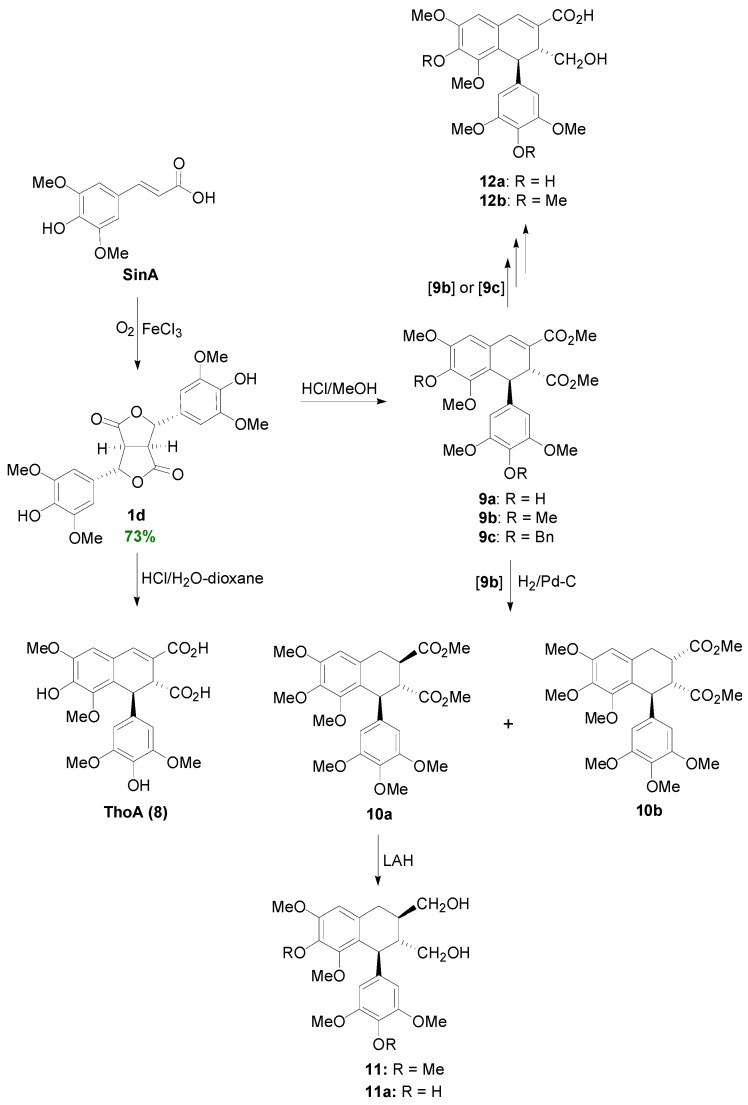
Synthesis of lignans **8–12** through POC of SinA and using dilactone **1d** as key intermediate.

On the other hand POC of SinA mediated by FeCl_3_ and O_2_, produced dilactone **1d** in 73% yield (Scheme 8) [26]. Dilactone **1d**, on treatment with aqueous acid, rearranges to ThoA (**8**), whereas with methanolic HCl, **1d** is converted to its corresponding diester **9a** [27,28]. The dimethyl ether **9b** of the latter can be hydrogenated to produce a mixture of the diastereomeric aryltetralins **10a** and **10b**, of which the former was reduced to (±)-lyoniresinol dimethyl ether (**11**) [29,30]. On the other hand **9b** or the corresponding dibenzyl ether **9c** can be transformed to either thomasic acid dimethyl ether (**12b**) or thomasic acid (**12a**), respectively, through a multi-step procedure involving LAH-mediated reduction, selective oxidation of the allylic alcohol function with MnO_2_, followed by further selective oxidation of the thus obtained aldehyde to the corresponding methyl ester with MnO_2_/HCN/MeOH, and finally either saponification or hydrogenolysis followed by saponification [27,28].

**Scheme 9:**
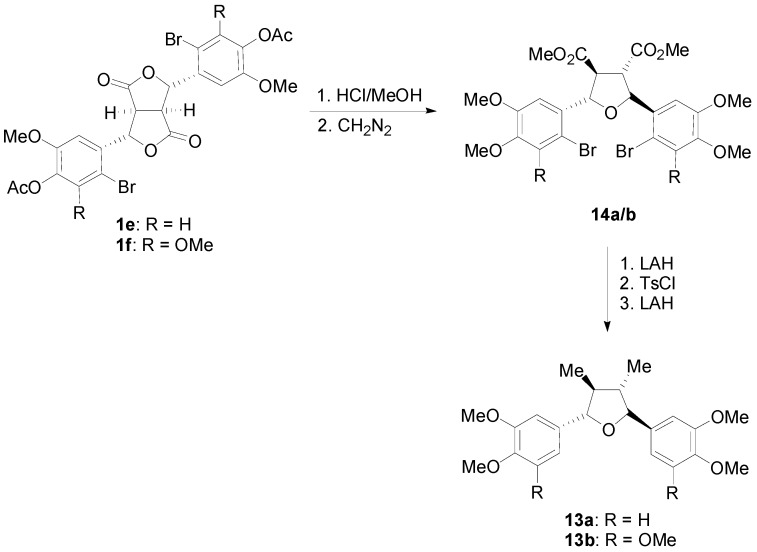
Application of halogenated lactones **1e** and **1f** to lignans of the CL5a subtype.

**Scheme 10:**
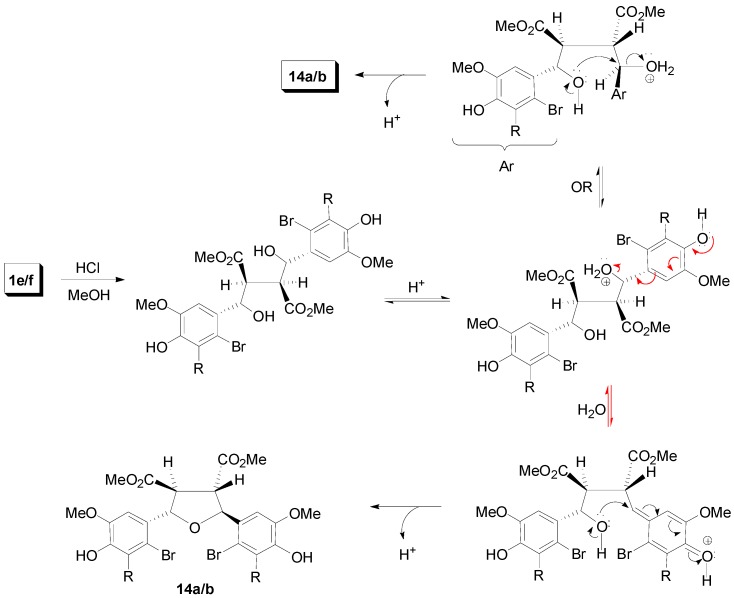
A plausible mechanism for the conversion of dilactones **1e**/**1f** to tetrahydrofuran derivatives **14a**/**b** on treatment with HCl/MeOH.

Dilactones substituted by halogens in the aromatic ring have been synthesized by either applying POC to appropriately halogenated CinAs [31,32] or by direct halogenation of acetylated dilactones [33]. Such halogen substituted dilactones like **1e** and **1f** have been used for the synthesis of lignans of the CL5a subtype, e.g., of galbelgin (**13a**) and grandisin (**13b**) (Scheme 9), through acid mediated rearrangement which however takes a different route (Scheme 10) than the one depicted in Scheme 7, precisely due to the presence of the halogen [33].

**Scheme 11:**
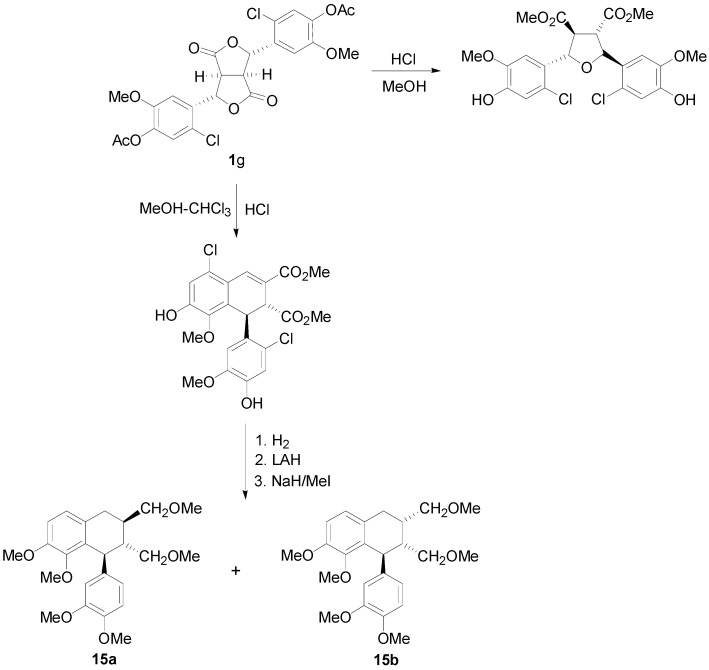
Application of halogenated lactone **1g** to the synthesis of lignans of the CL3 subtype with a different oxygenation pattern.

The presence of halogen is such dilactones may, in some other cases and depending on the reaction conditions, serve to produce aryltetralins, such as **15a** and **15b** (Scheme 11), with different oxygenation pattern than normal, by blocking the normal ring closure position [18].

**Scheme 12:**
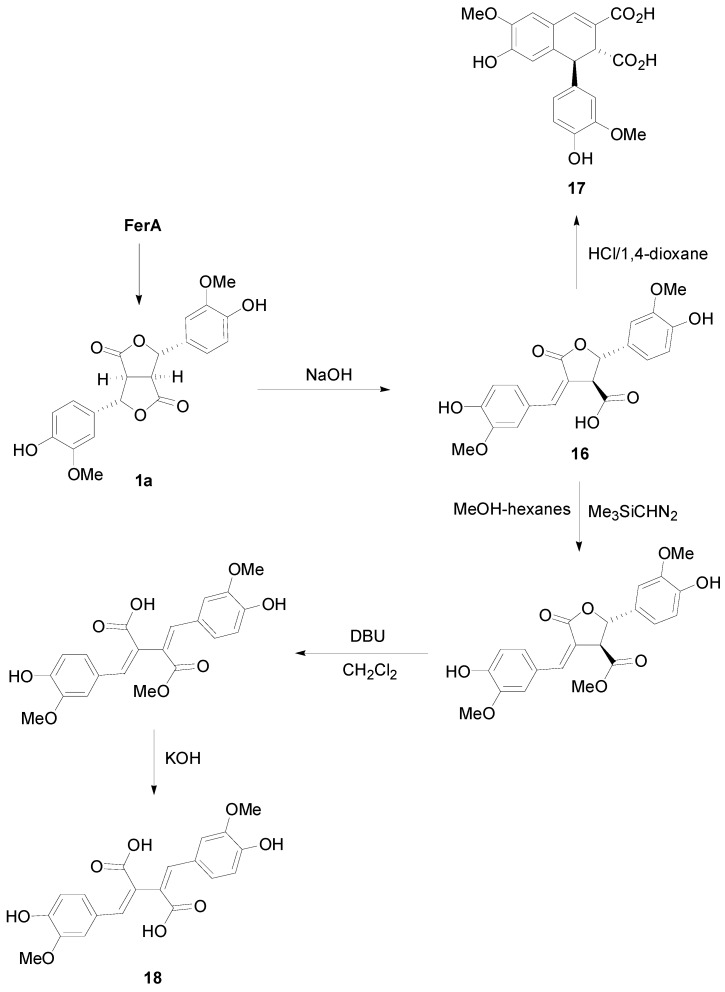
Application of dilactone **1a** from the dehydrodimerization of FerA to the synthesis of lignans of the arylnaphtalene and 2,3-bis(arylmethylene)succinic acid types.

**Scheme 13:**
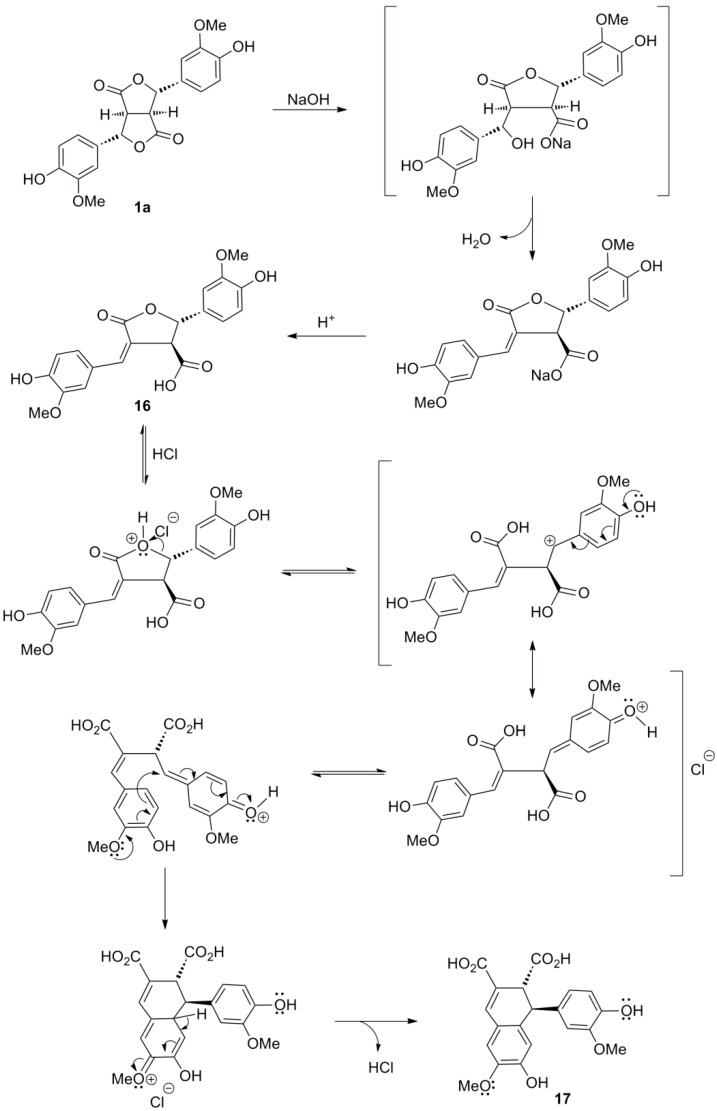
Plausible mechanisms for the conversion of the dilactone **1a** to arylnaphtalene **17** on treatment with NaOH first and then HCl.

Interestingly, dilactone **1a** have been converted to the arylnaphtalene lignan **17** and the lignan **18** of the 2,3-bis(arylmethylene)succinic acid type (Scheme 12) [34]. Thus, alkali treatment of **1a** gave as key intermediate the* trans*-γ-lactone **16** upon acidification. This compound, upon treatment with HCl rearranges (Scheme 13) to arylnaphthalene diacid **17**. On the other hand, selective methylation of the carboxyl function with diazo(trimethylsilyl)methane, followed by DBU-mediated opening (a β-elimination) of the γ-lactone and finally saponification provided lignan **18**.

**Scheme 14:**
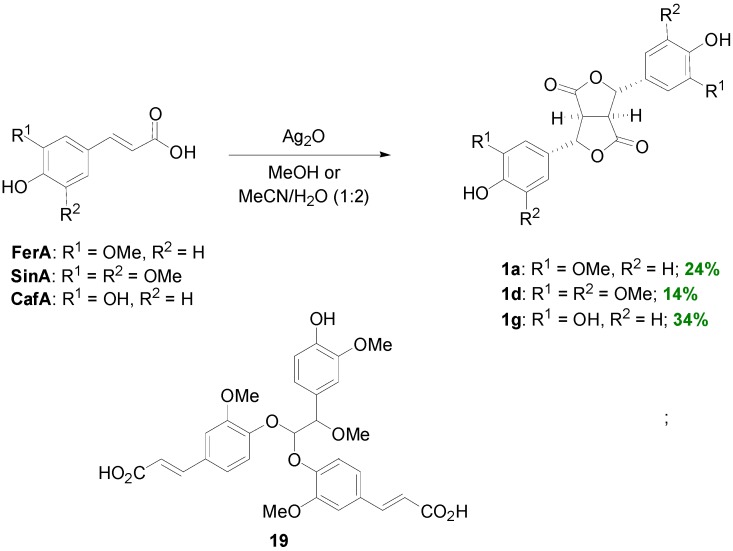
Ag_2_O-mediated dehydrodimerization of HCAs and the structure of the FerA trimer **19**.

Although the dilactones **1a** and **1d** from the dehydrodimerization of the FerA and SinA respectively, are readily available through FeCl_3_-mediated POC [23], the corresponding dilactone **1g** from the dehydrodimerization of CafA cannot be obtained using this one-electron oxidant. However, Kumada and coworkers reported that this transformation can be effected either enzymatically or non-enzymatically, using CuCl_2_ as the oxidant, in 64% yield [35]. On the other hand, Jin and coworkers demonstrated that all three dilactones may be obtained using a single oxidant, namely Ag_2_O, in yields however ranging from 14% to 34% [36]. In the case of FerA, a FerA trimer (**19**) was also isolated and characterized (Scheme 14). Evaluation of the antioxidant activity of monomers and dimers showed that the most potent were the ones bearing adjacent phenolic functionalities, that is CafA and dilactone **1g** and that dimerization results in remarkable enhancement of activity.

**Scheme 15:**
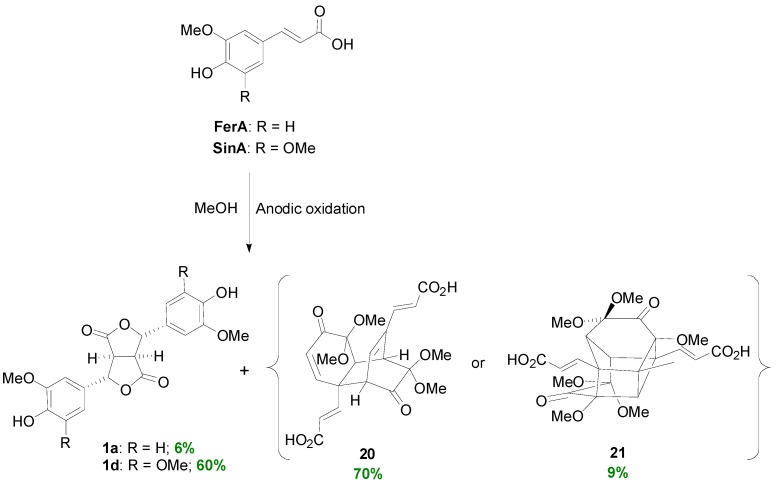
Electrochemical dehydrodimerization of HCAs.

Dilactones **1a** and **1d** have been also obtained electrochemically by the anodic oxidation of FerA and SinA, respectively, in MeOH along with NLs of the asatone (**20**)- and isoasatone (**21**)-types (Scheme 15) [37]. Interestingly, at low concentrations of HCAs only the NLs were formed.

**Scheme 16:**
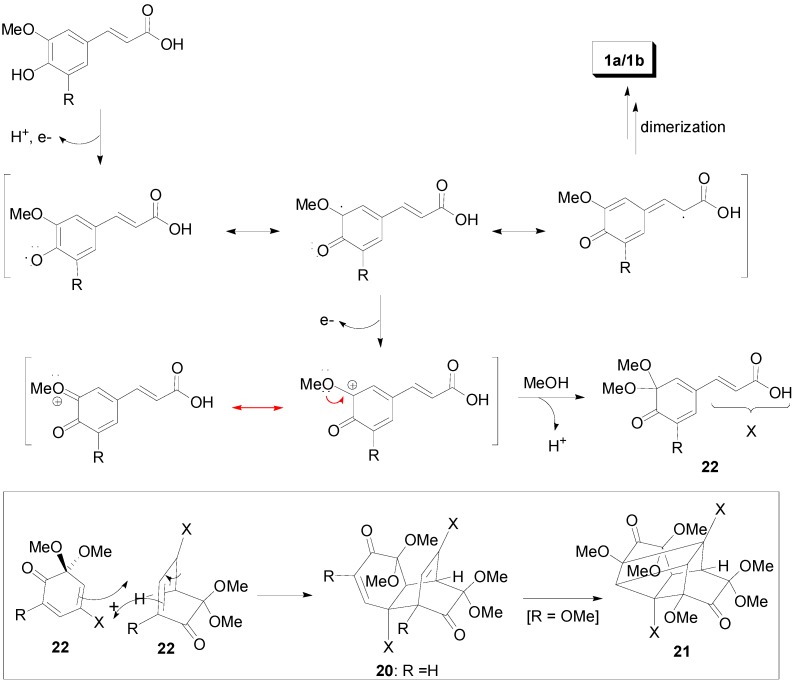
Plausible mechanisms for the electrochemical dimerization of HCAs.

These later compounds are plausibly formed through the Diels-Alder reaction of the cyclohexadienones **22**, which are obtained through two single-electron oxidations of the corresponding phenolates (Scheme 16) [37].

**Scheme 17:**
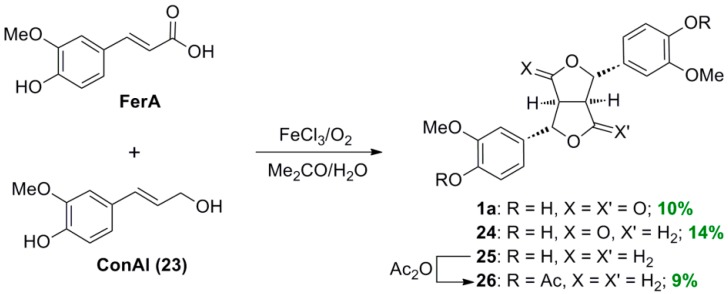
Cross POC between FerA and ConAl (**23**).

Interestingly HCAs, such as FerA, can be cross-coupled with the corresponding allylic alcohol (ConAl, **23**) to give the anticipated heterodimer, that is monolactone **24**. This compound is, however, formed in mixture with the homodimers, that is the dilactone **1a** and furofuran lignan pinoresinol (**25**) (Scheme 17) [38]. The latter was actually isolated as the corresponding diacetate **26**. Monolactone **24**, isolated from the reaction mixture through CC, is a potent germination inhibitor.

**Scheme 18:**
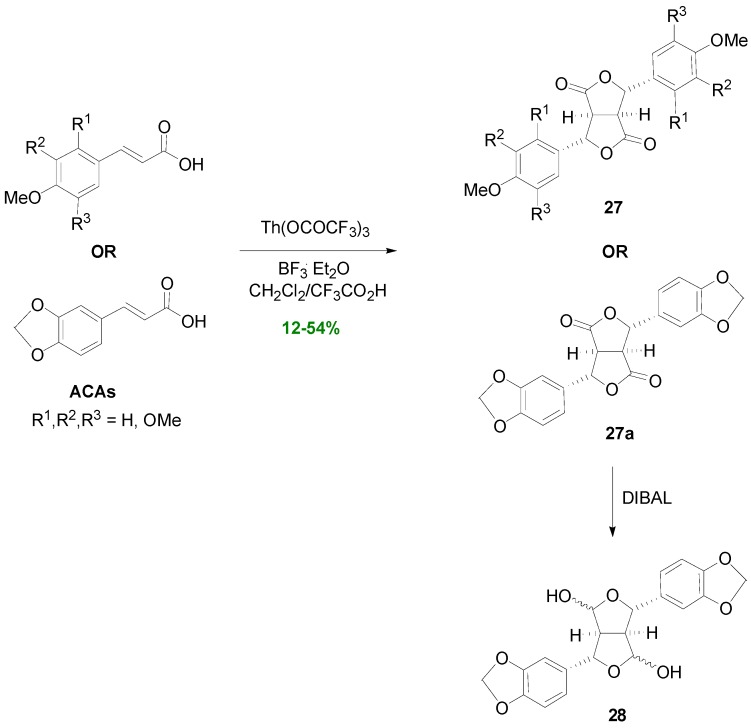
Thallium(III) trifluoroacetate-mediated dehydrodimerization of ACAs.

Dilactones of the general type **1** can be also obtained by the so-called non-POC of electronic rich aromatics [21], e.g., of ACAs. Thus, treatment of ACAs with thallium(III) trifluoroacetate in CH_2_Cl_2_/CF_3_CO_2_H in the presence of BF_3_.Et_2_O produced the corresponding dilactones **27** (Scheme 18) in yields ranging from 12% to 54%, based on recovered starting material [39]. Lactone **27a** has been partially reduced with DIBAL by Pelter and coworkers in order to prepare the lignan 4,8-dihydroxysesamin (**28**) [25]. Alternatively, CoF_3_ may be used for such dimerizations. Although the reactions are slower and less consumption of starting materials and poorer yields are observed with this reagent, its use is associated with several advantages over Th(OCOCF_3_)_3_. For example, POC may also be performed with CoF_3_ but not with Th(OCOCF_3_)_3_. Accordingly, CoF_3_-mediated dehydrodimerization of FerA produced dilactone **1a** in 20% yield.

**Scheme 19:**
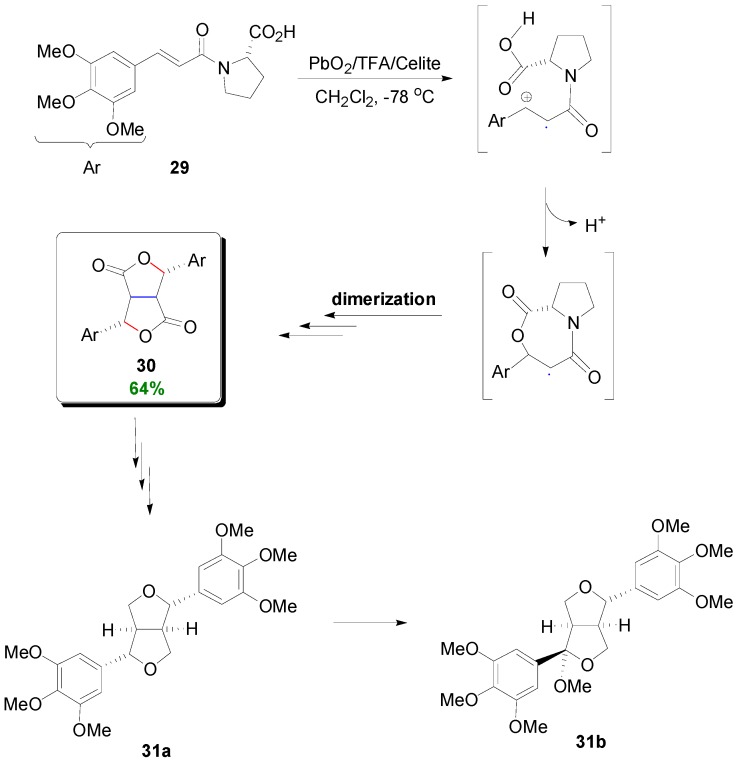
Synthesis of lignans **31a** and **31b** through oxidative dimerization of the amide (**29**) of *O*-methylsinapic acid with L-Pro.

The non-POC concept was further exploited by Mori and coworkers in the dehydrodimerization of the *N*-(3,4,5-trimethoxycinnamoyl)-l-Pro (**29**). This resulted in the stereoselective assembly of the skeleton of lignans of the 2,6-diarylfurofuran (CL6) subtype, such as yangambin (**31a**) and caruilignan A (**31b**) (Scheme 19) with a variety of pharmacological activities [40]. The method involved oxidation of the electron-rich aromatic ring of compound **29** with PbO_2_/TFA/Celite in CH_2_Cl_2_ at −78 °C to the corresponding benzylic cation radical which, through a cyclic radical, was dimerized and finally hydrolyzed to give furanofuranone **30** in 64% yield and e.e. >95% [40]. l-Pro acted as the chiral auxilliary inducing chirality in the dimerization step. Intermediate **30** was then readily transformed to the desired final products **31a** and **31b**.

### 2.2. Syntheses of Lignans Based on the Key Intermediate Dialkyl 1,2-dihydro-1-arylnaphthalene-2,3-dicarboxylate

**Scheme 20:**
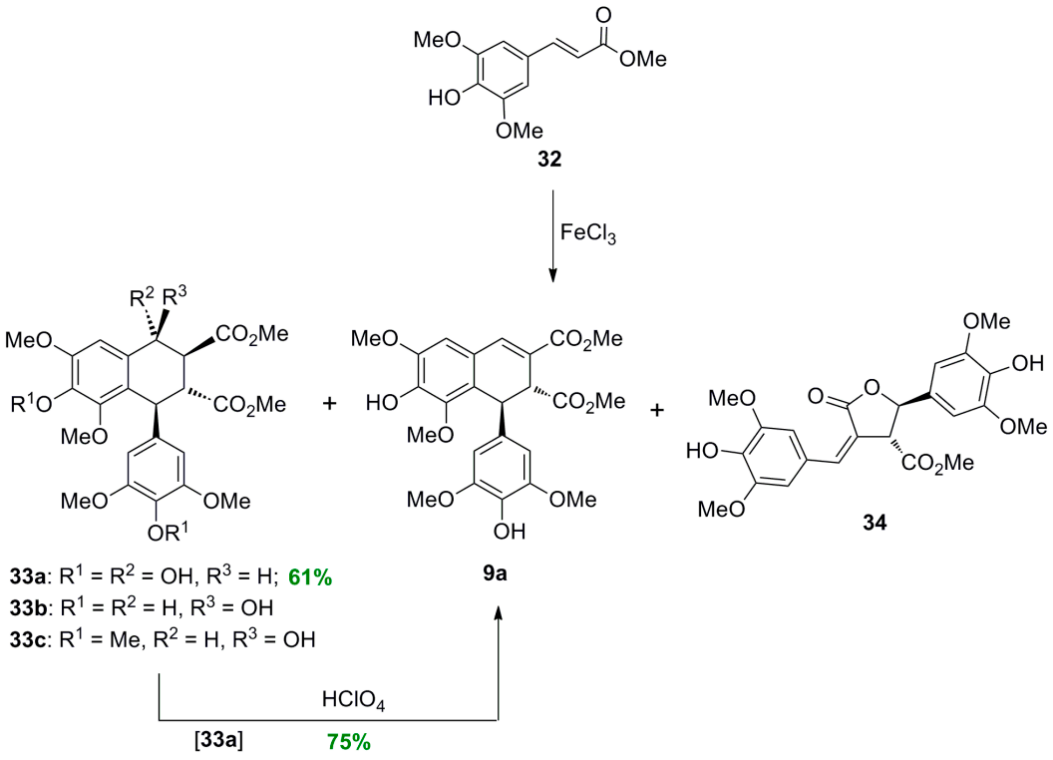
FeCl_3_-mediated POC of Me-Sin.

Treatment of Me-Sin (**32**) with FeCl_3_·6H_2_O in acetone-water produced a mixture of compounds, the major component of which was the tetralol **33a**. This compound was obtained in 61% yield following crystallization. The other components of the reaction mixture were separated with CC from the residue of the mother liquid. These included the dimethyl *trans*-1,2-dihydro-1-arylnaphthalene-2,3-dicarboxylate (dimethyl thomasidioate, **9a**), lactone **34**, and the diastereomer **33b** received in mixture with a quantity of unprecipitated **33a**. This mixture was first methylated and then rechromatographed to provide pure methyl ether **33c** (Scheme 20) [30].

**Scheme 21:**
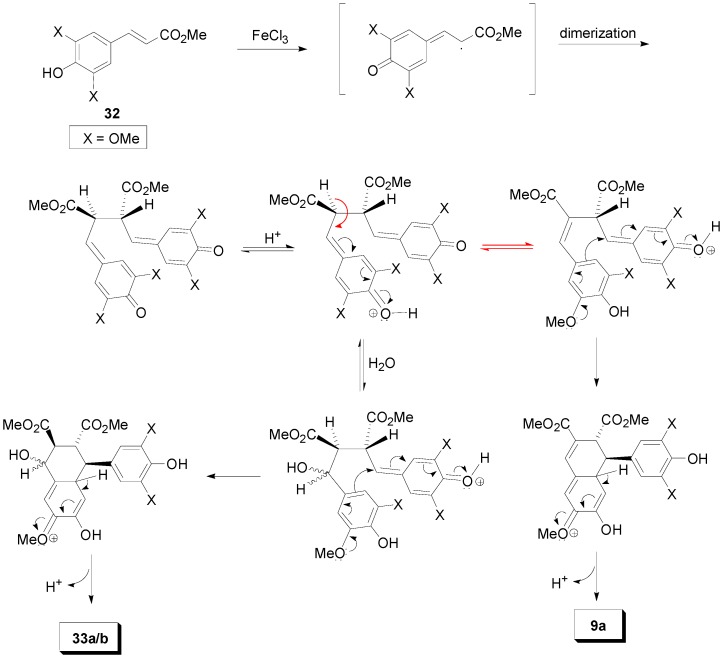
Outline of the plausible mechanisms for the formation of products **33a**,**b** and **9a** from POC of Me-Sin.

Compound **33a** was dehydrated to arylnaphthalene **9a** under treatment with perchloric acid in acetic acid. However, this conversion could not be effected under the acidic conditions (FeCl_3_, pH *ca.* 2) of the POC experiment described above. This indicates that the 4-hydroxyarylnaphthalenes **33a** and **33b** are formed through a different mechanism (Scheme 21) than the one producing the dehydrated product **9a** [29]. Interestingly, CAN-mediated POC of **32** produced a similar product mixture to that from FeCl_3_-mediated POC, whereas the use of potassium ferricyanide in acetone-water produced dimethyl thomasidioate (**9a**) in 16% yield as the only fluorescent compound under UV irradiation. On the other hand, treatment of **32** with silver carbonate produced a complex reaction mixture of non-fluorescent products.

**Scheme 22:**
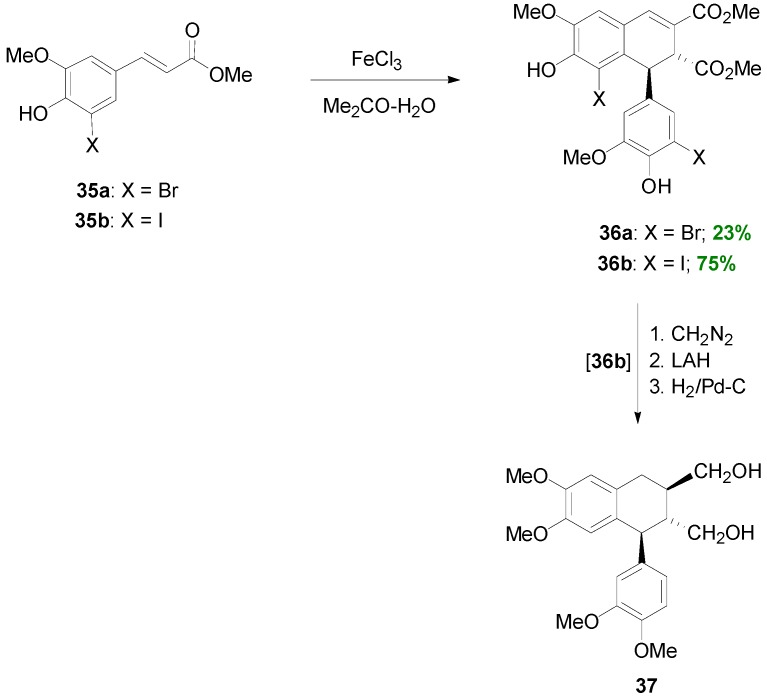
FeCl_3_-mediated POC of 5-halogenated Me-Fers.

Similarly, 5-Br or -I substituted Me-Fer (**35a** and **b**) can be directly converted to dihalogenated aryl dihydronaphthalenes (**36a** and **36b**) in 23% and 75% crude yields respectively, through POC, by using FeCl_3_ as one-electron oxidant. Dimer **36b** was further used in a synthesis of (±)-isolariciresinol dimethyl ether **37** (Scheme 22) [32].

**Scheme 23:**
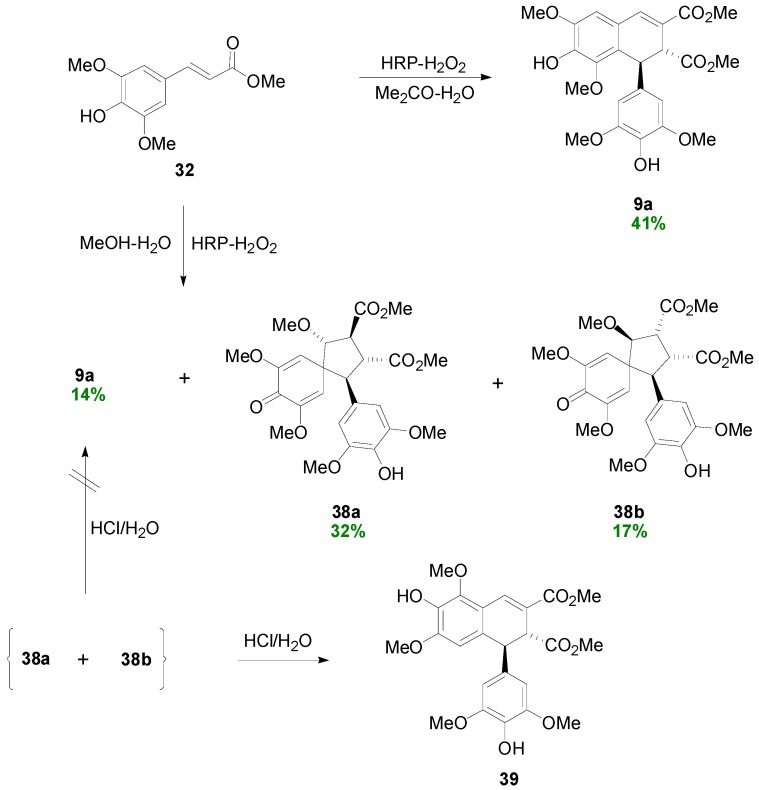
Biomimetic POC of Me-Sin with the system HRP-H_2_O_2_.

**Scheme 24:**
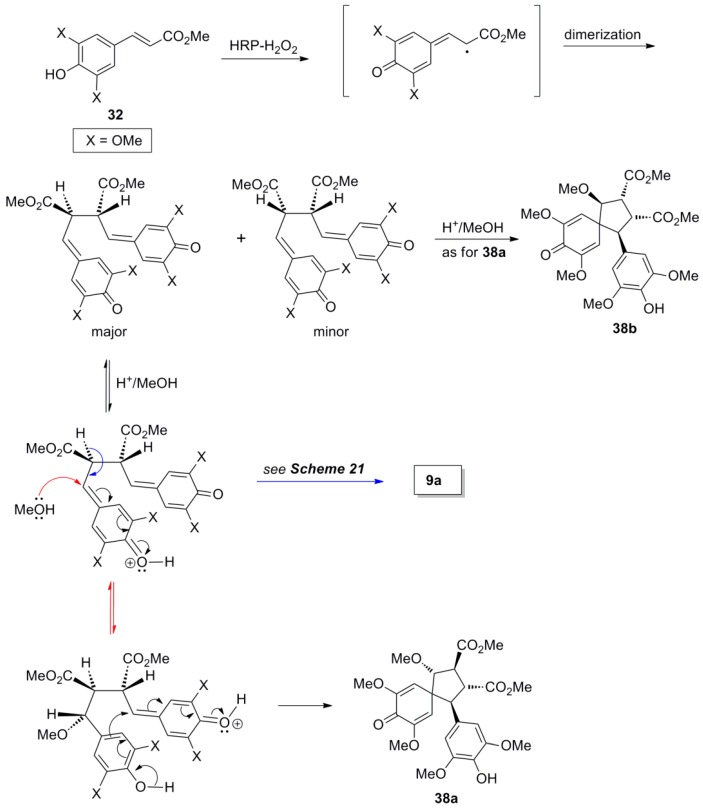
Outline of the plausible mechanisms for the formation of products **9a** and **38a**,**b**.

**Scheme 25:**
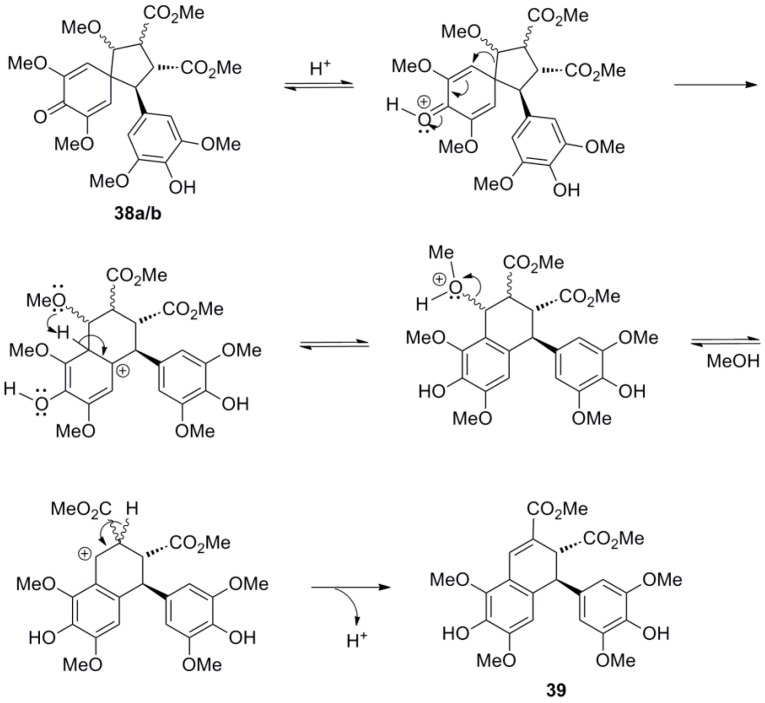
Outline of the plausible mechanism for the conversion of products **38a**,**b** to **39**.

Setälä and coworkers also obtained the aryldihydronaphthalene **9a**, in 41% yield, directly through POC of Me-Sin (**32**) but using hydrogen peroxide as oxidant and the enzyme HRP as catalyst at pH 4 in acetone-water. In earlier work, the authors had observed that in low pH-values the formation of oligomers/polymers is suppressed, thus favouring higher yields of the desired dimers. Interestingly, when the same reaction was performed in aqueous methanol dimer **9a** was produced in only 14% yield, the main product (49% yield) being a mixture of the spiro dimers **38a** and **38b** (Scheme 23 and Scheme 24) [41]. This mixture failed to produce the aryldihydronaphthalene **9a** upon attempted acid-mediated dienone-phenol rearrangement, which however led to the isomeric aryldihydronaphthalene **39** (Scheme 25). Thus, dimers **38** cannot be intermediates in the formation of **9a**.

**Scheme 26:**
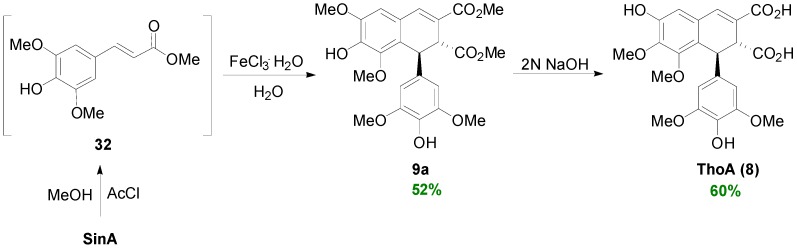
Synthesis of ThoA (**8**) from SinA.

Bunzel and coworkers synthesized dimethyl thomasidioate (**9a**) from SinA in a two-steps one-pot sequence involving treatment of SinA with acetyl chloride in methanol followed by treatment of the thus obtained* in situ* Me-Sin (**32**) with aqueous FeCl_3_^.^6H_2_O. That way, the dimer **9a** was obtained in 52% yield. Finally, saponification of diester **9a** with 2M NaOH produced ThoA (**8**) in 60% yield (Scheme 26) [42].

**Scheme 27:**
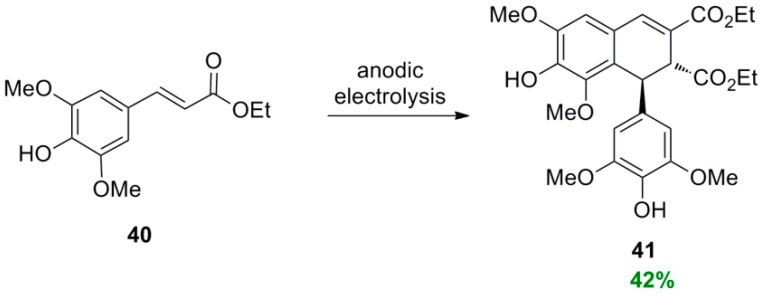
Electrochemical POC of Et-Sin (**40**).

Neudorffer and coworkers electrochemically generated the phenoxy radical from Et-Sin (**40**), in acetonitrile containing lithium perchlorate as the supporting electrolyte and a stoichiometric amount of tetramethylammonium hydroxide, which led to the synthesis of diethyl thomasidioate (**41**) in 42% yield (Scheme 27), along with 12% unreacted ester **40** [43].

**Scheme 28:**
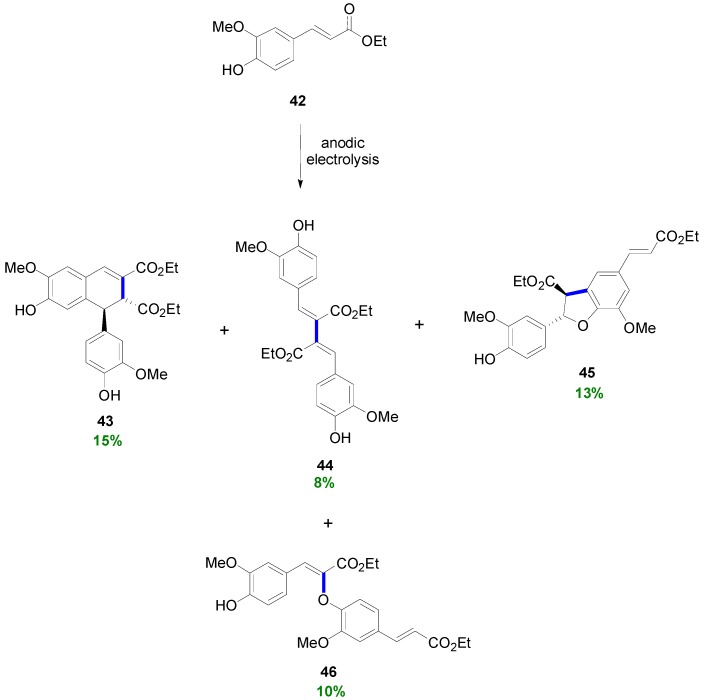
Electrochemical POC of Et-Fer. The primary bond connecting the monomers is shown in blue and bolded line.

When the corresponding Et-Fer (**42**) was subjected to anodic electrolysis under the above described reaction conditions, the corresponding dimer **43** was obtained in 15% yield along with 28% of unreacted ester **42** and three additional dimers. These included one lignan **44** of the bis(arylmethylene)succinic acid type in 8% yield, one neolignan **45** of the dihydrobenzofuran type in 13% and one oxyneolignan **46** with an β-*O*-4 bond in 10% (Scheme 28) [43].

**Scheme 29:**
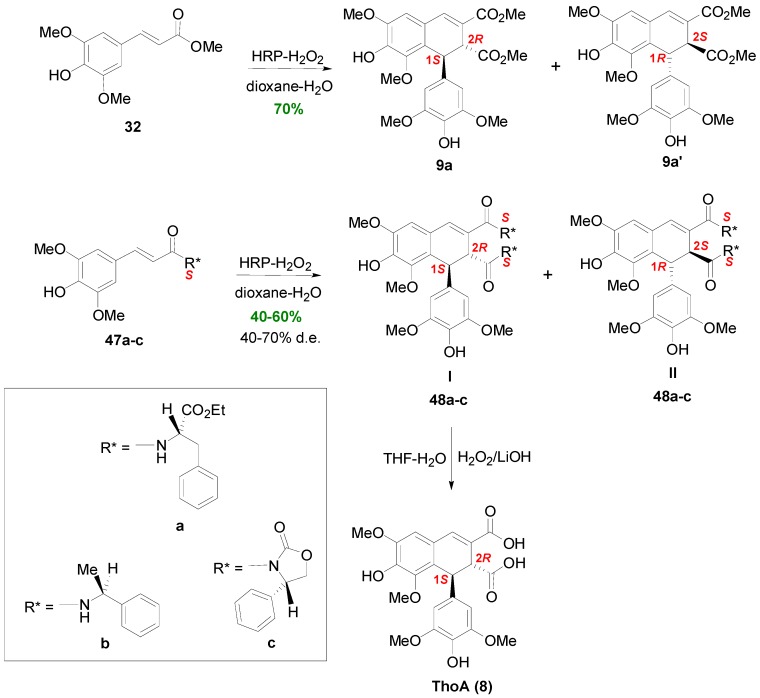
Biomimetic synthesis of ThoA (**8**) through asymmetric POC of chiral amides of SinA.

Zoia and coworkers developed an enantioselective biomimetic approach to synthesize ThoA (**8**) [44]. They subjected amides **47a**–**c** of SinA with chiral amines, such as (*S*)-Phe ethyl ester, (*S*)-methylbenzylamine and (*S*)-2-phenyloxazolidinone, the latter acting as chiral auxiliaries, to POC using hydrogen peroxide as oxidant and HRP as catalyst in a dioxane-water, buffered at pH 4, solution. The amides were obtained from the coupling of SinA and the corresponding amines in 40%–50% yields. Although POC of Me-Sin with this system produced a racemic mixture of the dimethyl *trans*-thomasidioates **9a** and **9a'** in 70% yield, the presence of the chiral auxilliaries secured the creation of mixtures of the corresponding two diastereomeric bisamides **I** and **II** (**48a**–**c**) of *trans*-ThoA with d.e. in the range of 40%–70%. The total yields (both diastereomers) of the thus obtained chiral amides of ThoA (**8**) were in the range 40%–60% (Scheme 29). Interestingly, best d.e. (70%) was obtained with (*S*)-2-phenyloxazolidinone, as the chiral auxilliary, whereas best yield (60%) with (*S*)-Phe ethyl ester, as the chiral auxilliary. Amides **I** (**48a**–**c**) could be hydrolyzed to ThoA (**8**) with LiOOH in THF-H_2_O in 10%–60% yields, the highest one obtained with the amide (**48a**–**I**) from (*S*)-2-phenyloxazolidinone. Computational methods showed that enantio- and diastereoselectivity are controlled by the β-β oxidative coupling step and the stability of the reactive conformation of the intermediate quinonemethide, respectively [44].

**Scheme 30:**
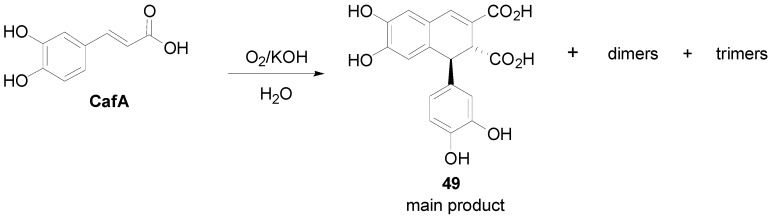
POC of CafA using O_2_ as oxidant.

Cilliers and Singleton subjected CafA to oxidation with O_2_ in an alkaline (KOH) aqueous solution (pH 8.5). Although POC on free HCAs usually leads to dilactones **1**, in this case, a mixture of mainly dimeric products (see also Section 3.1 and Section 3.3) and unreacted CafA was obtained, the main component of which was caffeicin E (**49**), that is a lignan of the substituted 1,2-dihydronaphthalene (CL3) type (Scheme 30) [45].

**Scheme 31:**
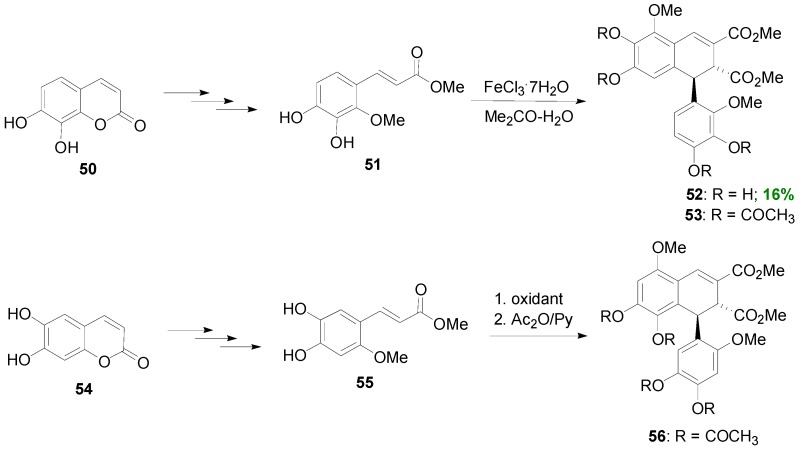
POC of HCA methyl esters **51** and **55**, obtained from coumarins.

Maeda and coworkers reported that POC of the HCA methyl ester **51**, readily derived from the coumarin daphnetin **50**, mediated by iron(III) chloride heptahydrate in aqueous acetone solution, gave 1,2-dihydronaphthalene **52** in 16% yield (Scheme 31) and 41% recovery of starting material [46]. Changing the oxidant to silver oxide or potassium hexacyanoferrate(III) in the presence of Na_2_CO_3_, resulted in very low yields (1%–5%) of **52**, isolated as the corresponding tetra-acetate (**53**), along with other dimeric products. Compound **52** was shown to be a potent inhibitor of lipid peroxidation. In an earlier study, the same authors obtained the isomeric 1,2-dihydronaphalene, isolated as the corresponding tetra-acetate **56**, as the main product from POC of methyl ester **55** using either silver oxide (28%), or potassium hexacyanoferrate/sodium acetate (20%) or sodium carbonate (13%), as the oxidant [47]. Ester **55** was readily obtained from the coumarin esculetin (**54**).

**Scheme 32:**
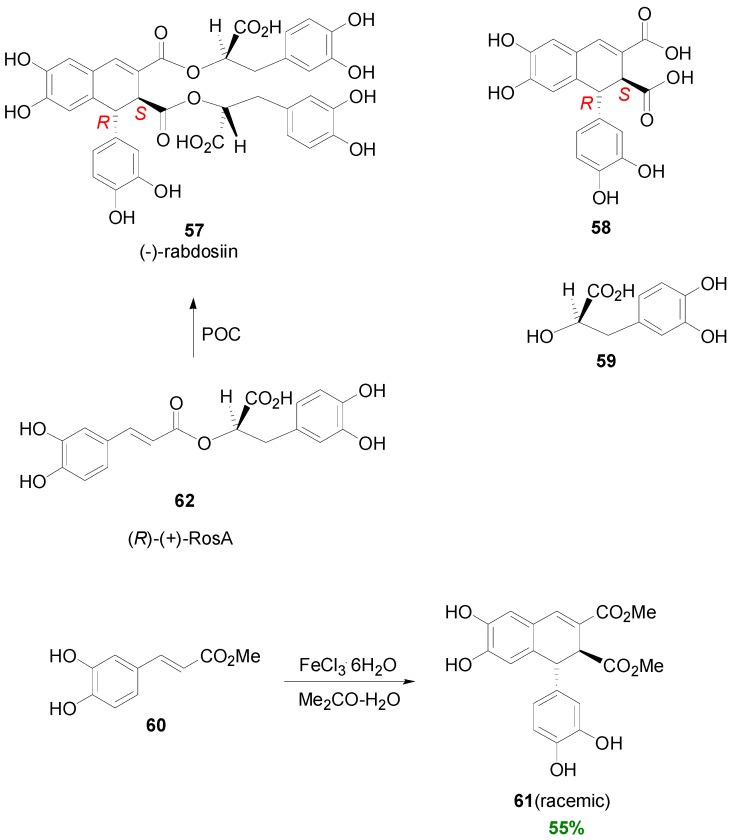
POC of Me-Caf (**60**) using FeCl_3_ as oxidant and the structures of rabdosiin (**57**) and RosA (**62**).

**Scheme 33:**
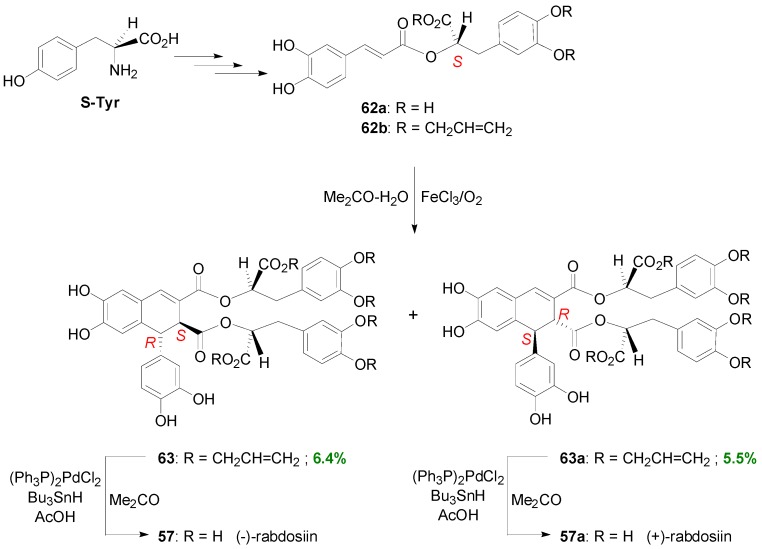
POC of a triallylated derivative **62b** of (*S*)-(−)-RosA (**62a**) *en route* to (−)- and (+)-rabdosiin (**57** and **57a**, respectively).

Agata and coworkers isolated from *Rabdosia japonica* Hara a CafA tetramer, which they named rabdosiin (**57**). The aboveground part of this plant is used in Japan as a common household medicine for gastrointestinal disorders, called “emeiso”. Compound is a diester (hybrid) of the lignan **58** of dihydronaphthalene type with the 1*R*,2*S* configuration and the (*R*)-α-hydroxyacid **59**. In order to prove its structure, this research group subjected Me-Caf (**60**) into POC using FeCl_3_ as the oxidant. That way, they obtained racemic lignan **61** in 55% yield. Rabdosiin is thought to be biogenetically produced through the oxidative coupling of two molecules of (*R*)-(+)-RosA (**62**) in a way analogous to the dehydrodimerization of Me-Caf towards the lignan skeleton **61** (Scheme 32) [48,49]. (−)-Rabdosiin and particularly its monopotassium and monosodium salts have potent anti-HIV activity [50]. It has been also found to be a strong inhibitor of DNA topoisomerases I and II [51]. Interestingly, natural rabdosiin has been isolated in both diastereomeric forms, namely the (−)-(1*R*,2*S*) and the (+)-(1*S*,2*R*) forms, although in both diastereomers the Dpl ester groups are *R*.

(*R*)-(+)-RosA (**62**) is a major constituent of the Chinese medicinal herb Danshen which has been used for the treatment of heart disease. This compound was first isolated from extracts of *Rosmarinus officinalis* and exhibits anti-inflammatory, antioxidative, antihistamine, antibacterial, antiviral and antihormonal effects [52]. Bogucki and Charlton developed a chemical synthesis to (*S*)-(−)-RosA (**62a**) from (*S*)-Tyr and CafA in 9% overall yield, which could be also applicable to (*R*)-Tyr [52]. In the latter case, (*R*)-(+)-RosA could be produced. Although attempts to oxidatively couple **62a** to obtain rabdosiin by the same authors were unsuccessful, a triallylated derivative **62b** of **62a** was indeed converted to (−)(**57**)- and (+)(**57a**)-rabdosiin, following deprotection, albeit in low yields with the (−)-isomer predominating (Scheme 33).

**Scheme 34:**
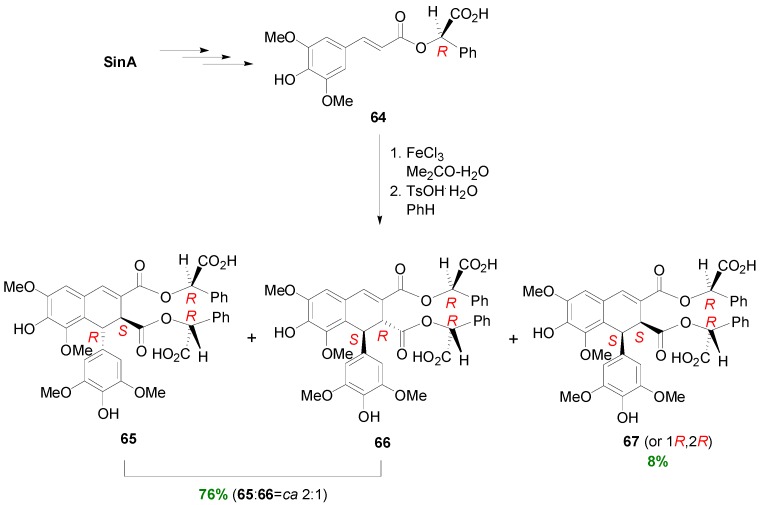
POC of ester **64** utilizing Wallis' methodology.

The same research group subjected the ester **64** of SinA with methyl (*R*)-mandelate into POC, using a similar method to that used by Wallis to dehydrodimerize the corresponding Me-Sin, and obtained three main products, namely two 1,2-*trans*-thomasidioate esters **65** and **66** in 53 and 23% yields and one 1,2-*cis* diastereomer **67** in 8% yield. It is therefore evident that chiral esters of HCAs lead to diastereoselective production of coupled-cyclized dimers (Scheme 34) [52].

**Figure 1:**
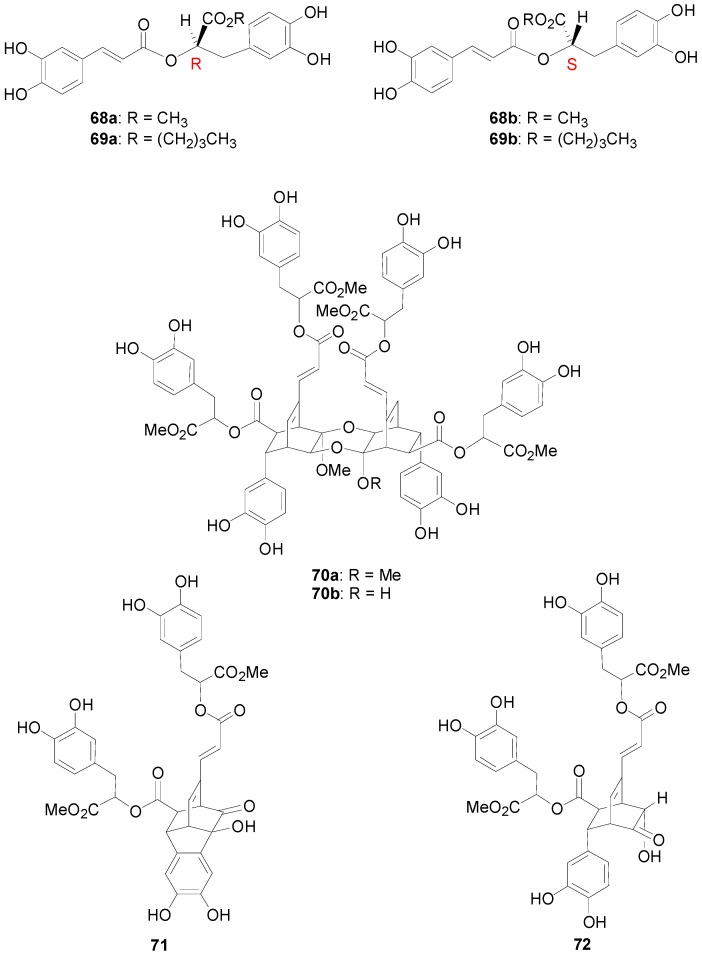
Structures of biologically interesting esters and oligomers of RosA.

It should be noted that syntheses of the methyl and butyl esters **68** and **69** of both enantiomers (*R*)-(+)(**62**)- and (S)-(−)(**62a**)-RosA have been published by Reimann and Pflug [53] and Huang and coworkers [54], respectively (Figure 1). Esters **69a** and **69b** showed interesting antioxidant and antitumour activity [54]. (*R*)-(+)-RosA has been recently shown to be a potent inhibitor against snake venom [55]. Interestingly, oligomerization of RosA in nature produces complex NLs isolated from the Indinesian plant *Helictora isora* possessing mild inhibitory activity against the avian myeloblastosis virus, such as helicterins A (**70a**) and B (**70b**), helisorin (**71**) and helisterculin A (**72**) (Figure 1). Syntheses for such compounds have been recently disclosed by Snyder and Kontes [56].

**Figure 2:**
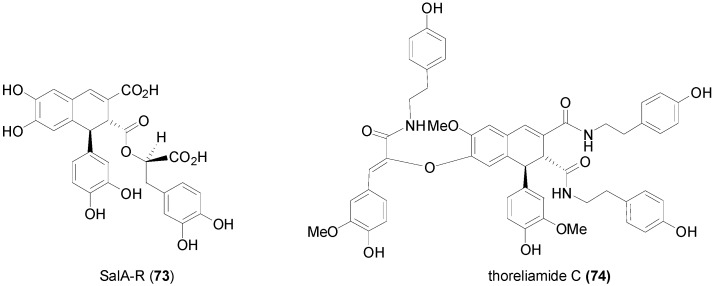
Structures of naturally occurring lignans incorporating a 1,2-dihydronaphthalene substructure.

Examples of recently reported interesting natural products of the ester (depsides) or the amide type containing the lignan dihydronaphthalene nucleus are the mono-ester **73** of caffeicin E, named SalA-R and the tris(tyramide) thoreliamide C (**74**) (Figure 2), the latter also incorporating a β-*O*-4 bond, isolated from *Origanum dictamnus* L. by Exarchou and coworkers [57] and from *Mitrephora thorelii* by Ge and coworkers [58], respectively.

**Scheme 35:**
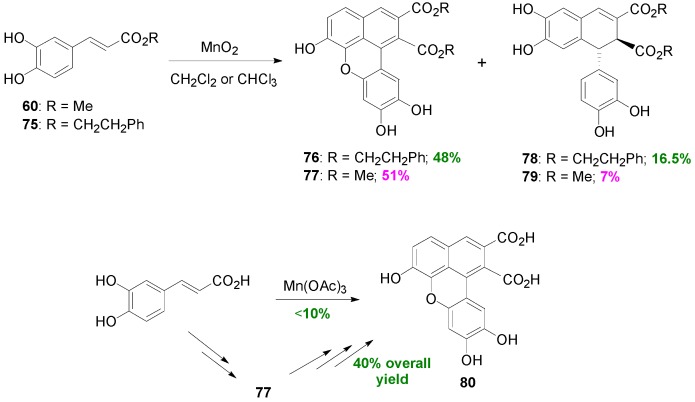
POC of CafA esters utilizing MnO_2_ or Mn(OAc)_3_ as the oxidant.

Daquino and coworkers subjected Me-Caf (**60**) and CAPE (**75**) to various oxidants and studied the products of the oxidative coupling reactions [59]. Ester **75** is a natural product contained in propolis with anti-inflammatory, antioxidant and antitumour activity [60]. Thus, POC of CAPE using MnO_2_ as oxidant in CH_2_Cl_2_ produced an unusual lignan **76** of the benzo[*kl*]xanthene type and the dihydronaphthalene **78** in 48% and 16.5% yields, respectively, along with residual CAPE (Scheme 35). Highest yield (72%) in **76** was obtained when CHCl_3_ was used as the solvent, whereas highest yield (23.7%) in **78** was obtained with *n*-hexane/CH_2_Cl_2_ (1:1) as the solvent. Under analogous reaction conditions and using CHCl_3_ as the solvent, Me-Caf gave the corresponding products **77** and **79** in 51% and 7% yields, respectively. CAPE was also oxidatively dimerized in the presence of Mn(OAc)_3_ in CHCl_3_ and produced, with 89% conversion, **76** and **78** in 71% and 22% yields, respectively. This then appears to be the best method to synthesize dimer **76**. On the contrary, CafA itself failed to produce the natural benzoxanthene lignan mongolicumin (**80**) in satisfactory yield (<10%) under these reaction conditions. However, lignan **80** could be obtained from dimer **77** through partial alkaline hydrolysis, acid-mediated cyclization and finally mild basic hydrolysis in 40% overall yield based on CafA as starting material. Benzoxanthenes have shown potential antitumour activity and, because of their strong fluorescence, may be of considerable interest in the design of new fluorescent probes for biomedical applications [59].

**Scheme 36:**
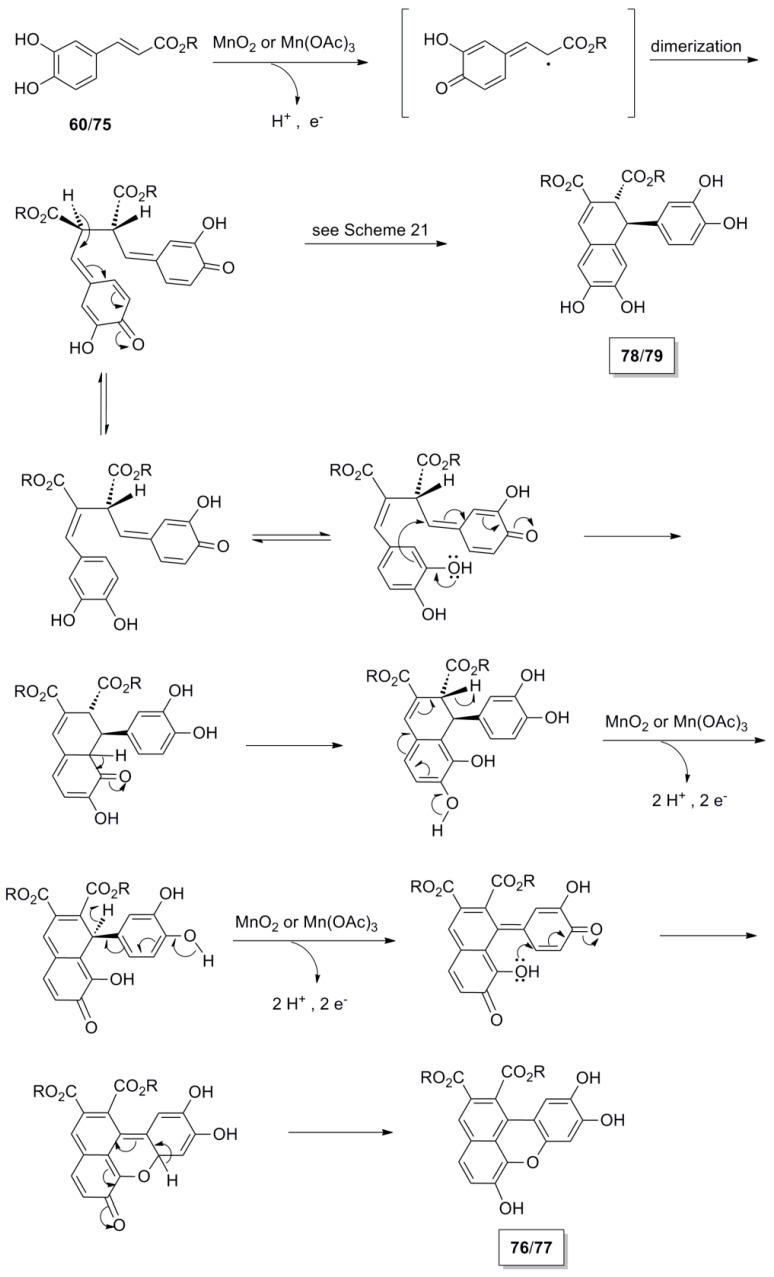
Plausible mechanism for the oxidative dimerization of CafA esters towards lignans of the benzoxanthene type.

The proposed, by the authors, mechanism for the formation of benzoxanthane lignans **76** and **77** is outlined in Scheme 36.

**Scheme 37:**
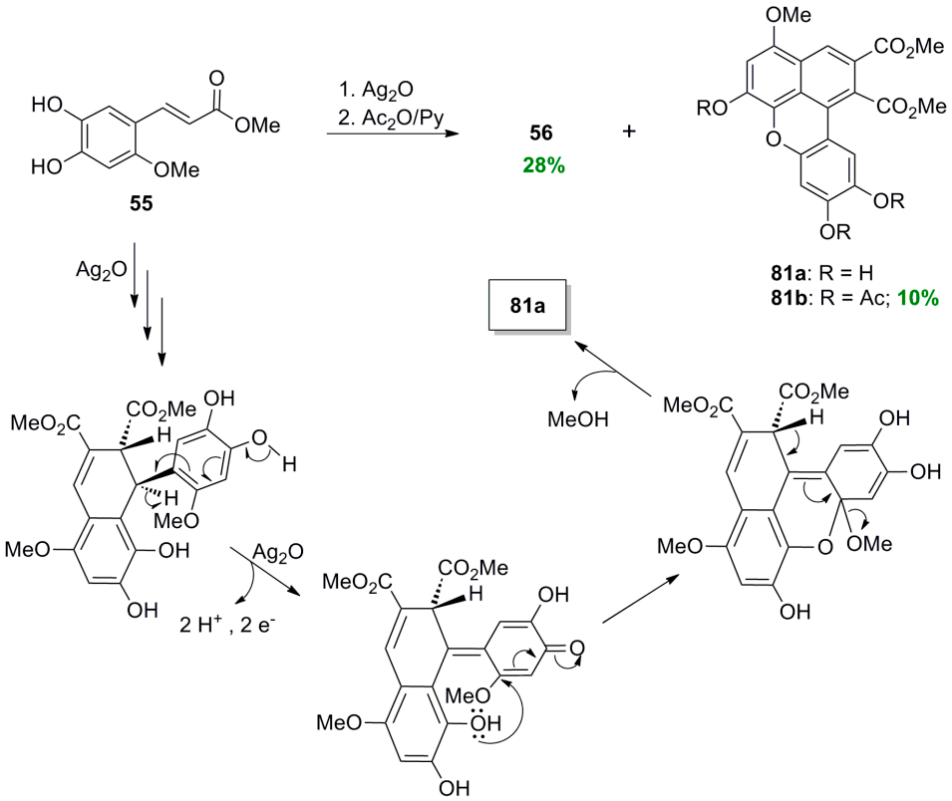
POC of ester **55** utilizing Ag_2_O as the oxidant. Stretched structures are used, in the proposed mechanism outlining formation of product, for the sake of drawing clearly electron movement and for avoiding overcrowding of groups.

It should be noted that Maeda and coworkers isolated a benzoxanthene lignan (**81a**), as the corresponding triacetate (**81b**) during the POC of the methyl ester **55** (Scheme 37), in particular when the oxidant used was silver oxide, along with the dihydronaphthalene **56** and other compounds [47]. Formation of compound **81a** may be also explained with a similar mechanism to that outlined in Scheme 36. Both compounds (**56** and **81b**) showed potent inhibition of lipid peroxidation.

**Scheme 38:**
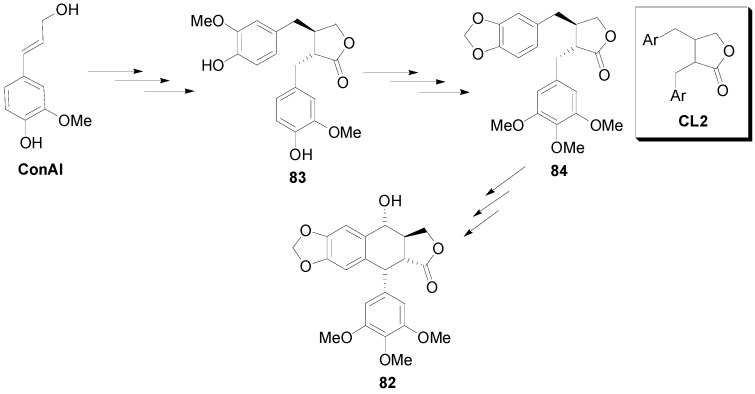
Outline of the proposed biosynthetic pathway to podophyllotoxin (**82**).

Asymmetric syntheses of aryltetralin lignans attract considerable interest due to the fact that the clinically important antitumor drugs Etoposide and Teniposide are derived from the natural lignan podophyllotoxin (**82**) (Scheme 38) [61].

**Scheme 39:**
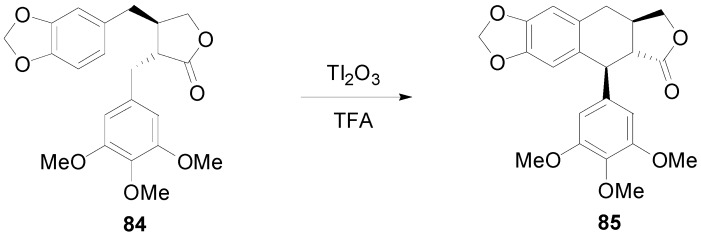
non-POC of yatein (**84**) to deoxyisopodophyllotoxin (**85**).

Taking into consideration the fact that podophyllotoxin is biosynthesized by the oxidative cyclisation of a lignan of the dibenzylbutyrolactone (CL2) type, such as matairesinol (**83**) or yatein (**84**) (Scheme 38) [62], attempts were made to effect such cyclizations using laboratory reagents. They were, however, unsuccessful as they invariably lead to the formation of an eight-membered ring rather than the anticipated six-membered ring [19]. Interestingly, Cambie and coworkers managed to cyclize yatein (**84**) by non-POC with formation of the six-membered ring, thus producing deoxyisopodophyllotoxin (**85**), using thallium(III) oxide as oxidant in TFA (Scheme 39) [63].

**Scheme 40:**
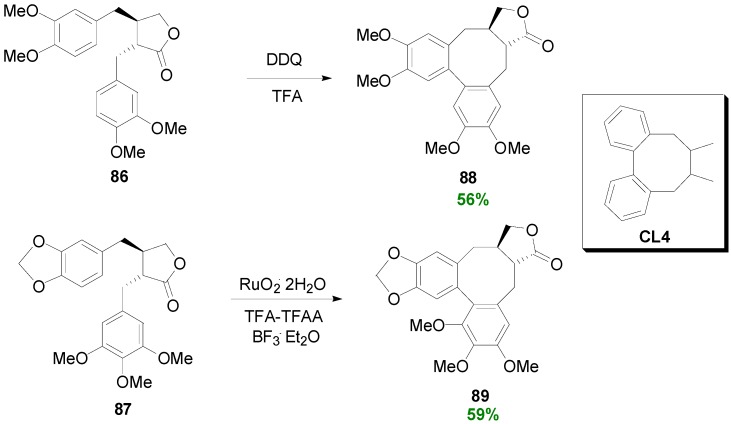
non-POC of the dibenzylbutyrolactones to isosteganes.

Non-POC on dibenzylbutyrolactones (e.g., **86** and **87**) has been also effected with other reagents, e.g. DDQ and Ru(OCOCF_3_)_4_, with formation of an eight-membered ring, thus providing access to lignans of the dibenzocyclooctadiene (CL4) type and in particular isosteganes, such as **88** and **89**, respectively (Scheme 40) [19].

**Scheme 41:**
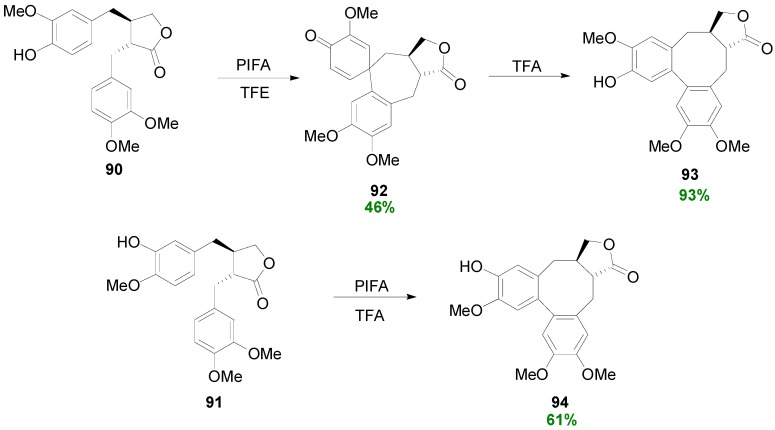
Use of PIFA in the biomimetic POC of phenolic dibenzylbutyrolactones to isosteganes.

Hypervalent iodine reagents, such as PIDA and PIFA, have been used by Ward and coworkers to effect biomimetic oxidative couplings in phenolic *trans* (e.g., **90** and **91**)- or *cis*-dibenzylbutyrolactones and which result to isosteganes (**93** and **94**, respectively) directly or indirectly through spirodienones (e.g., **92**) with acid-mediated dienone-phenol rearrangement (Scheme 41) [19].

### 2.3. Syntheses of Lignans Based on the Key Intermediate Dialkyl Bis(arylmethyle-ne)succinate

**Scheme 42:**
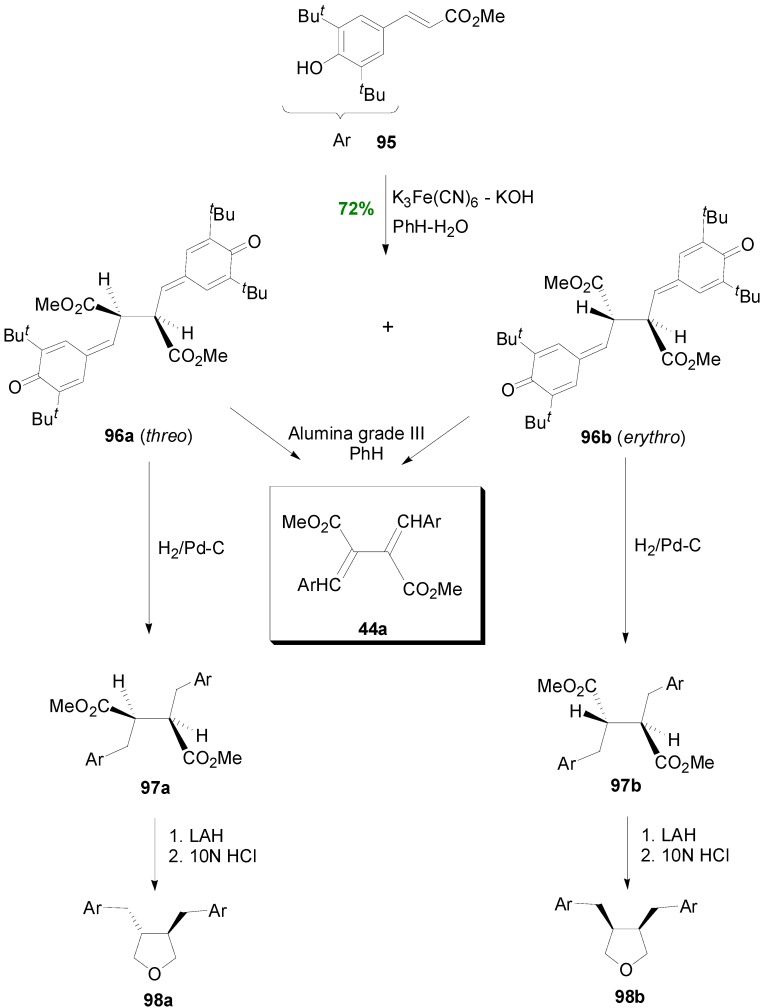
POC of cinnamate ester **95** producing bisquinonemethides **96**.

Sarkanen and Wallis subjected cinnamate ester **95** into POC coupling using alkaline potassium ferricyanide as oxidant and obtained a mixture of the bisquinonemethides **96a** and **96b**, in the ratio 65:35, in 72% yield. These compounds can be tautomerized to the same dimethyl *bis*(arylmethylene)succinate (**44a**), whereas upon catalytic hydrogenation they are transformed to the corresponding diarylbutanes **97a** and **97b**. Furthermore, diesteres **97a** and **97b** could be converted to the corresponding diastereomeric tetrahydrofurans **98a and 98b** upon reduction with LAH, followed by acid-mediated ring closure with dehydration (Scheme 42) [64].

**Scheme 43:**
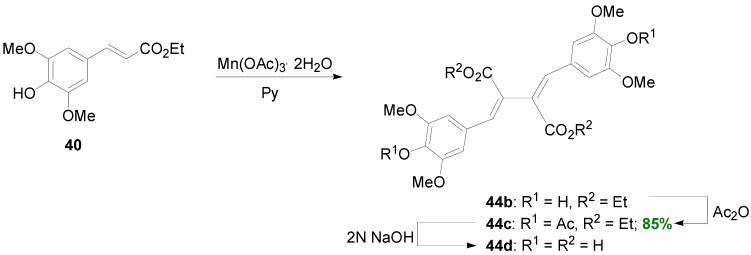
POC of Et-Sin (**40**) using Mn(OAc)_3_·2H_2_O.

It should be noted that the diethyl bis(arylmethylene)succinate **44** (Scheme 28) was identified by Neudorffer and coworkers as the minor product of the electrochemical POC of Et-Fer [43]. On the other hand, Bunzel and coworkers synthesized diethyl bis(arylmethylene)succinate **44b** through POC of Et-Sin, using Mn(OAc)_3_.2H_2_O as oxidant in pyridine (Scheme 43) [42]. They isolated its corresponding diacetate **44c** in 85% yield. From this compound, free acid **44d** was obtained, in a two-step saponification, however in low yield (30%) along with unsaponified diester **44b**.

**Scheme 44:**
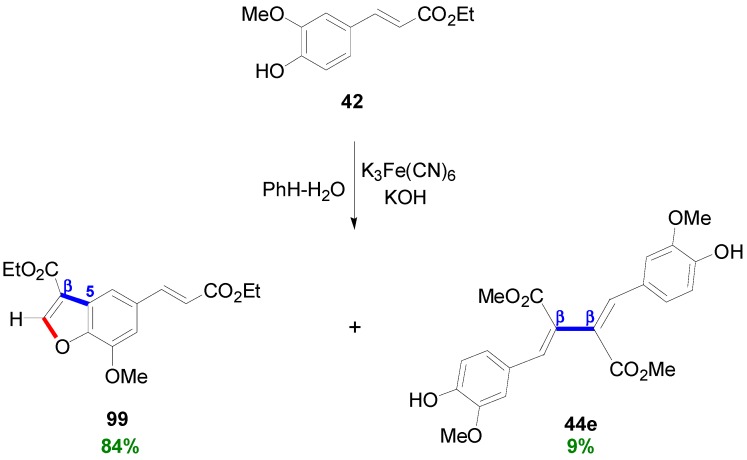
POC of Et-Fer (**42**) using K_3_Fe(CN)_6_. In blue solid line the primary bonds and in red the secondary one.

Wang and coworkers subjected Et-Fer (**42**) into an alkanine potassium ferricyanide-mediated POC in PhH-H_2_O to obtain the corresponding diethyl bis(aryl-methylene)succinate **44e**, however as a minor product of the reaction in only 9% yield (Scheme 44). The main product of the reaction was the benzofuran **99** which was obviously formed through the alternative primary β-5 coupling [65].

**Scheme 45:**
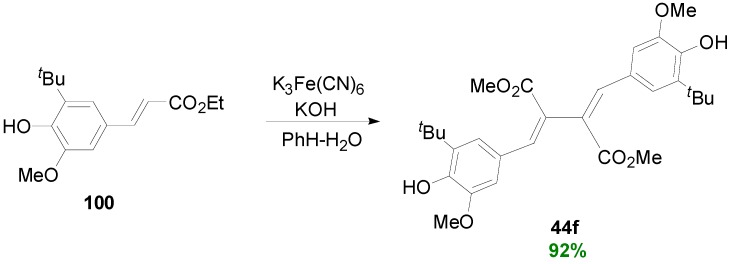
POC of ethyl 5-(*t*-butyl)ferulate (**100**) using K_3_Fe(CN)_6_.

When, however, they blocked the position 5 by a *tert*-butyl group, they succeeded to synthesize the β-β' coupling product **44f** in excellent yield from the Et-Fer derivative **100** (Scheme 45). That way, alternative dimerizations involving position C-5 or even O-4, due to steric hindrance, can be avoided.

**Scheme 46:**
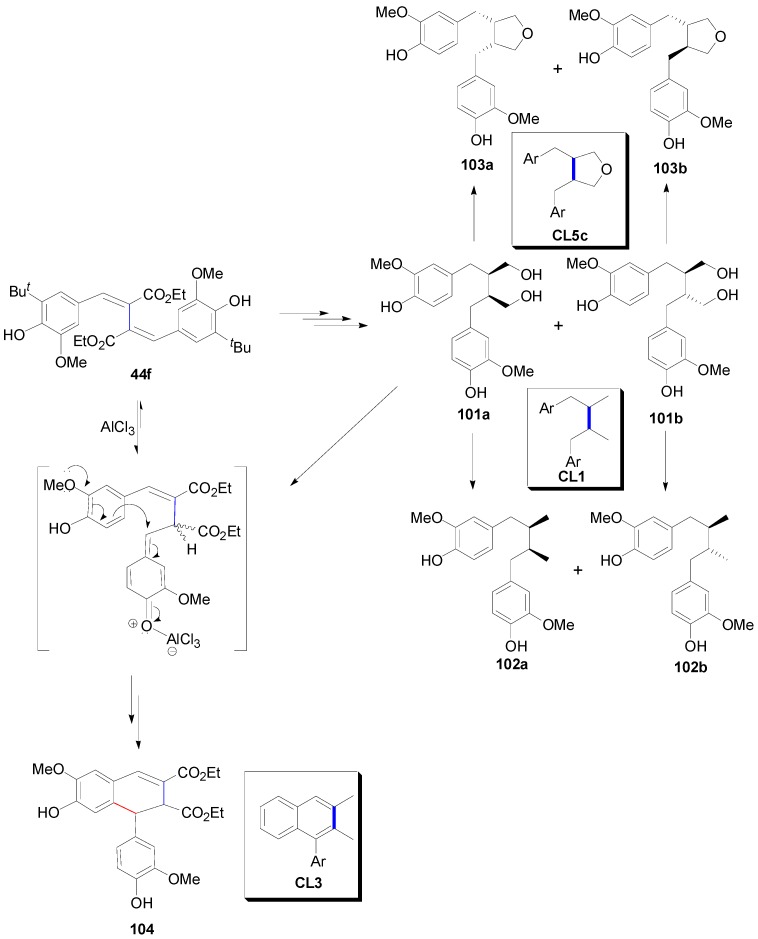
Synthesis of lignans **101**–**104** through oxidative dimerization of Et-Fer, bearing a *tert*-butyl blocking group at position 5. Stretched structures are used, in the proposed mechanism outlining formation of product, for the sake of clearly showing the electron movement.

Compound **44f** is a key intermediate in the synthesis of a variety of lignans of the dibenzylbutane (CL1), the 3,4-dibenzyltetrahydrofuran (CL5c) and the arylnaphtalene (CL3) type. Examples of lignans of these subtypes are the dibenzylbutanediols *meso*-secoisolariciresinol (**101a**) and (±)-secoisolariciresinol (**101b**), the corresponding fully reduced derivatives, the dibenzylbutanes *meso*-dihydroguaiaretic acid **102a** and (±)-dihydroguaiaretic acid **102b**, the 3,4-dibenzyltetrahydrofurans *meso*-divanillyltetrahydrofuran **103a** and (±)-divanillyltetrahydrofuran **103b** and the arylnaphalene derivative **104** (Scheme 46). The secoisolariciresinols exhibit interesting cytotoxic or immunosuppressive activities [66].

Thus, compound **44f** could be either reduced catalytically to provide access to compounds **101**–**103**, following chromatographic separation of the thus obtained diastereomeric diesters, or treated with AlCl_3_ to produce the 7,8-dihydro-7-arylnaphalene diester **104**. (Scheme 46) [65]. AlCl_3_ not only mediated the required intramolecular electrophilic aromatic substitution, obviously through a *p*-quinonemethide intermediate, but also performed the removal of the *tert*-butyl protecting group.

**Figure 3:**
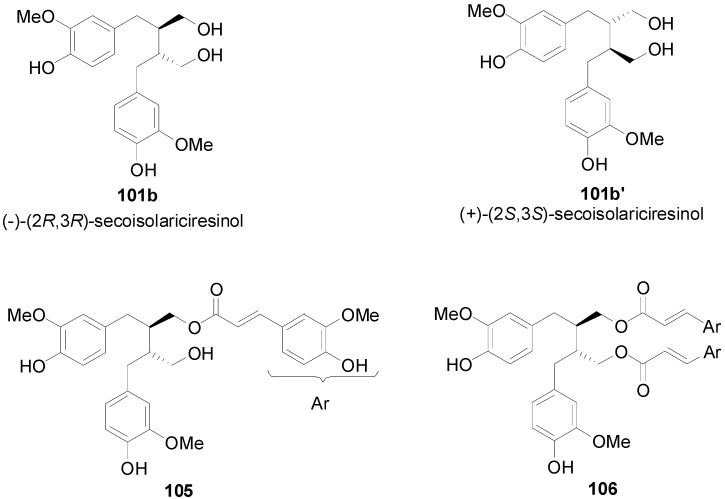
The structures of (−)- and (+)-secoisolariciresinol, hanultarin (**105**) and 1,4-*O*-diferuloyl-secoisolariciresinol (**106**).

Moon and coworkers recently isolated a novel cytotoxic lignan from the seeds of *Trichosanthes kirilowii*, identified it as the monoester of (−)-(2*R*,3*R*)-secoisolariciresinol with FerA and designated it as hanultarin (**105**). This compound and the already known diester 1,4-*O*-diferuloyl-secoisolariciresinol (**106**) (Figure 3), also isolated from the seeds, showed comparable cytotoxic effects to cisplatin against several cancer cell lines [67]. Lee and coworkers synthesized, with other than the POC route, for the first time the above compounds and analogs and evaluated their cytotoxicity against several cancer cell lines [68]. They found that, although neither FerA nor secoisolariciresinol are cytotoxic, their conjugation leads to cytotoxic hybrids with the most potent being the diferuloyl ester **106** of secolariciresinol. They also proposed that the observed cytotoxic effect might originate from the inhibitory effect of the polymerization of actin or microtubule fibers.

**Figure 4:**
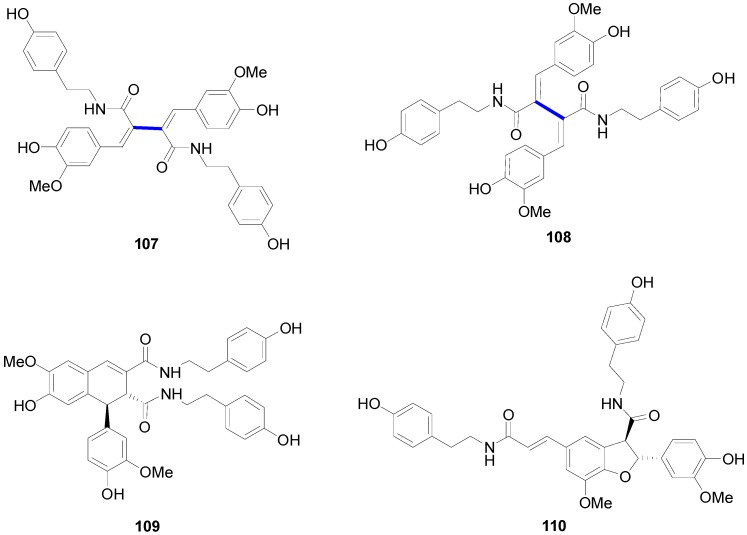
Structures of lignanamides from seeds of *Hyoscyamus niger*.

It is interesting to note that amides of the bis(arylmethylene)succinic acid have been isolated from plants. For example, Ma and coworkers isolated from the seeds of *Hyoscyamus niger* four lignanamides, that is hyoscyamide (**107**), cannabisin G (**108**), cannabisin D (**109**) and grossamide (**110**) (Figure 4) [69]. Hyoscyamide and cannabisin G are dimers of (*Z*)- and (*E*)-*N*-feruloyltyramine, respectively. On the other hand, grossamide and cannabisin D are bistyramides of dimers of FerA of the benzofuran neolignan and the arylnaphtalene lignan types, respectively. Grossamide and cannabisins D and G showed moderate cytotoxicity in cultured LNCaP human prostate cancer cells.

Tomosaka and coworkers also isolated cannabisin G (**108**), along with (±)-lyoniresinol (**11a**) (Scheme 8), from the root bark of *Berberis vulgaris* L. Although both compounds exhibited antioxidant activity in a hydroxyl radical-scavenging assay, only canabbisin G showed cytoprotective activity in cultured MCF-7 cells modulated by hydrogen peroxide [70]. Li and coworkers have recently published a concise synthesis of cannabisin G using as key reaction the alkaline ferricyanide-mediated oxidative dimerization of ethyl 5-(*t*-butyl)ferulate (**100**) [71].

### 2.4. Syntheses of Lignans Based on the Key Intermediate 3,4-Disubstituted 2,5-Di-aryltetrahydrofuran

**Scheme 47:**
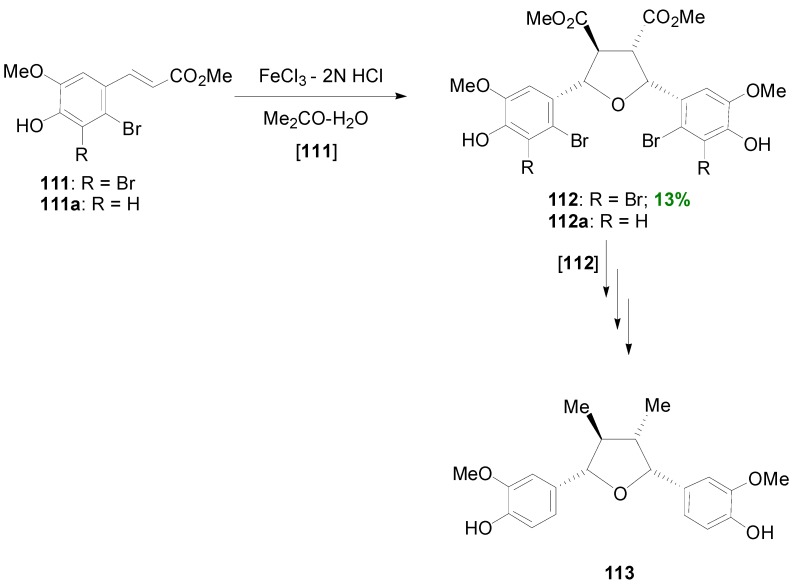
Synthesis of (±)-veraguensin (**113**).

As outlined above (Scheme 9), lignans of the 2,5-diaryltetrahydrofuran (CL5a) type can be obtained indirectly through acid-mediated rearrangement of halogenated bilactones (e.g., **1e**, **1f**). However, Ahmed and coworkers also managed to obtain directly such a compound through POC of suitable halogenated HCAs, such as **111**, using FeCl_3_ as oxidant in the presence of hydrochloric acid in low concentration. That way, they isolated the tetrahydrofuran intermediate **112** in 13% yield, along with unreacted starting material (Scheme 47). 

**Scheme 48:**
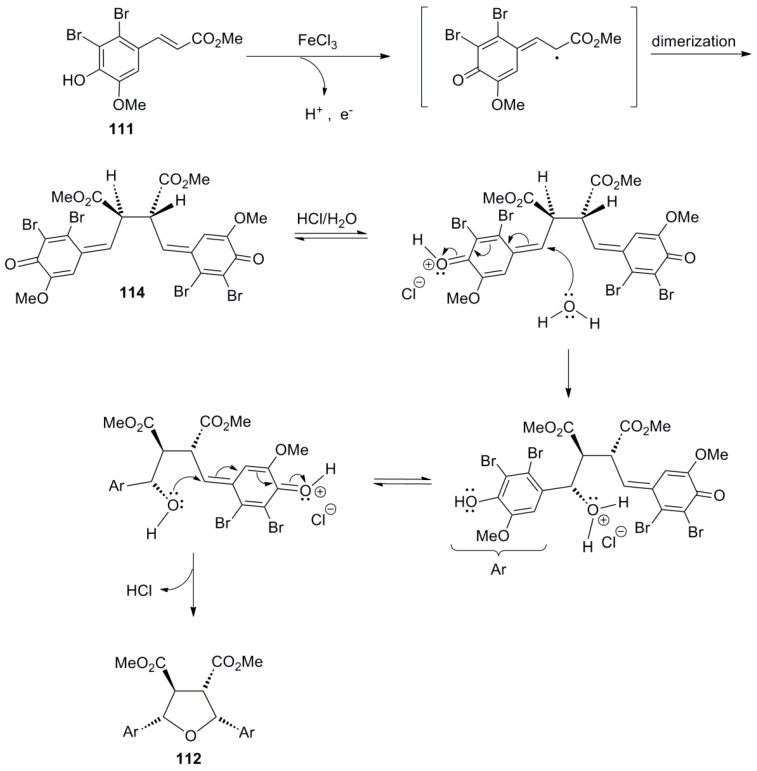
Outline of the proposed mechanism for the formation of the 2,5-diaryl- tetrahydrofuran lignan **112** by POC of HCA **111**.

This compound could be converted into the lignan (±)-veraguensin (**113**), in several steps including O-methylation, removal of the bromine atoms with catalytic hydrogenolysis, LAH-mediated reduction, tosylation and again LAH-mediated reduction [32]. However, attempts to convert directly the mono-brominated HCA **111a** to the corresponding dimer **112a** were unsuccessful [33]. Formation of dimer **112** was explained through HCl-catalyzed interception by water of the initially formed intermediate bisquinonemethide **114** (Scheme 48).

## 3. Neolignan Skeleton Assembly

### 3.1. Synthesis of NLs of the Benzofuran Type—Formation of β-5 Bond as the Key Step

**Figure 5:**
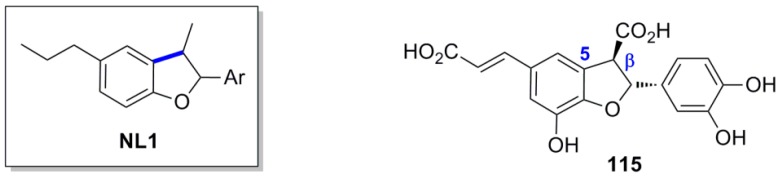
A neolignan (**115**) of the 2,3-dihydrobenzofuran type (NL1).

Cilliers and Singleton treated an alkaline aqueous solution of CafA with O_2_ and identified a series of POC products designated as caffeicins. One of them, designated caffeicin F (**115**), is a neolignan of the 2,3-dihydrobenzofuran (NL1) type [45]. Although no relative stereochemistry is provided for **115**, the coupling constant between protons 2 and 3 (*J* = 7.2 Hz) indicates a *trans* configuration (Figure 5).

**Scheme 49:**
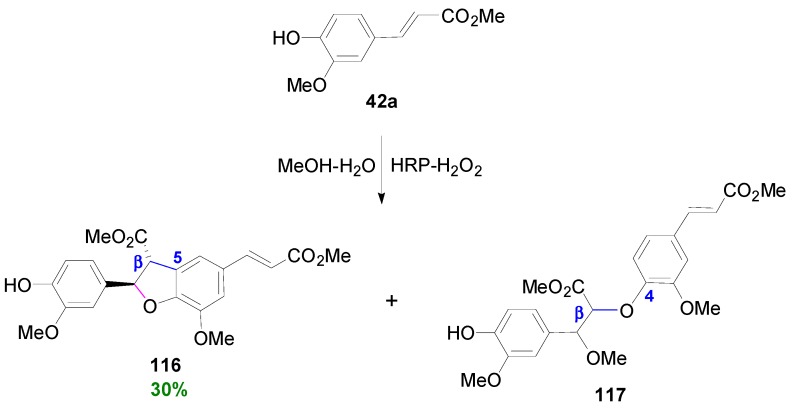
Synthesis of neolignan **116** through oxidative dimerization of Me-Fer using HRP-H_2_O_2_ as oxidant system.

Chioccara and coworkers subjected Me-Fer (**42a**) to POC using HRP-H_2_O_2_ in MeOH (10% or 90% v/v)-aqueous buffer at pH 3 to obtain the benzofuranoid dimer **116** in 30% yield after crystallization (Scheme 49) [72]. Higher pH values facilitate oligomers formation. A very minute amount of the oxyneolignan (β-*Ο*-4 dimer) **117** was also produced in the reaction in both *erythro* and *threo* diastereomers in nearly 1:1 ratio.

The same reaction could be performed using either an excess of iodosylbenzene in the presence of Mn(III)TPPOAc as catalyst or a stoichiometric amount of iodosylbenzene with MnTTPCl or even excess H_2_O_2_ with MnTPPCl to obtain benzofuran **116** in 22%, 36% and *ca.* 25% yields, respectively.

**Scheme 50:**
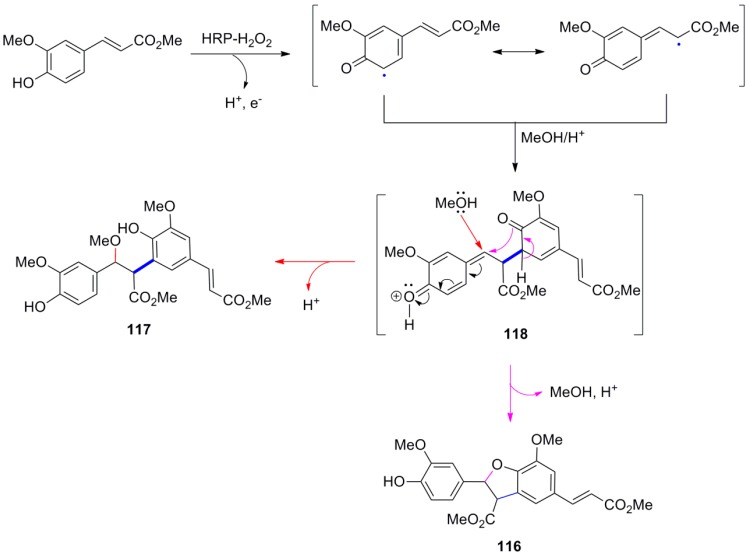
Outline of the proposed mechanism for the formation of the neolignan **116** and the oxyneolignan **117**.

Compounds **116** and **117** are thought to be formed through the common intermediate **118** which is intercepted either intramolecularly from the adjacent hydroxyl group or intermolecularly from methanol (Scheme 50).

**Scheme 51:**
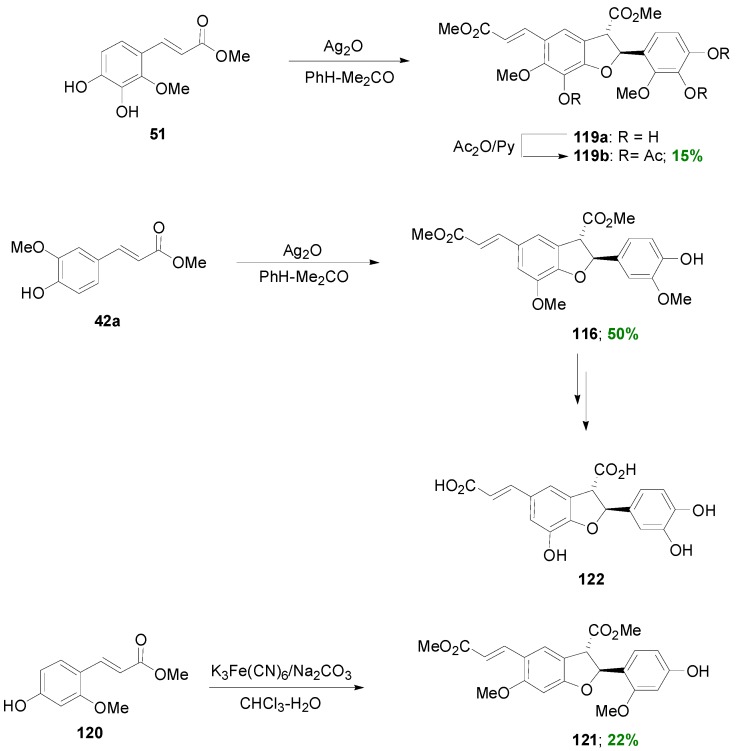
POC of Me-Fer (**42a**) and of HCA methyl esters **51** and **120**, obtained from coumarins, producing benzofuranoid NLs.

Maeda and coworkers reported that POC of the HCA methyl ester **51**, mediated by silver oxide in benzene-acetone, produced the benzofuranoid dimer **119a** as the main product, isolated as its corresponding triacetate **119b** in 15% yield (Scheme 51) [46] and 21% recovery of starting material as the corresponding diacetate. No such product was isolated by using either potassium hexacyanoferrate(III)/Na_2_CO_3_ or ferric chloride as oxidant. Compound **119b** exhibited potent inhibition of lipid peroxidation both in rat brain homogenate and rat liver microsomes. In earlier studies, the same authors synthesized the benzofuranoids **116** and **121** from Me-Fer (**42a**) and its regioisomer **120**, respectively, and converted the former into the lignin schizotenuin D (**122**) and related compounds with potent inhibitory effects on lipid peroxidation [73,74]. The common structural characteristic of all starting materials used in these POCs producing benzofuranoid NLs is that their 5 position is unhindered.

**Scheme 52:**
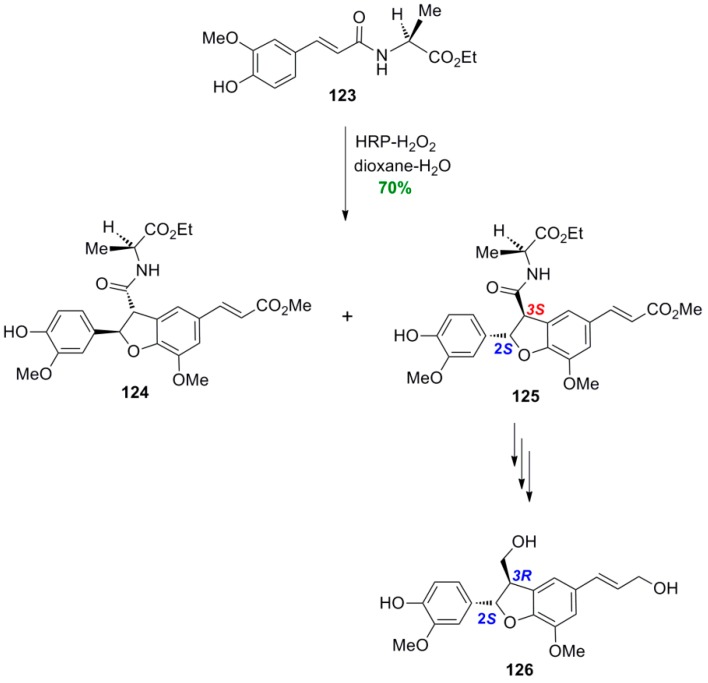
Diastereoselective POC of ferulamide **123** using HRP-H_2_O_2_.

Bolzacchini and coworkers subjected the amide **123** of FerA with ethyl (*S*)-alaninate, used as chiral auxilliary, to POC employing HRP-H_2_O_2_ as oxidant system in dioxane-aqeous buffer pH 3. That way, a mixture of the two diastereomeric benzofuranoid dimers **124** and **125** was obtained in 70% yield and d.e. 65%, which could be separated by silica gel FCC (Scheme 52) [75]. It should be reminded that, without a chiral auxilliary, the oxidant system HRP-H_2_O_2_ produces racemic mixtures. The major diastereomer had the 2*S*,3*S* absolute stereochemistry, as this was shown by LiOOH-mediated hydrolysis, esterification with diazomethane, reduction with LiBH_4_ and finally identification with the known 2*S*,3*R* stereoisomer **126** of dehydrodiconiferyl alcohol.

Ralph and coworkers developed a simple and cheap method for the preparation of diethyl diferulate **45** (Scheme 28), in gram quantities and in reasonable yields (*ca.* 50% yield), from Et-Fer (**42**) utilizing the biomimetic HRP-H_2_O_2_ system in an aqueous acetate buffer pH 4.0 [76].

**Scheme 53:**
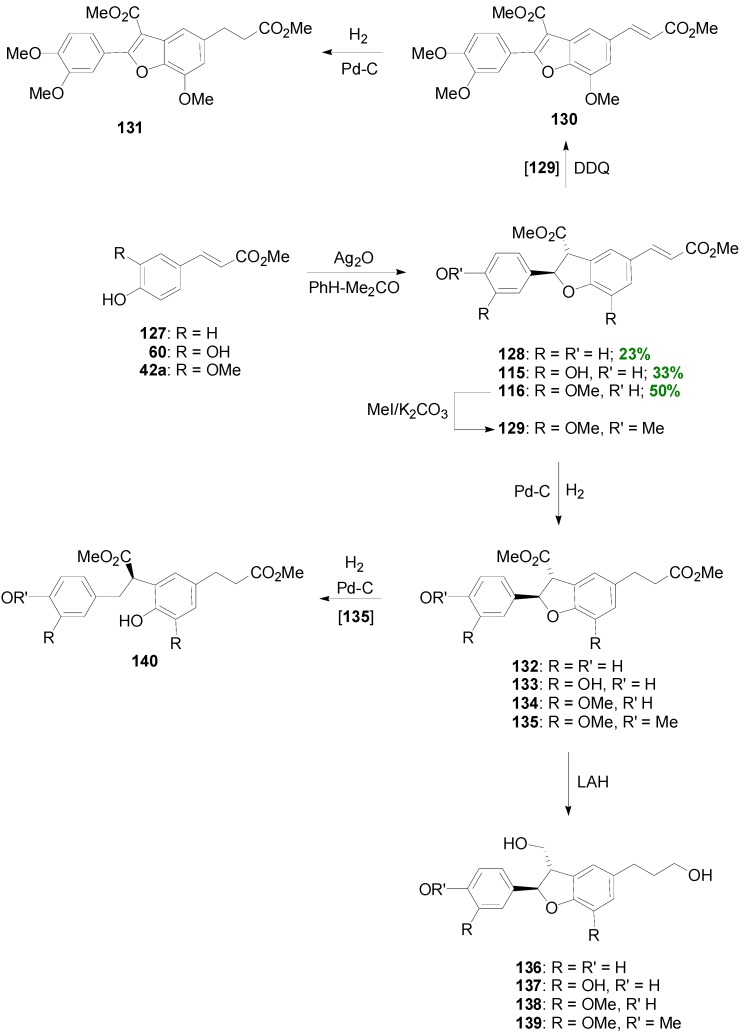
Synthesis of NLs of the benzofuran type using as key reaction the Ag_2_O-mediated POC of methyl esters of HCAs.

Considering the natural neolignan 3',4-di-*O*-methylcedrusin (**139**) with known inhibitory activity of cell proliferation as lead compound, Pieters and coworkers synthesized a series of 19 related dihydrobenzofuran lignans and benzofurans, suitable for SARS, by utilizing a biomimetic reaction sequence involving as key reaction the POC of *p*-coumaric (**127**), caffeic (**60**) or ferulic (**42a**) acid methyl esters with silver oxide in anhydrous PhH-acetone. The anticipated dihydrobenzofurans **128**, **115** and **116** were obtained in 23%–50% yields. Compound **116** was O-methylated giving the diferulate analog **129**. This compound was oxidized with DDQ to the corresponding benzofuran **130**, which was then catalytically reduced leading to benzofuran analog **131**. On the other hand, dihydrobenzofurans **115**–**116** and **128**–**129** were catalytically reduced giving analogs **132**–**135**, which were further reduced with LAH leading to the diols **136**–**139**. Furthermore, catalytic hydrogenation of **135** caused opening of the dihydrofuran ring giving alcohol **140** (Scheme 53). All synthesized compounds were tested for potential anticancer activity in 60 human tumor cell lines. The dihydrobenzofuran dimer **115** from Me-Caf was the most potent. It should be noted that the two enantiomers of racemic **115** were separated by chiral HPLC and that the 2*R*,3*R* enantiomer was the more active one. Furthermore, the two enantiomers of racemic neolignan **129** were also separated in order to obtain 3',4-di-*O*-methylcedrusin (**139**), with the 2*R*,3*S* assigned configuration, a minor constituent of the traditional medicament “dragon’s blood”. Surprisingly, no activity was exhibited by this particular compound. Leukemia cell lines and breast cancer lines were relatively more sensitive to the cytotoxic dihydrobenzofuran NLs. These studies established that the NLs of the dihydrobenzofuran type form a new group of antimitotic and potential antitumor agents which inhibit tubulin polymerization [77].

**Scheme 54:**
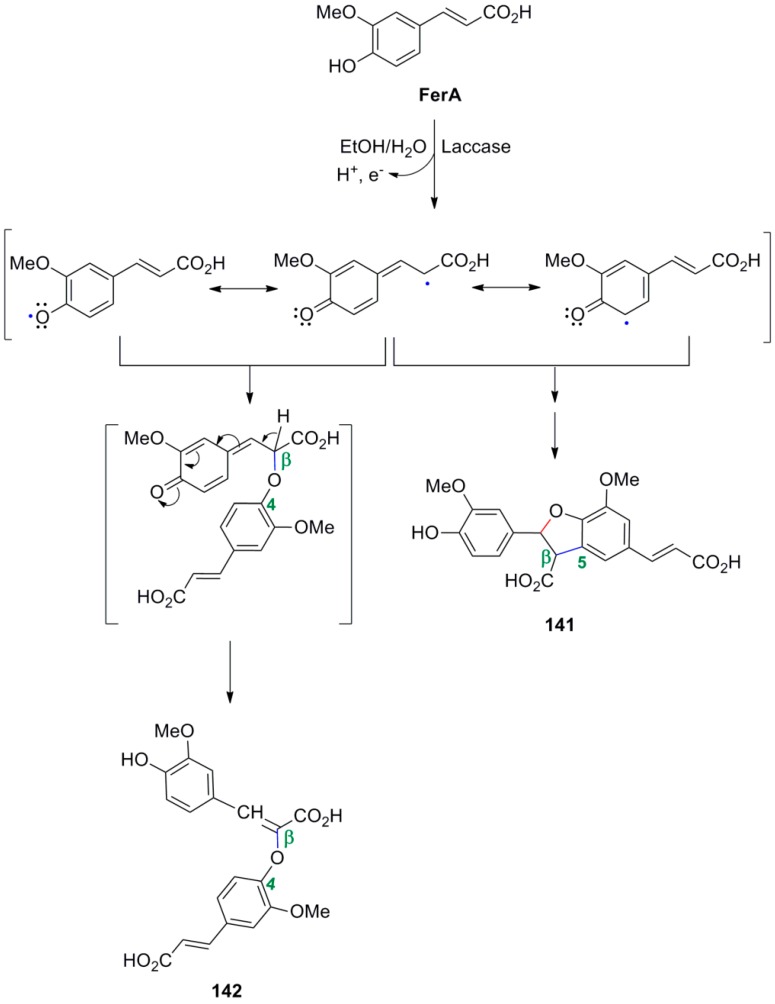
Dimers from laccase-mediated POC of FerA.

Carunchio and coworkers subjected FerA to POC using the enzyme laccase in EtOH-aqueous buffer pH 6.0 and the reaction was monitored by HPLC-MS. They identified the two main products initially formed as the benzofuranoid dimer **141** and the β-*O*-4 coupling product **142** (Scheme 54) [78]. They also found that enzymatic activity is inhibited by dipeptides of the type Gly-X due to the formation of complexes with Cu(II), known to play a central role in the laccase, single electron transfer, reaction mechanism.

A dihydrobenzofuran dimer, namely compound **45** was also isolated by Neudorffer and coworkers, as the second most abundant compound (13% yield) of the mixture obtained by the electrochemical POC of Et-Fer (Scheme 28) [43].

**Scheme 55:**
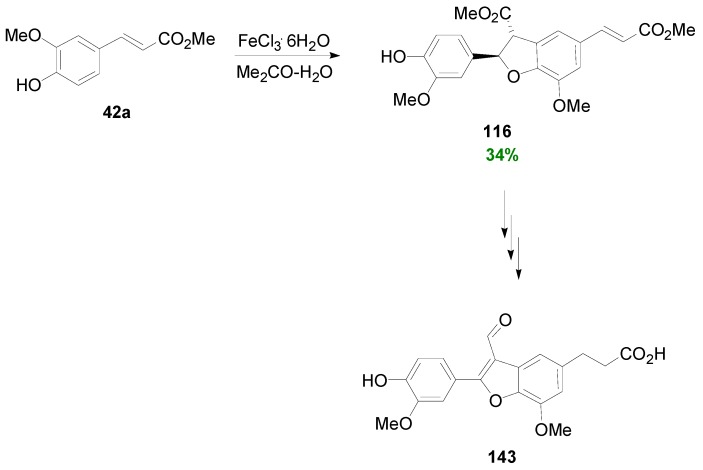
Synthesis of salvinal (**143**) through FeCl_3_-mediated POC of Me-Fer (**42a**).

Kuo and Wu reported a biomimetic multistep synthesis of salvinal (**143**) [79], a benzofuranoid NL isolated from the root of *Salvia miltiorrhizae* Bunge, whose aqueous extracts have been extensively used in Asia in the treatment of cardiovascular disorders and cancer. Salvinal is a novel Adenosine A_1_ receptor ligand and a novel microtubule inhibitor with antimitotic activity in multidrug-sensitive and -resistant human tumor cells [80]. Key step in the synthesis of salvinal was POC of Me-Fer by ferric chloride in aqueous acetone (Scheme 55) producing the known dihydrobenzofuran dimer **116** in 34% yield.

**Scheme 56:**
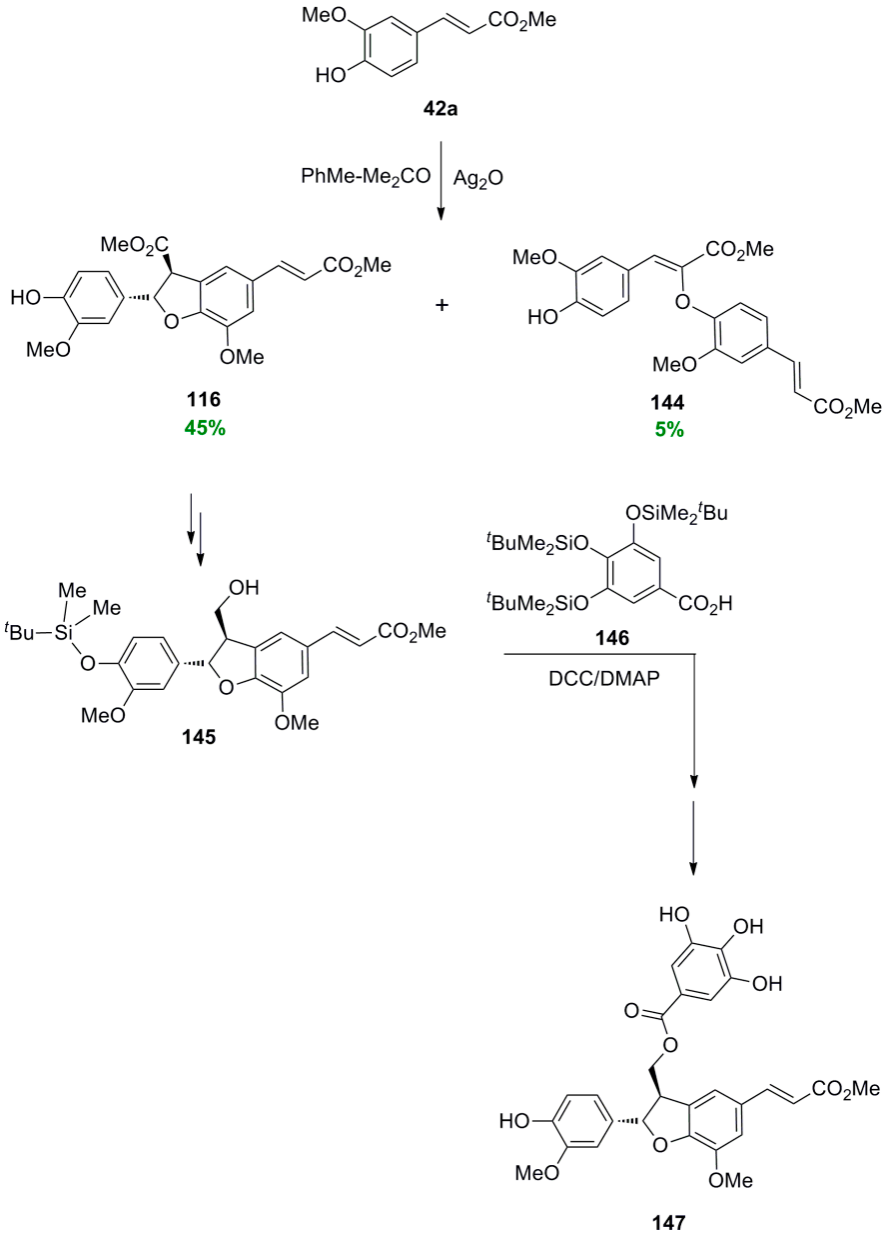
Ag_2_O-mediated POC of Me-Fer and application to the synthesis of the dihydrobenzofuran-gallic acid hybrid **147**.

Rakotondramanana and coworkers subjected Me-Fer (**42a**) to POC using Ag_2_O as oxidant in toluene-acetone (2:1) and obtained as the main product the known dihydrobenzofuran **116** in 45% yield, along with the corresponding β-*Ο*-4 dimer **144** in 5% yield. Compound **116** was protected with the TBDMS group and then selectively reduced to produce alcohol **145**. This compound, upon esterification with the gallic acid derivative **146** and finally deprotection gave the dihydrobenzofuran-gallic acid hybrid **147** (Scheme 56) [81]. Biological evaluation of these compounds for potential antiatherogenic, antiplasmodial and cytotoxic activities showed that dimer **144** presented the best antiatherogenic effect (antioxidant activity and cytoprotective effect) whereas dihydrobenzofuran **116** exhibited the best activity against murine P388 leukemia cells. On the other hand, hybrid **147** showed a very good antiplasmodial potency coupled also with an antioxidant one.

**Figure 6:**
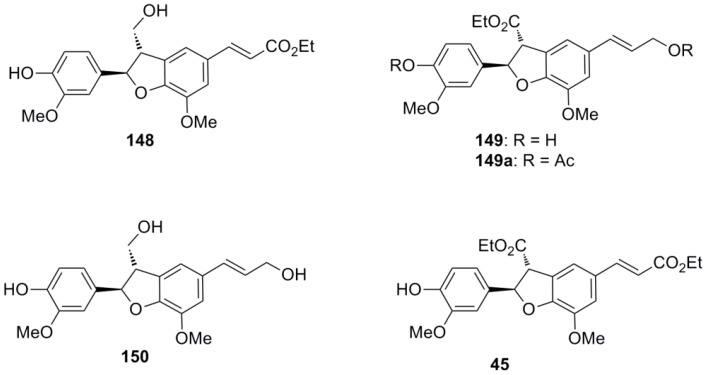
Cross- and homo-coupled products from the cross-POC of Et-Fer with ConAl utilizing the HRP/H_2_O_2_-urea oxidant system.

Zhang and coworkers performed radical coupling reactions between equimolar quantities of Et-Fer (**42**) and ConAl in the presence of HRP and H_2_O_2_-urea complex as oxidant in acetone-aqueous buffer pH 5.0. They isolated and characterized a series of cross-coupled products, two of which (**148** in 6.4% yield and **149**, as the corresponding acetate **149a**, in 1.5% yield) (Figure 6) were NLs of the dihydrobenzofuran type, along with a series of homo-coupled products [82]. Homodimers of the same type, such as dehydrodiconniferyl alcohol **150** and diethyl dehydrodiferulate **45**, were also identified in the reaction mixture.

**Scheme 57:**
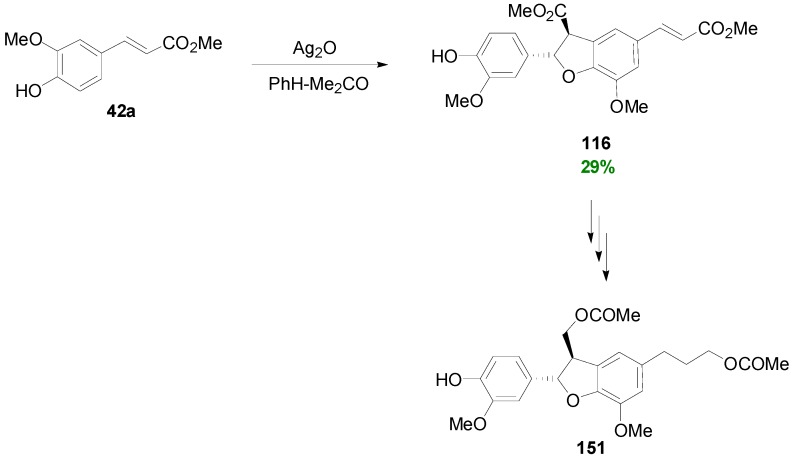
Total synthesis of (±)-tiruneesiin (**151**) utilizing Ag_2_O-mediated POC of Me-Fer (**42a**) as key step.

Subbaraju and coworkers synthesized the neolignan tiruneesiin (**151**) in racemic form using as key reaction the POC of Me-Fer (**42a**) with Ag_2_O in dry PhH-acetone, which produced dihydrobenzofuran **116** in 29% yield. Catalytic hydrogenation of the double bond, LAH-mediated reduction of the ester functions, followed by O-benzylation of the aromatic hydroxyl, bisacetylation of the aliphatic hydroxyl groups and finally catalytic hydrogenolysis afforded (±)-**151** in 12.6% total yield (Scheme 57) [83]. It should be noted that tiruneesiin, identified in *Justicia neesii* Ramamoorthy as the (−)-**151** enantiomer, belongs to the group of dihydrobenzofuranoid NLs, which are known to be inhibitors of tubulin polymerization [77].

**Scheme 58:**
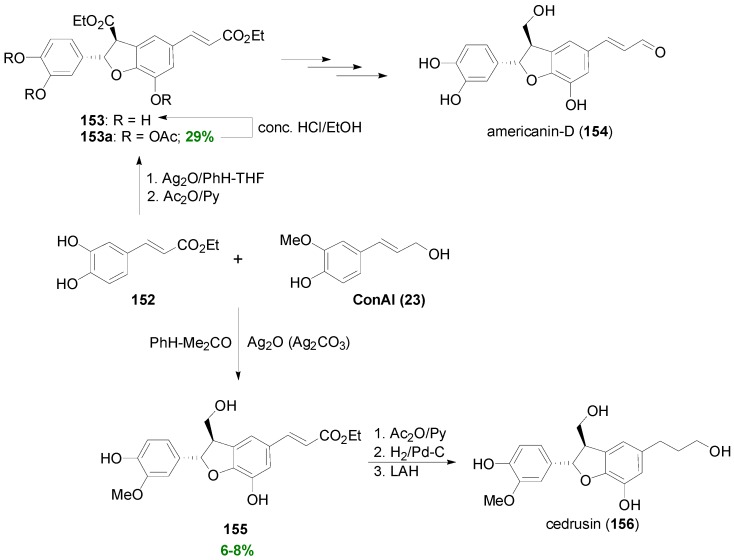
Total synthesis of (±)-americanin-D (**154**) and -cedrusin (**156**) utilizing Ag_2_O-mediated POC of either Et-Caf (**152**) alone or in mixture with ConAl (**23**), respectively, as key step.

Antus and coworkers have shown that Ag_2_O-mediated POC of Et-Caf (**152**) leads to the dihydrobenzofuran **153** as the main product isolated as its corresponding triacetate (**153a**), which was used for the synthesis of the NL Americanin-D (**154**) [84]. When a mixture of **152** and ConAl (**23**) was treated with either silver oxide or silver carbonate in benzene-acetone, the cross-coupled product **155** was isolated in 6%–8% yield, along with other dimers [85]. Compound **155** was used by the same research group as starting material for the synthesis of racemic cedrusin (**156**) (Scheme 58) [86].

**Scheme 59:**
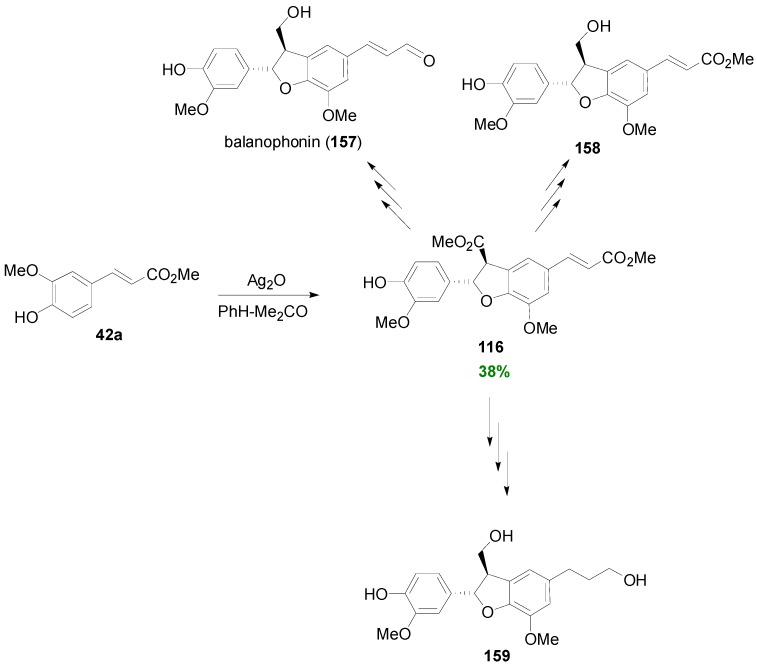
Total synthesis of NLs 157–159 utilizing Ag_2_O-mediated POC of Me-Fer (42) as key step.

On the other hand, the same research group oxidatively dimerized Me-Fer (**42a**) with Ag_2_O in benzene-acetone to obtain dihydrobenzofuran **116** in 38% yield. This compound was employed as starting material for the synthesis of racemic balanophonin (**157**) and its corresponding methyl ester **158** [87] as well as the monomethyl ether **159** of cedrusin [86] (Scheme 59). It should be noted that the NL (−)-balanophonin and its corresponding methyl ester were isolated from *Balanophora japonica* Mikino and *Ziziphus jujuba* Mill, respectively, and showed significant PGI_2_ inducing effect, whereas (+)-cedrusin and its monomethyl ether were isolated from *Cedrus deodara* and *Euconomia ulmoides* Oliv., respectively. The latter plant has been applied in China as hypotensive drug.

**Scheme 60:**
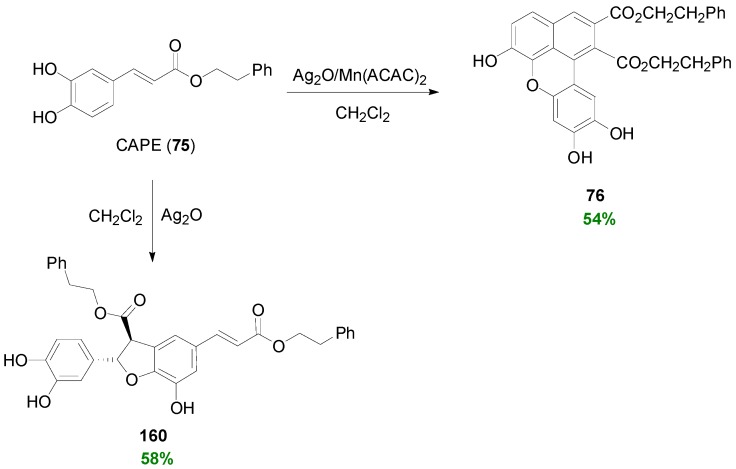
POC of CAPE (**75**) using Ag_2_O alone or Ag_2_O in the presence of Mn(ACAC)_2_ as oxidant.

Daquino and coworkers subjected CAPE (**75**) into POC using Ag_2_O as oxidant in CH_2_Cl_2_ and obtained the corresponding dihydrobenzofuran **160** in 58% yield (Scheme 60) [59] when a ratio of CAPE:Ag_2_O = 1:2 was used. Interestingly, when POC was repeated in the presence of Mn(ACAC)_2_ no dihydrobenzofuran was formed, the main product being the benzoxanthene lignan **76** in 54% yield.

**Scheme 61:**
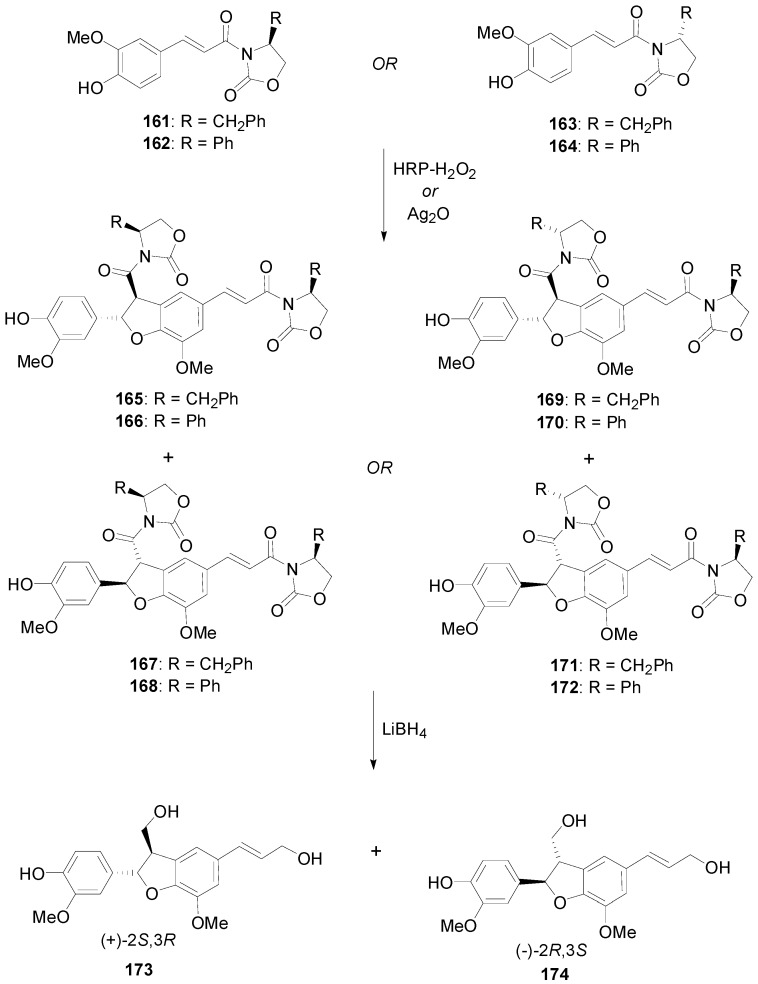
Stereoselective POC of amides **161**–**165** of FerA with 2-oxazilidinones using either Ag_2_O or HRP-H_2_O_2_ as oxidant.

Bruschi and coworkers oxidized Evans’ 2-oxazolidinone amides (**161**–**164**) of FerA utilizing either HRP-H_2_O_2_ in dioxane or acetone/aqueous buffer pH 3.5 or Ag_2_O in CH_2_Cl_2_ as oxidants and obtained the corresponding diastereomeric dihydrobenzofurans **165**–**172**. These compounds were further reduced with LiBH_4_ to the corresponding known enantiomeric dihydrodiconiferyl alcohols **173** and **174** (Scheme 61). The major enantiomer was identified in each case and the e.e. was calculated. Best enantioselectivities (e.e. 59%–62%) were secured with HRP-H_2_O_2_ in acetone-aqueous buffer as the oxidant system and (*R*)- and (*S*)-5-phenyl-2-oxazolidinones as the chiral auxiliaries [88].

**Scheme 62:**
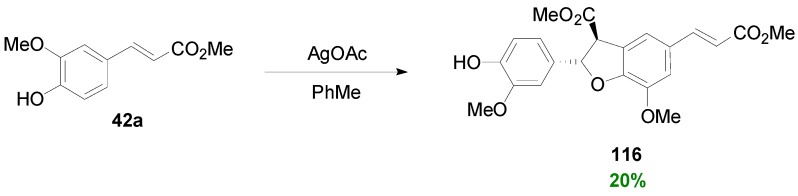
POC of Me-Fer using AgOAc as oxidant.

Snyder and Kontes subjected Me-Fer (**42a**) into POC utilizing silver acetate as the oxidant in toluene and obtained a complex mixture of products from which the dihydrobenzofuran **116** was isolated in 20% yield (Scheme 62) [56].

**Figure 7:**
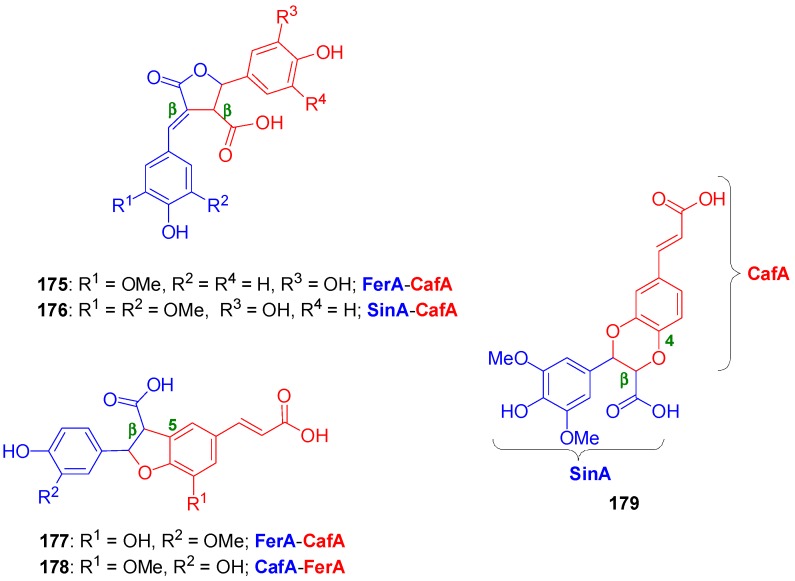
Major cross-coupled products from POC of pairs of HCAs using peroxidase-H_2_O_2_ as oxidant.

Arrieta-Baez and Stark subjected pairs of HCAs in cross-POC utilizing H_2_O_2_ in combination with either APP or HRP, as the oxidant systems in a phosphate buffer (pH 6.1) and studied the formation of products from cross-coupling and dimerization reactions. They found that only the mixtures of CafA and FerA and of CafA and SinA produced cross-coupled products in addition to dimerization products. In combinations of CouA and any of the other HCAs, dimerization products were only observed from the other HCAs but not CouA. Also, from the mixture FerA and SinA dimerization products were only observed from SinA. Both enzymes produced identical major products. The various major products formed were isolated by HPLC and identified. They had predominantly β-β γ-lactone (**175**–**176**) and β-5 benzofuran molecular frameworks (**177**–**178**) and one of them (**179**) was of the oxyneolignan 1,4-benzodioxane type (Figure 7) [89].

**Figure 8:**
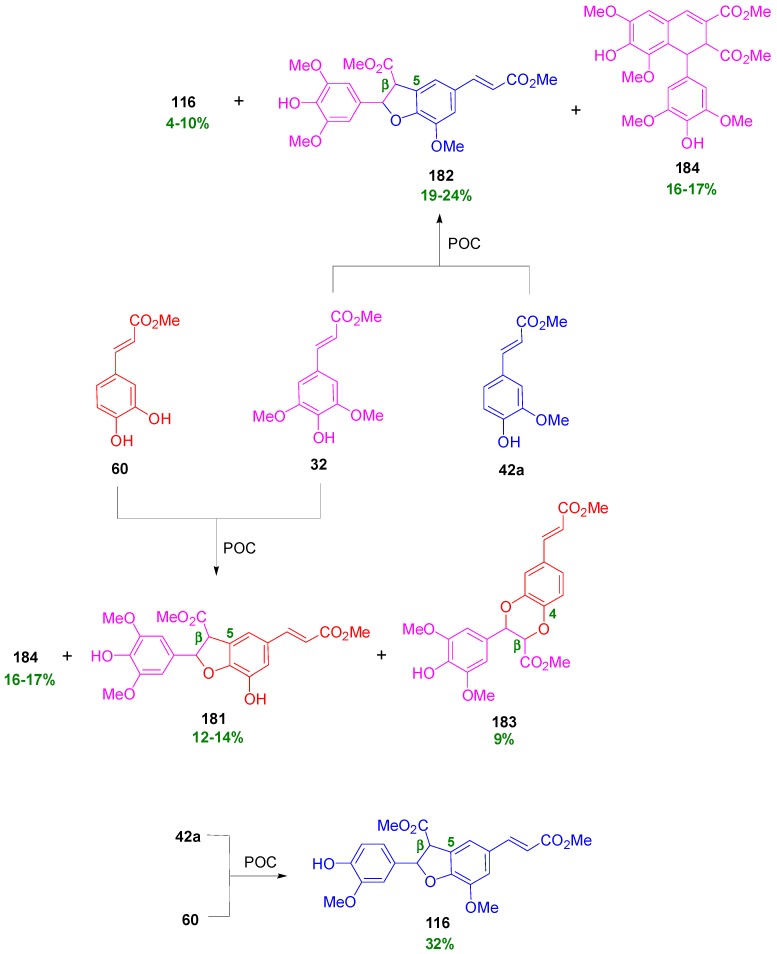
Major dimers obtained from POC of pairs of methyl esters of HCAs using peroxidase-H_2_O_2_ or MnO_2_ as oxidant.

Saliu and coworkers also performed cross-coupling reactions in pairs of equimolar methyl esters (**60**, **42a** and **32**) of the HCAs CafA, FerA and SinA and of the amides **180** and **181** of FerA and SinA, respectively) with the chiral auxiliary (*R*)-methylbenzylamine, utilizing the HRP-H_2_O_2_ oxidant system in dioxane-aqueous buffer pH 3.2 or MnO_2_ in CH_2_Cl_2_. The two oxidant systems gave, in general, comparable results. Cross-coupling products, along with homodimers, were essentially obtained in 12%–24% yields from the combinations of **32** with **60** and of **32** with **42a**. They were predominantly of the benzofuran type (**181**–**182**). In one case, a 1,4-benzodioxane product (**183**) was also formed in 9% yield. The combination of **60** and **42a** produced as main product in 32% yield the homodimer benzofuran **116** from **42a**. Homodimers obtained were either of the benzofuran (**116**) or the dihydronaphthalene (**184**) type (Figure 8) [90].

**Scheme 63:**
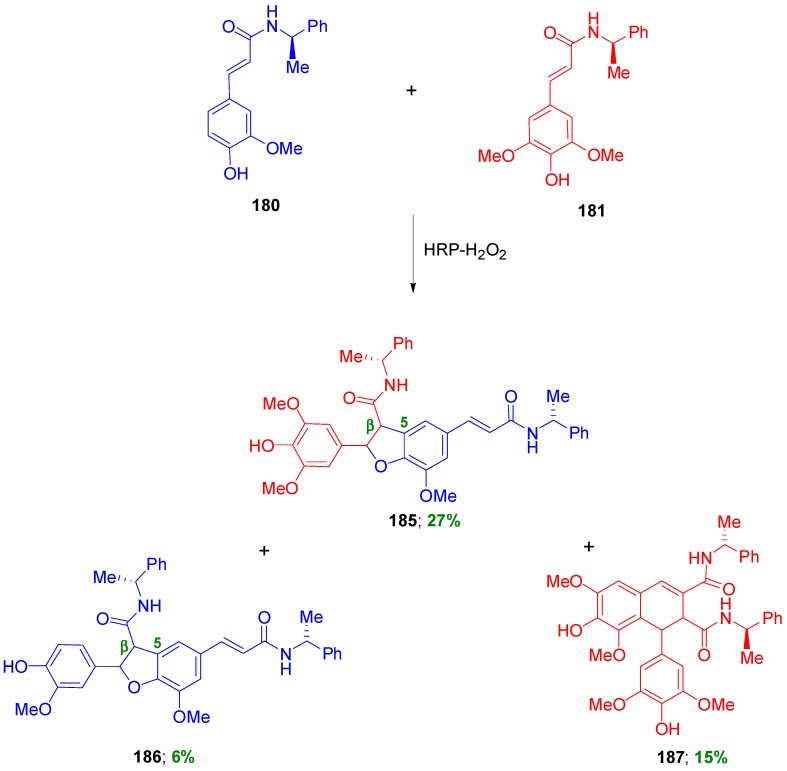
Products from POC of chiral amides of HCAs using peroxidase-H_2_O_2_ as oxidant.

On the other hand, the combination of amides **180** and **181** produced mainly a benzofuran type cross-coupled product **185** in 27% and two homodimers, one of the benzofuran type (**186**) and the other of the dihydronaphthalene type (**187**) in 6 and 15% yields, respectively (Scheme 63). Product **185** was a mixture of diastereomers with a d.e. evaluated to be 38%.

**Figure 9:**
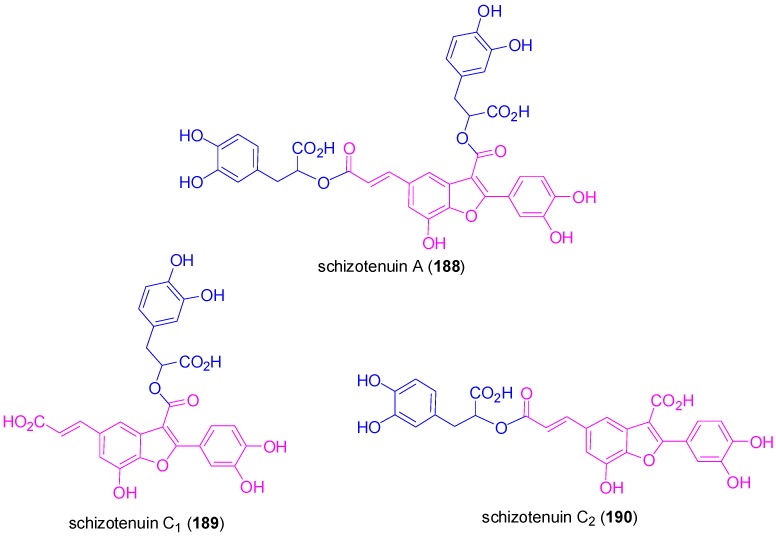
The structure of schizotenuins.

**Scheme 64:**
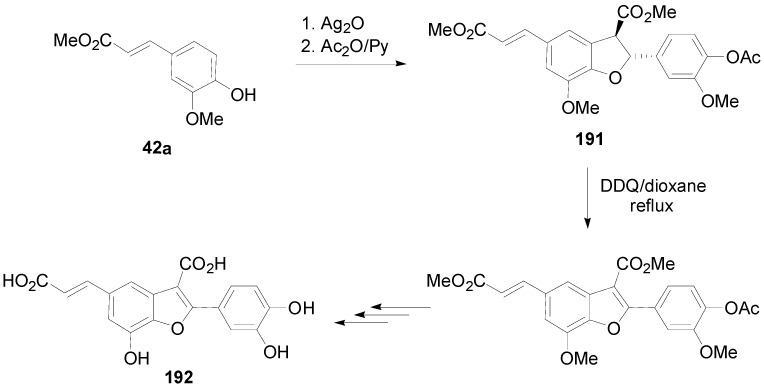
Synthesis of the benzofuran core (**192**) of schizotenuins.

Interestingly, conjugation of NLs of the benzofuran type with other natural products can lead to hybrids with interesting biological properties, such as the dihydrobenzofuran-gallic acid hybrid **147** (Scheme 56) mentioned above. Other worth mentioning such hybrids are the so called schizotenuins A (**188**), C_1_ (**189**) and C_2_ (**190**) (Figure 9) which were isolated by Matsuta and coworkers from the terrestrial part of *Schizonepeta tenuifolia* BRIQ. This plant has been used in traditional Chinese medicines as anti-inflammatory crude drug. Hybrids **188**–**190** are actually the diester and the two possible monoesters of the benzofuran **192** (CafA dimer) with DplA and act by inhibiting the 3α-hydroxysteroid dehydrogenase. Compound **192** was prepared from the fully protected dihydrobenzofuran **191**, readily available using Ag_2_O-mediated POC of Me-Fer (**42a**) as key reaction, by DDQ-mediated oxidation, followed by deprotection steps (Scheme 64) [73].

**Scheme 65:**
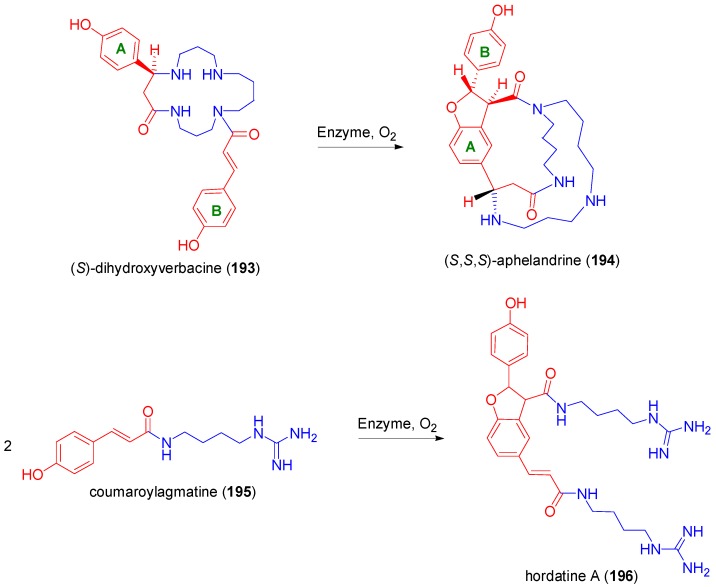
The PA alkaloids **194** and **196**, incorporating dihydrobenzofuranoid substructures, arising from enzyme-catalyzed POC of CouA moieties. The PA moieties are drawn in blue.

A dihydrobenzofuran unit constitutes the central substructure of the bicyclic Spm alkaloid (*S*,*S*,*S*)-aphelandrine (**194**) which is present in *Aphelandra* plants (Acanthaceae). Nezbedovà and coworkers has obtained this alkaloid in preparative yield through a stereoselective intramolecular POC of the Spm-CouA hybrid (*S*)-dihydroxyverbacine (**193**) in the presence of the soluble protein fraction of barley seedlings (*Hordeum vulgare*, Gramineae) as catalyst and O_2_ as oxidant (Scheme 65) [91]. The PA alkaloid (+)-hordatine A (**196**), isolated from barley (*Hordeum vulgare* L.), has a similar benzofuranoid substructure. It had been previously synthesized enzymatically from coumaroylagmatine (**195**) using cell free extracts from the shoots of barley seedlings [92].

**Figure 10:**
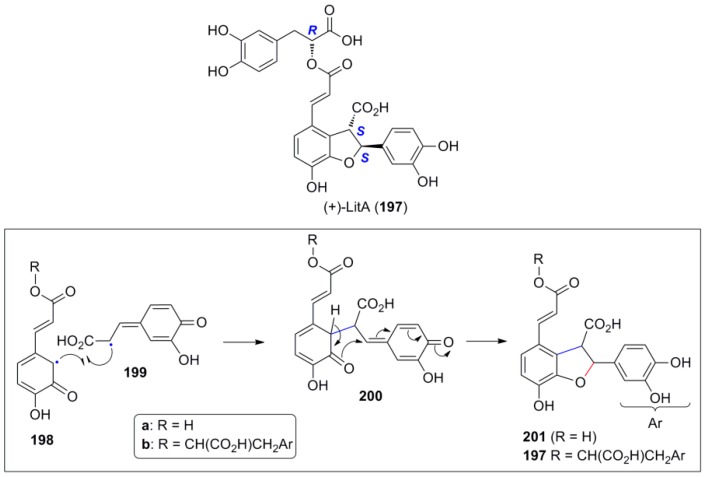
The structure of (+)-LitA (**197**) and outline of plausible mechanism for the formation of core dihydrobenzofuranoid structure by either POC of CafA or cross-POC of CafA with RosA.

Another interesting, naturally occurring, dihydrobenzofuranoid hybrid is (+)-LitA (**197**) (Figure 10), in particular because of its potent and non-toxic anti-HIV activity that results from inhibition of HIV-1 integrase [93]. Several total syntheses of **197** have been already reported [93,94,95,96,97]. This compound can be considered as a hybrid of the (2*S*,3*S*)-dimer **201** of CafA with (*R*)-DplA. Dimer **201** could be assembled in nature through the unusual coupling (β-2) of the C-centered radicals **198** and **199**, followed by the usual Michael addition on the *p*-quinonemethide intermediate **200**. Alternatively, LitA might be considered as the product (hybrid) of cross-POC between RosA and CafA, involving the same type of C-C bond formation as key step for the assembly of the molecular skeleton.

**Figure 11:**
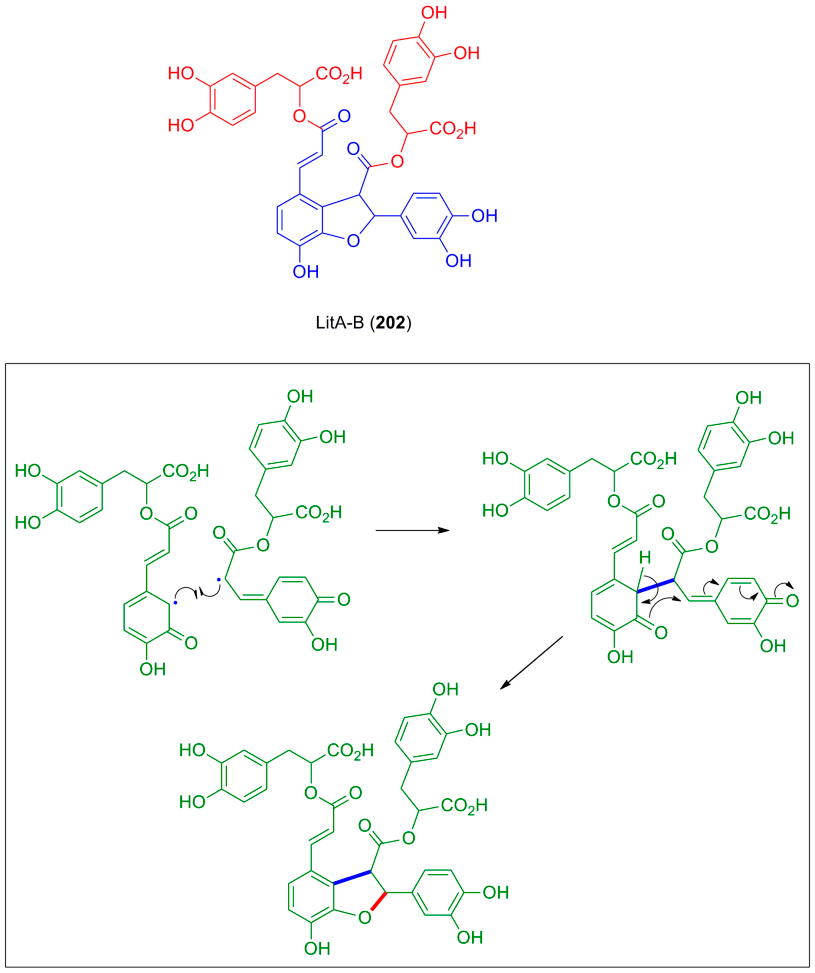
The structure of LitA-B (**202**) and outline of the possible mechanism of its formation by oxidative cyclization through POC between two molecules of RosA.

RosA and LitA along with dimers and trimers of the former, in which the monomeric units are joined through dihydrobenzofuran moieties, were isolated and identified by Ly and coworkers from the methanol exctract of dried leaves of *Celastrus hindsii* Benth a species of the Celastraceae family, which is used in Vietnam as traditional medicine for treating ulcers, tumors and inflammation. These compounds showed antioxidative activity whose effectiveness could be correlated to the number of the phenolic hydroxyl groups in each molecule [98]. One of the dimers, namely LitA-B (**202**) may be considered as a hybrid of neolignan **201** with two molecules of DplA (Figure 11). It is thought of being formed in nature through POC coupling of two molecules of RosA. LitA-B is also a potent and nontoxic inhibitor of HIV-1 replication. The pharmaceutically interesting acids RosA, LitA and LitA-B have been also found in some other plants [98].

**Figure 12:**
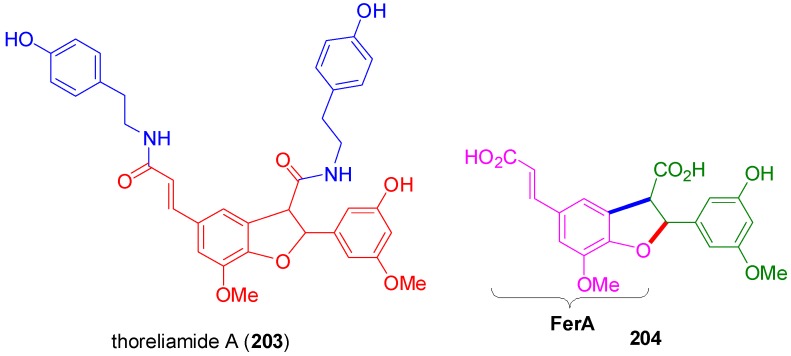
The structure of thorielamide A (**203**).

Another example of a dihydrobenzofuranoid hybrid is thoreliamide A (**203**) (Figure 12) which has been isolated in optically inactive form from the stems of the plant *Mitrephora thorelii* (Annonaceae) by Ge and coworkers [58]. This natural product may be considered as the hybrid of the unusual dihydrobenzuran **204** with two molecules of tyramine. Compound **204** might arise from cross-POC of FerA and SinA or HFeA with further functional group elaboration on the aromatic SinA/HFeA side of the molecule.

### 3.2. Synthesis of Oxyneolignans of the Ether Type—Formation of β-O-4 Bond as the Key Step

**Figure 13:**
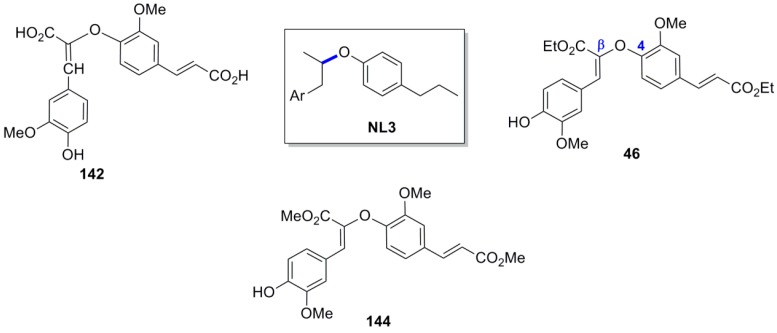
The structure of FerA and Et-Fer dimers **142**, **46** and **144** respectively, as examples of the NL3 subtype.

As we noted above, Carunchio and coworkers subjected FerA to POC using the enzyme laccase and identified two main products initially formed, a benzofuranoid dimer **141** and the β-*O*-4 coupling product **142** (Scheme 54) [78]. The latter product is an example of the NL3 subtype of NLs (Figure 13). Οn the other hand, Neudorffer and coworkers isolated the oxyneolignan ether **46** (Scheme 28 and Figure 13) in 10% yield, among other lignans, from the reaction mixture of the electrochemical POC of Et-Fer [43]. Furthermore, Rakotondramanana and coworkers isolated the corresponding dimethyl ester **144** (Scheme 56 and Figure 13) in very low yield, along with the dihydrobenzofuranoid FerA dimer **116**, from the Ag_2_O-mediated POC of Me-Fer [81].

**Figure 14:**
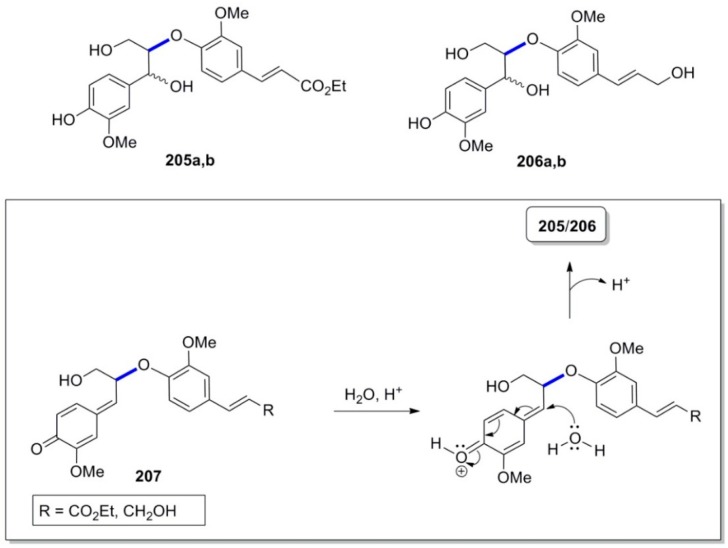
Cross (**205**)- and homo (**206**)-coupled products of the β-*Ο*-4 ether type from the cross-POC of Et-Fer with ConAl and outline of mechanism of their formation.

Cross (e.g., **205a**,**b**)- and homo (**206a**,**b** and **46**)-coupling products (Figure 14) of the β-*Ο*-4 ether type from the cross-POC of Et-Fer and ConAl (**23**) were isolated and/or identified, among other lignans, by Zhang and coworkers [82]. Compounds **205** and **206** are obviously formed through interception of the *p*-benzoquinonoid intermediates **207** by water.

Lu and coworkers subjected Et-Fer in POC utilizing the complex CuCl(OH)-TMEDA as catalyst and oxygen as oxidant in acetonitrile and obtained a mixture composed of β-*O*-4, 8-5, 8-8 (cyclic and noncyclic) and 5-5 coupled dehydrodiferulates, from which the β-*O*-4 dimer **46** was obtained through FCC along with the β-5 (dihydrobenzofuranoid) dimer **45** (the structure of dimers **45** and **46** can be seen in Scheme 28). Crystallization of the mixture resulted to pure crystalline **45** and left **46** in mother liquor. From the latter, pure dimer **46** was obtained through simple treatment of mother liquor with TBAF, which converted the remaining dimer **45** to a noncyclic β-5 dimer, and finally rechromatography [99].

**Figure 15:**
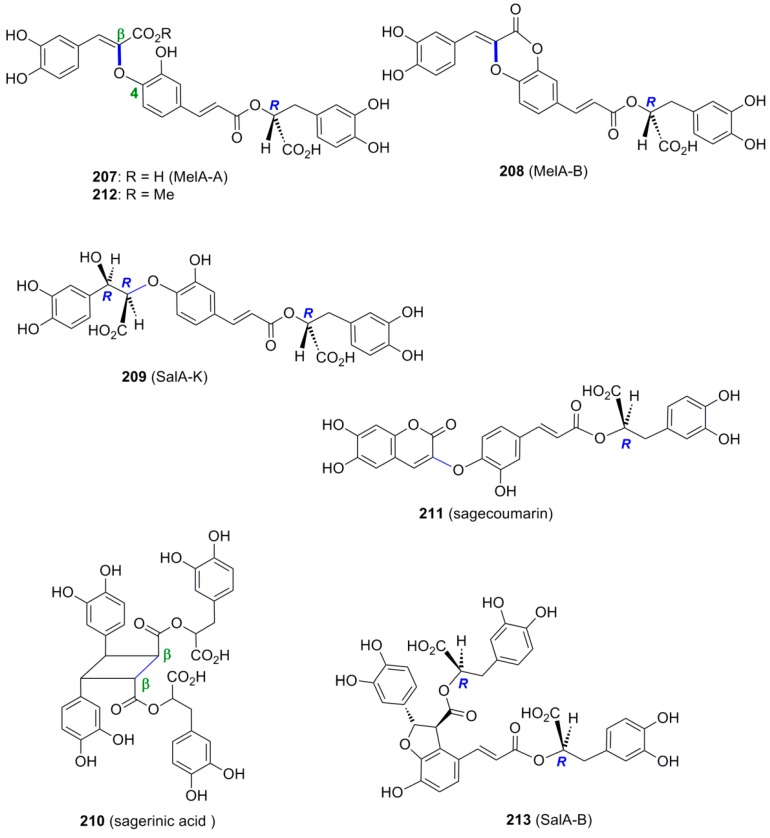
The structures of melitric and salvianolic acids as well as of sagerinic acid and sagecoumarin.

Interestingly, several oxyneolignan hybrids with interesting biological activities have been isolated from plants. For example, Agata and coworkers isolated from the overground part of *Melissa officinalis* L., a labiate plant used as folkmedicine in Europe for the treatment of chronic bronchial catarrh, feverish cold, headaches and tension, in addition to RosA two new compounds consisted of three CafA units which they named MelA-A (**207**) and -B (**208**) (Figure 15) [100]. The former compound may be thought of deriving from RosA and CafA through cross-POC with a β-*Ο*-4 bond connecting the two molecules. Althernatively, compound **207** may be considered as a hybrid of β-*O*-4 dimeric CafA and DplA. MelA-B is a product of an intramolecular dehydration of MelA-A.

Lu and Foo isolated from *Salvia officinalis* (Sage), a popular herb which has been used since ancient times for its health giving properties and for treating all kinds of ailments, in addition to RosA two compounds, namely the known SalA-K (**209**) and a novel cyclobutane derivative named sagerinic acid (**210**) (Figure 15) [101]. The former might be formed by cross-POC of RosA with CafA during which the primary β-*Ο*-4 bond is formed, followed by a stereoselective interception of the derived *p*-benzoquinone intermediate by water. In the latter, the two RosA units may be first connected by a Cβ-Cβ bond through POC, probably followed by an intramolecular Michael addition to create the second C-C bond with ring closure and finally reduction of the intermediate *o*-benzoquinone formed. From the same plant, Lu and coworkers isolated another novel CafA trimer, which they named sagecoumarin (**211**) (Figure 15), and methyl melitrate A **(212**) and proposed that MelA-A, its ester **212** and **211** are all synthesized from SalA-K [102].

Another SalA, namely SalA-B (**213**) (Figure 15) has been isolated from *Salvia miltiorrhiza* Bge, a well-known Chinese medicine for treating and preventing aging diseases for throusands of years, and has been shown to present interesting cardiovascular effects [103] and to be a potential chemoprotective agent for head and neck squamous cell cancer [104]. SalA-B is structurally related to LitA.

**Figure 16:**
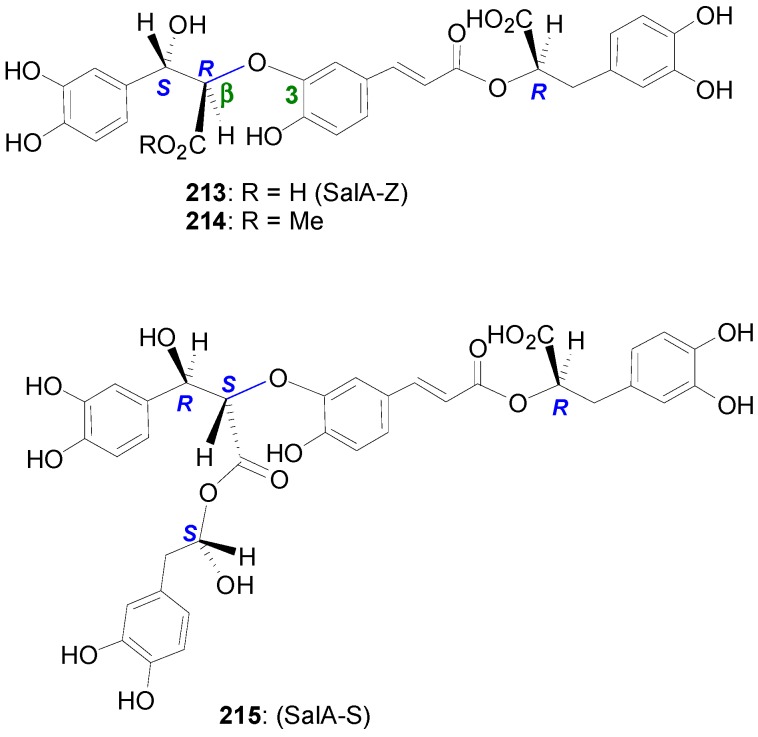
The structures of SalA-Z and SalA-S and of the methyl ester **214** of the former.

Exarchou and coworkers isolated from *Origanum dictamnus* L. (Lamiaceae), an endemic plant of Crete which was considered by ancient Greeks as Panacea and used in many cases for its healing effects, in addition to a new dihydronaphthalene hybrid SalA-R (**73**, Figure 2), RosA and MelA-A three new hybrids **213**–**215** of dimeric CafA, with an unusual connection (β-*Ο*-3), and DplA (Figure 16). Interestingly, SalA-S (**215**) was the most potent antioxidant, followed by RosA, in the DPPH assay used [57].

### 3.3. Synthesis of Oxyneolignans of the 1,4-Benzodioxane Type—Formation of β-O-4 Bond as the Key Step

**Figure 17:**
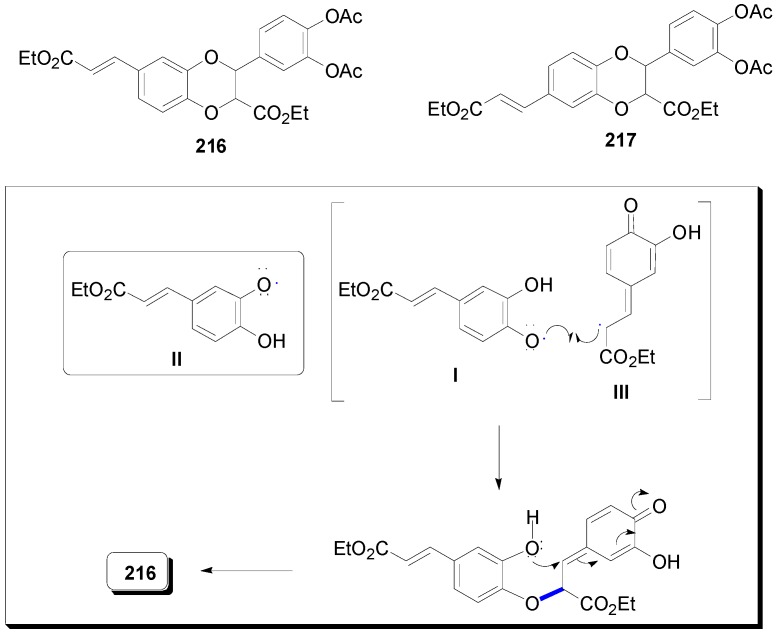
Structure of oxyneolignans **216** and **217** and outline of the mechanism of formation of regioisomer **216**. Isomer **217** might be formed from the alternative phenoxy radical **II**.

As we noted above, Antus and coworkers oxidatively dimerized Et-Caf (**152**) using Ag_2_O as oxidant and then acetylated the crude reaction product to obtain pure triacetate **153a** in 29% yield following crystallization (Scheme 58). From the mother liquor, the authors isolated through silica gel CC a mixture of the isomeric oxyneolignans **216** and **217** (Figure 17) of the 1,4-benzodioxane NL2 type in 6% yield [84].

**Scheme 66:**
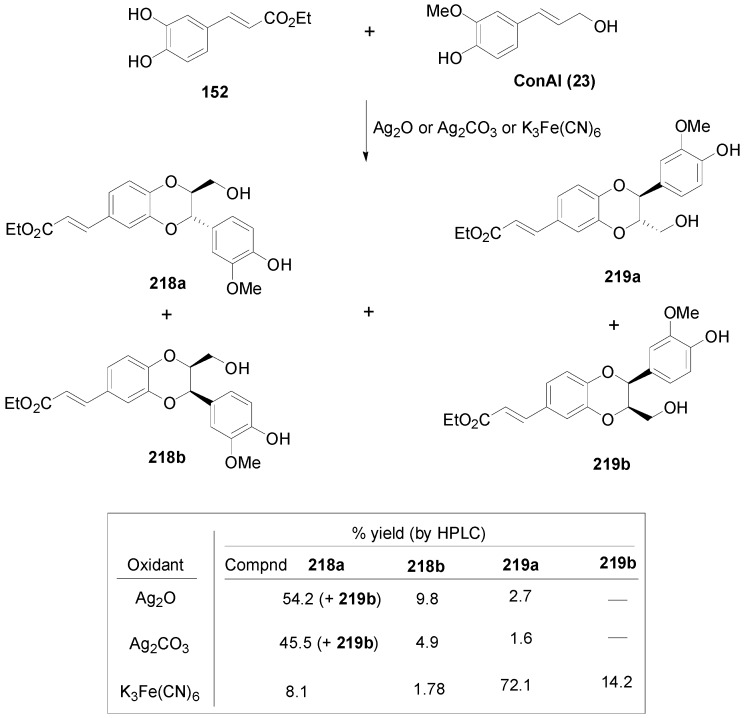
Cross-POC between Et-Caf and ConAl (**23**).

The same research group subjected a mixture of Et-Caf (**152**) and ConAl (**23**) (molar ratio = 1:1.1) in cross-POC using silver oxide or silver carbonate as oxidant in benzene/acetone (2:1) and obtained a mixture from which the various products were isolated by preparative TLC. Major product of the coupling reaction was the 3-aryl-1,4-benzodioxane derivative **218a**, which crystallized out from a mixture with the alternative regioisomer **219a**. Both had the *trans* relative stereochemistry. Their corresponding *cis* isomers **218b** and **219b** were also obtained in much lower yields (Scheme 66). Silver carbonate resulted in higher regioselectivity (**218a**:**219a** = 25:1) than that obtained with silver oxide (**218a**:**219a** = 19:1). On the other hand, the use of hexacyanoferrate(III)/sodium carbonate as oxidizing agent in acetone/water (*ca.* 1:3) reversed the regioselectivity of the reaction and produced **219a** as the major isomer. The *cis* isomers were also isolated as crystalline compounds in lower yields.

The **218** (**a** + **b**):**219**(**a** + **b**) ratio was *ca.* 1:9 [85].

**Figure 18:**
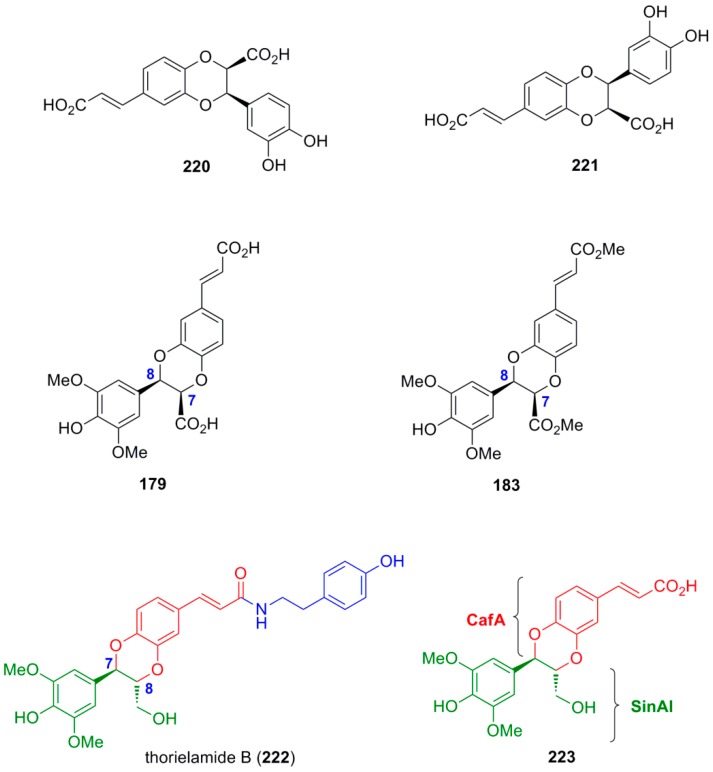
Structures of oxyneolignans of the 1,4-benzodioxane type.

POC of CafA with O_2_ in an alkaline (KOH) aqueous solution produced, in addition to caffeicins E and F (see sections 2.2 and 3.1 above), dimeric CafA compounds of the 1,4-benzodioxane type which were named caffeicin A–D. The two substituents of the heterocyclic ring had the *cis* relative configuration (see e.g., compounds **220** and **221**) (Figure 18) based on ^1^H-NMR coupling constants considerations [45]. The *cis* formed is preferred since the bulky groups are both in the equatorial position with the dioxane ring adopting the boat form.

As mentioned above, when a mixture of CafA and SinA was subjected in cross-POC utilizing H_2_O_2_ in combination with either APP or HRP, an oxyneolignan (**179**) of the 1,4-benzodioxane type was obtained (Figure 7 and Figure 18) in modest yield [89].

The reported coupling constant (*J*) for protons 7 and 8 was 2.5 Hz. Therefore, the relative stereochemistry in compound **179** should be *cis* (Figure 18). On the other hand, cross-POC of a pair of Me-Caf and Me-Sin utilizing the HRP-H_2_O_2_ oxidant system produced, among other lignans, the 1,4-benzodioxane product **183** in 9% yield (Figure 8) [90]. Based on coupling constant arguments (*J*_H7–H8_ = 2.3 Hz), this compound should also have the *cis* relative stereochemistry (Figure 18).

An interesting optically inactive 1,4-benzodioxane hybrid **222** (Figure 18) was isolated, along with other lignanamides (see Section 2.2 and Section 3.1) from the stems of *Mitrephora thorelii* [58]. This compound, named thoreliamide B, can be considered as the hybrid of acid **223** and tyramine. The former may then obviously be a cross-POC product of SinAl with CafA. Based on coupling constant arguments (*J*_H7–H8_ = 8.0 Hz), this racemic compound should have the *trans* relative stereochemistry.

### 3.4. Synthesis of Other NLs—Formation of β-1 or 5-5 Bond as the Key Step

**Scheme 67:**
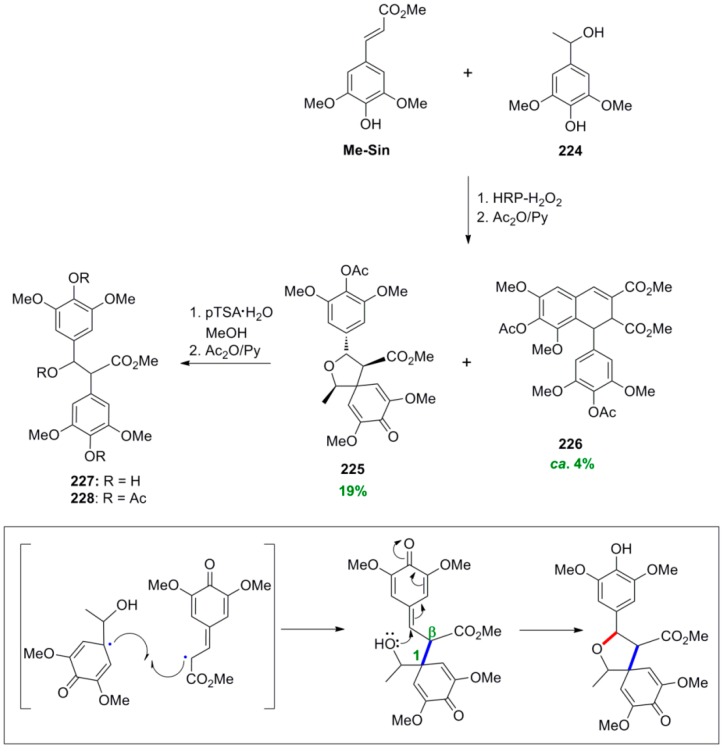
Cross-POC between Me-Sin and alcohol **224**.

Setälä and coworkers performed a cross-POC reaction with an equimolar mixture of Me-Sin (**32**) and alcohol **224**, a syringyl lignin model compound, utilizing HRP-H_2_O_2_ as oxidant system in acetone-aqueous buffer solution pH 3.5. Acetylation of the crude reaction mixture, followed first by silica gel CC and then by preparative HPLC separation, lead to a *ca.* 50% recovery of alcohol **224** and the isolation of two products, namely the spiro cross-coupling product **225** in 19% yield and the sinapate dimer **226** in *ca.* 4% yield (Scheme 67) [105]. Treatment of spiro compound **225** with a catalytic quantity of pTSA monohydrate in methanol gave, with loss of the side chain, a single diastereomer of compound **227**, which was isolated in 33% yield as the corresponding peracetate **228**.

**Figure 19:**
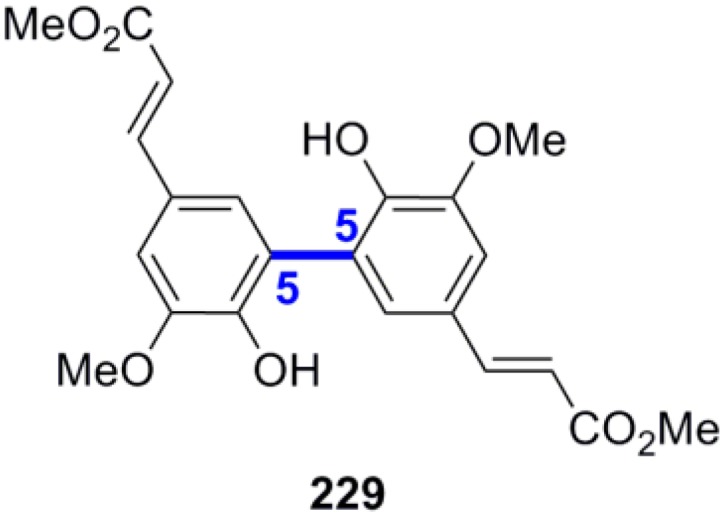
The structure of 5-5 coupled dimethyl dehydrodiferulates (**229**).

As we noted above, Lu and coworkers subjected Et-Fer in POC utilizing the complex CuCl(OH)-TMEDA as catalyst and O_2_ as oxidant in acetonitrile and obtained a mixture composed of β-*O*-4, 8-5, 8-8 (cyclic and noncyclic) and 5-5 coupled dehydrodiferulates. From this mixture, they isolated not only the β-*Ο*-4 and the 8-5 coupled dehydrodiferulates (see Scheme 28) but also the 5-5 coupled diferulate (**229**) (Figure 19) and the 8-8 coupled cyclic (**43**) and noncyclic (**44**) dehydrodiferulates (see Scheme 28) by flash chromatographic fractionation [99].

## 4. Conclusions

We have presented in this review an overview of past and recent applications of an old reaction, namely POC, in the bioinspired syntheses of a variety of CLs, and NLs with potential medicinal significance. These involve, in particular, lignans of the dilactone, dihydronaphthalene, bis(aryl-methylene)succinic acid, 1,4-benzodioxane, tetrahydrofuran, dihydrobenzofuran, aryl β-cinnamyl ether, and benzoxanthene subtypes. POC utilizes simple starting materials and experimental procedures and creates in one-pot experiments relatively complex structures often with good yields, which would otherwise require several steps to be assembled. Simple, commercially available or easily synthesized, building blocks, such as naturally occurring HCAs and HCAls and synthetic analogs as well as esters and amides of HCAs, are usually employed. Coupling between the same or different (cross-POC), in some cases, building blocks is effected by the action of one-electron inorganic oxidants or enzymes (peroxidases, usually HRP and less often laccases) as catalysts with H_2_O_2_ or O_2_ as oxidant, which mimic even closer the natural conditions for performing such dehydrodimerizations. Suitable inorganic oxidants have been also used in the so-called non-POC which uses ACAs as starting materials and has been used successfully, for example, in the synthesis of lignans of the isostegane type. Although POC usually produces mixtures of regio- and diastereoisomers and therefore chromatographic techniques are necessary for their separation, it is still a very attractive alternative to other multi-step methodologies, in particularly in cases where a single regioisomer and/or diastereoiosmer is formed as the main product, because of the simplicity in its performance and the fact that creates complex structures in one-pot reactions. The regioselectivity of POC has been improved by selectively blocking positions which could be potentially involved in bond-forming reactions through radical coupling. One such group is the *tert*-butyl group which is readily introduced and finally removed after the POC reaction. Interestingly, the primary products of POC can be readily transformed through simple reactions in a multitude of other medicinally significant lignans and hybrids. A serious drawback of POC is that it provides the dimers in racemic mixtures, even when enzymes are used as catalysts. However, this problem has been faced with considerable success by subjecting to POC chiral esters or amides of HCAs, readily produced by condensing HCAs with chiral alcohols or amines, e.g., esters of chiral α-amino acids, used as chiral auxilliaries. That way, the thus produced diastereomeric compounds are resolved with chromatographic techniques and, if necessary, the chiral auxilliary is removed, for example, by alkaline hydrolysis or LiOOH-mediated hydrolysis. It is reasonable to assume that POC will continue to be used for the ready assembly of the skeleta of naturally occurring and biologically interesting lignans of several types and that it will be further developed towards the direction of improving its regio- and the stereoselectivity by the thoughtful choice of combinations of substrates, oxidant systems, reaction conditions and chiral auxilliaries.

## Abbreviation

ACAs: alkoxycinnamic acids
APP: anionic potato peroxidase
CafA: caffeic acid
CAN: ceric ammonium nitrate
CAPE: phenethyl caffeate
CC: column chromatography
CinA: cinnamic acid
CL: classical lignan
ConAl: coniferyl alcohol
CouA: *p*-coumaric acid
DBU: 1,8-diazabicyclo[5.4.0]undec-7-ene
DCC: *N,N'*-dicyclohexylcarbodiimide
DDQ: 2,3-dichloro-5,6-dicyano-*p*-benzoquinone
DIBAL: diisobutylaluminium hydride
DMAP: 4-(dimethylamino)pyridine
DplA: 3-(3,4-dihydroxyphenyl)lactic acid
DPPH: 2,2-diphenyl-1-picrylhydrazyl
Et-Caf: ethyl caffeate
Et-Fer: ethyl ferulate
Et-Sin: ethyl sinapate
FCC: flash column chromatography
FerA: ferulic acid
Gly: glycine
HCAs: 4-hydroxycinnamic acids
HCAls: 4-hydroxycinnamyl alcohols
HFeA: 5-hydroxyferulic acid
HRP: horse radish peroxidase
LAH: lithium aluminium hydride
LitA: lithospermic acid
Me-Caf: methyl caffeate
Me-Fer: methyl ferulate
MelA: melitric acid
Me-Sin: methyl sinapate
Mn(ACAC)_2_: manganese(II) acetylacetonate
Mn(III)TPPOAc: tetraphenylporphyrinatomanganese(III) acetate
NL: neolignan
non-POC: “non-phenolic” oxidative coupling
PA: polyamine
Phe: Phenylalanine
PIDA: phenyliodonium diacetate
PIFA: phenyliodonium *bis*(trifluoroacetate)
POC: phenol oxidative coupling
Pro: proline
pTSA: *p*-toluenesulfonic acid
RosA: rosmarinic acid
SalA: Salvianolic acid
SARS: structure-activity relationship studies
ShiA: shikimic acid
SinA: sinapic acid
SinAl: sinapyl alcohol
Spm: spermine
TBAF: tetrabutylammonium fluoride
TBDMS: *tert*-butyldimethylsilyl
ThoA: thomasidioic acid
TLC: thin layer chromatography
TMEDA: tetramethylethylenediamine
Tyr: tyrosine

## References

[1] Geissman T.A., Crout D.H.G. (1969). Organic Chemistry of Secondary Plant Metabolism.

[2] Teixera J., Gaspar A., Garrido E.M., Garrido J., Borges F. (2013). Hydroxycinnamic acid antioxidants: An electrochemical overview. BioMed Res. Int..

[3] De P., Baltas M., Bedos-Belval F. (2011). Cinnamic acid derivatives as anticancer agents—A review. Curr. Med. Chem..

[4] Lewis N.G., Davin L.B., Barton Sir D.H.R., Nakanishi K., Meth-Cohn O. (1999). Lignans: Biosynthesis and function. Comprehensive Natural Products Chemistry.

[5] Barton D.H.R., Cohen T. (1957). Some biogenetic aspects of phenol oxidation. Festschrift Arthur Stoll.

[6] Barton D.H.R., Deflorin A.M., Edwards O.E. (1956). The synthesis of Usnic acid. J. Chem. Soc..

[7] Erdtman H. (1933). Dehydrierungen in der coniferylreihe. II. Dehydrodi-isoeugenol. Annalen.

[8] Erdtman H., Wachtmeister C.A. (1957). Phenoldehydrogenation as a biosynthetic reaction. Festschrift Arthur Stoll.

[9] Erdtman H. (1968). Recent Advances in Phytochemistry.

[10] Gotlieb O.R. (1972). Chemosystematics of the lauraceae. Phytochemistry.

[11] Brown B.R., Taylor W.I., Battersby A.R. (1967). Biochemical Aspects of Oxidative Coupling of Phenols. Oxidative Coupling of Phenols.

[12] Scott A.I. (1965). Oxidative coupling of phenolic compounds. Q. Rev. (Chem. Soc.).

[13] Lewis N.G., Sarkanen S. (1998). Lignin and Lignan Biosynthesis.

[14] Moss G.P. (2000). Nomenclature of lignans and neolignans. Pure Appl. Chem..

[15] Setälä H. (2008). Regio- and Stereoselectivity of Oxidative Coupling Reactions of Phenols: Spirodienones as Construction Units in Lignin. Ph.D. Thesis.

[16] Umezawa T., Okunishi T., Shimada M. (1997). Stereochemical diversity of lignan biosynthesis. Wood Res.: Bull. Wood Res. Inst. Kyoto Univ..

[17] Deyama T., Nishibe S., Heitner C., Dimmel D.R., Schmidt J.A. (2010). Pharmacological properties of lignans. Lignins and Lignans: Advances in Chemistry.

[18] Ward R.S. (1982). The synthesis of lignans and neolignans. Chem. Soc. Rev..

[19] Ward R.S. (2003). Different strategies for the chemical synthesis of lignans. Phytochem. Rev..

[20] Pan J.-Y., Chen S.-L., Yang M.-H., Wu J., Sinkkonen J., Zou K. (2009). An update on lignans: Natural products and synthesis. Nat. Prod. Rep..

[21] Pal T., Pal A. (1996). Oxidative phenol coupling: A key step for the biommimetic synthesis of many important natural products. Curr. Sci..

[22] Erdtman H., Phenoldehydrierung V.I. (1935). Dehydrierende Kupplung einiger Guajakoi-derivate. Svensk. Kem. Tidskr..

[23] Cartwright N.J., Haworth R.D. (1944). Constituents of natural phenolic resins. XIX. Oxidation of ferulic acid. J. Chem. Soc..

[24] Takei Y., Mori K., Matsui M. (1973). Synthesis of *dl*-matairesinol dimethyl ether, dehydrodimethyl conidendrin and dehydrodimethyl retrodendrin from ferulic acid. Agric. Biol. Chem. (Jpn.).

[25] Pelter A., Ward R.S., Watson D.J., Collins P., Kay I.T. (1982). Synthesis of 2,6-diaryl-4,8-dihydroxy-3,7-dioxabicyclo[3.3.0]octanes. J. Chem. Soc. Perkin Trans. 1.

[26] Freudenberg K., Schraube H. (1955). Synthese des syringaresinols und versuche mit sinapinalkohol. Chem. Ber..

[27] Ahmed R., Lehrer M., Stevenson R. (1973). Synthesis of thomasidioic acid. Tetrahedron Lett..

[28] Ahmed R., Lehrer M., Stevenson R. (1973). Synthesis of thomasic acid. Tetrahedron.

[29] Wallis A.F.A. (1973). Oxidation of (*E*)- and (*Z*)-2,6-Dimethoxy-4-propenylphenol with ferric chloride—A facile route to the 2-aryl ethers of 1-Arylpropan-1,2-diols. Aust. J. Chem..

[30] Wallis A.F.A. (1973). Oxidative dimerization of methyl (*E*)-sinapate. Aust. J. Chem..

[31] Kumada Y., Naganawa H., Takeuchi T., Umezawa H., Yamashita K., Watanabe K. (1978). Biochemical activities of the derivatives of dehydrodicaffeic acid dilactone. J. Antibiot..

[32] Ahmed R., Schreiber F.G., Stevenson R., Williams J.R., Yeo H.M. (1976). Oxidative coupling of bromo- and iodo-ferulic acid derivatives: synthesis of (±)-veraguensin. Tetrahedron.

[33] Stevenson R., Williams J.R. (1977). Synthesis of tetrahydrofuran lignans, (±)-galbelgin and (±)-grandisin. Tetrahedron.

[34] Ralph J., Quideau S., Grabber J.H., Hatfield R.D. (1994). Identification and synthesis of new ferulic acid dehydrodimers present in grass cell walls. J. Chem. Soc. Perkin Trans. 1.

[35] Kumada Y., Takeuchi T., Umezawa H. (1977). Characterization of the dehydrodicaffeic acid dilactone-forming enzyme and the enzymic and chemical synthesis of this mushroom product. Agric. Biol. Chem..

[36] Jin X.L., Yang R.T., Shang Y.J., Dai F., Qan Y.P., Cheng L.X., Zhou B., Liu Z.L. (2010). Oxidative coupling of cinnamic acid derivatives and their radical-scavenging activities. Chin. Sci. Bull..

[37] Iguchi M., Nishiyama A., Eto H., Terada Y., Yamamura S. (1979). Anodic oxidation of 4-hydroxycinnamic acids. Chem. Lett..

[38] Cooper R., Gottlieb H.E., Lavie D., Levy E.C. (1979). Lignans from *Aegilops ovata* L. Synthesis of a 2,4- and 2,6-diaryl monoepoxylignanolide. Tetrahedron.

[39] Taylor E.C., Andrade J.G., Rall G.J.H., Steliou K., Jagdmann G.E., McKillop A. (1981). Thallium in Organic Synthesis. 60. 2,6-diaryl-3,7-dioxabicyclo[3.3.0]octane-4,8-dione lignans by oxidative dimerization of 4-alkoxycinnamic acids with thallium(III) trifluroacetate or cobalt(III) trifluoride. J. Org. Chem..

[40] Mori N., Watanabe H., Kitahara T. (2006). Simple and efficient asymmetric synthesis of furofuran lignans yangambin and caruilignan A. Synthesis.

[41] Setälä H., Pajunen A., Kilpelläinen I., Brunow G. (1994). Horse radish peroxidase-catalysed oxidative coupling of methyl sinapate to give diastereomeric spiro dimers. J. Chem. Soc. Perkin Trans. 1.

[42] Bunzel M., Ralph J., Kim H., Lu F., Ralph S.A., Marita J.M., Hatfield R.D., Steinhart H. (2003). Sinapate dehydrodimers and sinapate-ferulate heterodimers in cereal dietary fiber. J. Agric. Food Chem..

[43] Neudorffer A., Deguin B., el Ha C., Fleury M.-B., Largeron M. (2003). Electrochemical oxidative coupling of 4-hydroxycinnamic ester derivatives: A convenient methodology for the biomimetic synthesis of lignin precursors. Collect. Czechoslov. Chem. Commun..

[44] Zoia L., Bruschi M., Orlandi M., Tolppa E.-L., Rindone B. (2008). Assymetric biomimetic oxidations of phenols: the mechanism of the diastereo- and enantioselective synthesis of thomasidioic acid. Molecules.

[45] Cilliers J.J.L., Singleton V.L. (1991). Characterization of the products of non-enzymic autoxidative phenolic reactions in a caffeic acid model system. J. Agric. Food Chem..

[46] Maeda S., Masuda H., Tokoroyama T. (1995). Studies on the preparation of bioactive lignans by oxidative coupling reaction. IV. Oxidative coupling reaction of methyl (*E*)-3-(3,4-dihydroxy-2-methoxyphenyl)propenoate and lipid peroxidation inhibitory effects of the produced lignans. Chem. Pharm. Bull..

[47] Maeda S., Masuda H., Tokoroyama T. (1994). Studies on the preparation of bioactive lignans by oxidative coupling reaction. IΙ. Oxidative coupling reaction of methyl (*E*)-3-(4,5-dihydroxy-2-methoxyphenyl)propenoate and lipid peroxidation inhibitory effects of the produced lignans. Chem. Pharm. Bull..

[48] Agata I., Hatano T., Nishibe S., Okuda T. (1988). Rabdosiin, a new rosmarinic acid dimer with a lignan skeleton from *Rabdosia japonica*. Chem. Pharm. Bull..

[49] Agata I., Hatano T., Nishibe S., Okuda T. (1989). A tetrameric derivative of caffeic acid from *Rabdosia japonica*. Phytochemistry.

[50] Kashiwada Y., Nishizawa M., Yamagishi T., Tanaka T., Nonaka G., Cosentino L.M., Snider J.V., Lee K. (1995). Anti-AIDS agents, 18. Sodium and potassium salts of caffeic acid tetramers from Arnebia euchroma as anti-HIV agents. J. Nat. Prod..

[51] </b>Kashiwada Y., Bastow K.F., Lee K. (1995). Novel lignan derivatives as selective inhibitors of DNA topoisomerase II. Bioorg. Med. Chem. Lett..

[52] Bogucki D.E., Charlton J.L. (1997). A non-enzymatic synthesis of (*S*)-(−)-rosmarinic acid and a study of a biomimetic route to (+)-rabdosiin. Can. J. Chem..

[53] Reimann E., Pflug T. (1998). Synthese der enantiomeren (+)- and (−)-rosmarinsäuremethylester. Monatsch. Chem..

[54] Huang L.-J., Li C.-H., Lu Z.-M., Ma Z.-B., Yu D.-Q. (2006). Total synthesis and biological evaluation of (+)- and (−)-butyl ester of rosmarinic acid*.*. J. Asian Nat. Prod. Res..

[55] Aung H.T., Furukawa T., Nikai T., Niwa M., Takaya Y. (2011). Contribution of cinnamic acid analoues in rosmarinic acid to inhibition of snake venom induced hemorrhage. Bioorg. Med. Chem..

[56] Snyder S.A., Kontes F. (2009). Explorations into neolignan biosynthesis: Concise total syntheses of Helicterin B, Helisorin, and Helisterculin A from a common intermediate. J. Am. Chem. Soc..

[57] Exarchou V., Takis P.G., Malouta M., Vervoort J., Karali E., Troganis A.N. (2013). Four new depsides in *Origanum dictamnus* methanol extract. Phytochem. Lett..

[58] Ge F., Tang C.-P., Ye Y. (2008). Lignanamides and sesquiterpenoids from stems of Mitrephora thorelii. Helv. Chim. Acta.

[59] Daquino C., Rescifina A., Spatafora C., Tringali C. (2009). Biomimetic synthesis of natural and “unnatural” lignans by oxidative coupling of caffeic esters. Eur. J. Org. Chem..

[60] Lin H.-P., Lin C.-Y., Huo C., Su L.-C., Chuu C.-P. (2012). Anticancer effect of caffeic acid phenethyl ester. Pharmacologia.

[61] Maddaford S.P., Charlton J.L. (1993). A general asymmetric synthesis of (−)-α-dimethylretrodendrin and its diastereomers. J. Org. Chem..

[62] Broomhead A.J., Rahman M.M.A., Dewick P.M., Jackson D.E., Lucas J.A. (1991). Matairesinol as precursor of *Podophyllum* lignans. Phytochemistry.

[63] Cambie R.C., Craw P.A., Rutledge P.S., Woodgate P.D. (1988). Oxidative coupling of lignans III. Non-phenolic oxidative coupling of deoxypodorhizon and related compounds. Aust. J. Chem..

[64] Sarkanen K.V., Wallis A.F.A. (1973). Oxidative dimerization of methyl (*E*)-4-hydroxy-3,5-di-t-butylcinnamate with potassium ferricyanide. J. Chem. Soc. Perkin Trans. 1.

[65] Wang Q., Yang Y., Li Y., Yu W., Hou Z.H. (2006). An efficient method for the synthesis of lignans. Tetrahedron.

[66] Sugahara T., Yamauchi S., Kondo A., Ohno F., Tominaga S., Nakashima Y., Kishida T., Akiyama K., Maruyama M. (2007). First stereoselective synthesis of *meso*-secoisolariciresinol and comparison of its biological activity with (+) and (−) secoisolariciresinol. Biosci. Biotechnol. Biochem..

[67] Moon S.-S., Rahman A.A., Kim J.-Y., Kee S.-H. (2008). Hanultarin, a cytotoxic lignan as an inhibitor of actin cytoskeleton polymerization from the seeds of *Trichosanthes kirilowii*. Bioorg. Med. Chem..

[68] Lee E., Ahamed V.S.J., Kumar M.S., Rhee S.W., Moon S.-S., Hong I.S. (2011). Synthesis and evaluation of cytotoxic effect of hanultarin and its derivatives. Bioorg. Med. Chem. Lett..

[69] Ma C.-Y., Liu W.K., Che C.-T. (2002). Lignanamides and nonalkaloidal components of *Hyoscyamus niger* seeds. J. Nat. Prod..

[70] Τomosaka H., Chin Y.-W., Salim A.A., Keller W.J., Chai H., Kingborn A.D. (2008). Antioxidant and cytoprotective compounds from Berberis vulgaris (Barberry). Phytother. Res..

[71] Li D., Li W., Wang Q., Yang Z., Hou Z. (2010). Consize synthesis of cannabisin G. Bioorg. Med. Chem. Lett..

[72] Chioccara F., Poli S., Rindone B., Pilati T., Brunow G., Pietikäinen P., Setälä H. (1993). Regio- and Diastereo-selective synthesis of dimeric lignans using oxidative coupling. Acta Chem. Scand..

[73] Maeda S., Masuda H., Tokoroyama T. (1994). Studies on the preparation of bioactive lignans by oxidative coupling reaction. I. Preparation and lipid peroxidation inhibitory effect of benzofuran lignans related to schizotenuins. Chem. Pharm. Bull..

[74] Maeda S., Masuda H., Tokoroyama T. (1994). Studies on the preparation of bioactive lignans by oxidative coupling reaction. III. Synthesis of polyphenolic benzofuran and coumestan derivatives by oxidative coupling reaction of methyl (*E*)-3-(4-hydroxy-2-methoxyphenyl)propenoate and their inhibitory effect on lipid peroxidation. Chem. Pharm. Bull..

[75] Bolzacchini E., Brunow G., Meinardi S., Orlandi M., Rindone B., Rummakko P., Setala H. (1998). Enantioselective synthesis of a benzofuranic neolignan by oxidative coupling. Tetrahedron Lett..

[76] Ralph J., Garcia Conesa M.T., Williamson G. (1998). Simple preparation of 8–5 coupled diferulate. J. Agric. Food Chem..

[77] Pieters L., van Dyck S., Gao M., Bai R., Hamel E., Vlietinck A., Lemière G. (1999). Synthesis and biological evaluation of dihydrobenzofuran lignans and related compounds as potentia antitumor agents that inhibit tubulin polymerization. J. Med. Chem..

[78] Carunchio F., Crescenzi C., Girelli A.M., Messina A., Tarola A.M. (2001). Oxidation of ferulic acid by laccase: Identification of the products and inhibitory effect of some dipeptides. Talanta.

[79] Kuo Y.-H., Wu C.-H. (1996). Synthesis of 5-(3-hydroxypropyl)-7-methoxy-2-(3'-methoxy-4'-hydroxyphenyl)-3-benzo[*b*]furancarbaldehyde, a novel adenosine A_1_ receptor ligand from the root of *Salvia miltiorrhiza*. J. Nat. Prod..

[80] Chang J.Y., Chang C.-Y., Kuo C.-C., Chen L.-T., Wein Y.-S., Kuo Y.-H. (2004). Salvinal, a novel microtubule inhibitor isolated from Salvia miltiorrhizae Bunge (Danshen), with antimitotic activity in multi-drug-sensitive and-resistant human tumor cells. Mol. Pharmacol..

[81] Rakotondramanana D.L.A., Delomenede M., Baltas M., Duran H., Bedos-Belval F., Rasoanaivo P., Negre-Salvayre A., Gornitzka H. (2007). Synthesis of ferulic ester dimers, functionalization and biological evaluation as potential antiatherogenic and antiplasmodial agents. Bioorg. Med. Chem..

[82] Zhang A., Lu F., Sun R., Ralph J. (2009). Ferulate-coniferyl alcohol cross-coupled products formed by radical coupling reactions. Planta.

[83] Subbaraju G., Kavitha J., Rajasekhar D., Hsu F.-L., Cheng K.-T. (2007). Justicia lignans: Part 10—Synthesis of tiruneesiin, the first neolignan from Justicia species. Ind. J. Chem..

[84] Antus S., Bauer R., Gottsegen A., Seligmann O., Wagner H. (1987). Synthese von Americanin-D. Liebigs Ann. Chem..

[85] Antus S., Baitz-Gàcs E., Bauer R., Gottsegen A., Seligmann O., Wagner H. (1989). Regioselective synthesis of 2- and 3-aryl-1,4-benzodioxanes. Liebigs Ann. Chem..

[86] Antus S., Baitz-Gàcs E., Bauer R., Gottsegen A., Seligmann O., Wagner H. (1990). Total synthesis of cedrusin and its methyl ether. Liebigs Ann. Chem..

[87] Antus S., Gottsegen A., Kolonits P., Wagner H. (1989). Total synthesis of two naturally occurring neolignans of potential biological activity. Liebigs Ann. Chem..

[88] Bruschi M., Orlandi M., Rindone B., Rummakko P., Zoia L. (2006). Asymmetric biomimetic oxidations of phenols using oxazolidines as chiral auxiliaries: the enantioselective synthesis of (+)- and (−)-dehydrodiconiferyl alcohol. J. Phys. Org. Chem..

[89] Arrieta-Baez D., Stark R.E. (2006). Modeling suberization with peroxidase-catalyzed polymerization of hydroxycinnamic acids: Cross-coupling and dimerization reactions. Phytochemistry.

[90] Saliu F., Tolppa E.-L., Zoia L., Orlandi M. (2011). Horseradish peroxidase catalyzed oxidative cross-coupling reactions: The synthesis of “unnatural” dihydrobenzofuran lignans. Tetrahedron Lett..

[91] Nezbedovà L., Hesse M., Drandarov K., Werner C. (2001). New reagent for oxidative phenol coupling. The transformation of the monocyclic spermine base (*S*)-dihydroverbacine to the bicyclic alkaloid (*S*,*S*,*S*)-aphelandrine by cell free extract of barley seedlings. Tetrahedron Lett..

[92] Bird C.R., Smith T.A. (1981). The biosynthesis of coumarylagmantine in barley seedings. Phytochemistry.

[93] Varadaraju T.G., Hwu J.R. (2012). Synthesis of anti-HIV lithospermic acid by two divergent strategies. Org. Biomol. Chem..

[94] Jacobson R.M., Raths R.A. (1979). Total synthesis of heptamethyl lithospermate. J. Org. Chem..

[95] O’Malley S.J., Tan K.L., Watzke A., Bergman R.G., Ellman J.A. (2005). Total synthesis of (+)-lithospermic acid by asymmetric intramolecular alkylation via catalytic C-H bond activation. J. Am. Chem. Soc..

[96] Wang D.-H., Yu J.-Q. (2011). Highly convergent total synthesis of (+)-lithospermic acid via a late-stage intermolecular C-H olefination. J. Am. Chem. Soc..

[97] Fischer J., Savage G.P., Coster M.J. (2011). A concise route to dehydrobenzo[*b*]furans: Formal total synthesis of (+)-lithospermic acid. Org. Lett..

[98] Ly T.N., Shimoyamada M., Yamauchi R. (2006). Isolation and characterization of rosmarinic acid oligomers in *Celastrus hindsii* Benth leaves and their antioxidative activities. J. Agric. Food Chem..

[99] Lu F., Wei L., Azarpira A., Ralph J. (2012). Rapid synthesis of dehydrodiferulates via biomometic radical coupling reactions of ethyl ferulate. J. Agric. Food Chem..

[100] Agata I., Kusakabe H., Hatano T., Nishibe S., Okuda T. (1993). Melitric acids A and B, new trimeric caffeic acid derivatives from Meliss officinalis. Chem. Pharm. Bull..

[101] Lu Y., Foo L.Y. (1999). Rosmarinic acid derivatives from *Salvia officinalis*. Phytochemistry.

[102] Lu Y., Foo L.Y., Wong H. (1999). Sagecoumarin, a novel caffeic acid trimer from *Salvia officinalis*. Phytochemistry.

[103] Wang J., Xiong X., Feng B. (2013). Cardiovascular effects of salvianolic acid B. Evid. Based Complement. Alternat. Med..

[104] Zhao Y., Guo Y., Gu X. (2011). Salvianolic acid B, a potential chemoprotective agent, for head and necksquamous cell cancer. J. Oncol..

[105] Setälä H., Pajunen A., Rummakko P., Sipilä J., Brunow G. (1999). A novel spiro compound formed by oxidative cross coupling of methyl sinapate with a syringyl lignin model compound. A model system for the β-1 pathway in lignin biosynthesis. J. Chem. Soc. Perkin Trans. 1.

